# A revision of the ant genus *Mystrium* in the Malagasy region with description of six new species and remarks on *Amblyopone* and *Stigmatomma* (Hymenoptera, Formicidae, Amblyoponinae)

**DOI:** 10.3897/zookeys.394.6446

**Published:** 2014-03-31

**Authors:** Masashi Yoshimura, Brian L. Fisher

**Affiliations:** 1 Department of Entomology, California Academy of Sciences, Golden Gate Park, 55 Music Concourse Drive, San Francisco, California 94118, U.S.A.

**Keywords:** Major, minor, ergatoid queen, intercaste, intermediate, male, Madagascar, Comoros, Mayotte, mandible evolution, labral dentiform setae, genitalia, Amblyoponini, XMAS clade, *Onychomyrmex*, *Apomyrma*

## Abstract

The genus *Mystrium* is revised for the Malagasy region. Six species, *Mystrium barrybressleri* **sp. n.**, *Mystrium labyrinth* **sp. n.**, *Mystrium eques* **sp. n.**, *Mystrium mirror* **sp. n.**, *Mystrium shadow* **sp. n.**, and *Mystrium janovitzi* **sp. n.** are described as new. Two existing names, *Mystrium fallax* Forel and *Mystrium stadelmanni* Forel, are synonymized with *Mystrium voeltzkowi* Forel and *Mystrium mysticum* Roger, respectively. All recognized species, including species outside of the Malagasy region, are assigned to one of the three newly proposed species groups. The associations between existing names and males are reexamined, and males of eight of the ten Malagasy species are described or redescribed. The taxonomic history of *Mystrium* highlights the importance of using unique identifiers when designating type specimens and the use of deposited vouchers in phylogenetic and ecological studies. Keys to species for workers, queens, and males are provided. Furthermore, a neotype for *Mystrium mysticum* is designated, as well as lectotypes for *Mystrium camillae* Emery, *Mystrium rogeri* Forel, *Mystrium fallax* Forel, *Mystrium oberthueri* Forel, *Mystrium stadelmanni* Forel, and *Mystrium voeltzkowi* Forel. *Stigmatomma gingivale* (Brown) is reassigned to *Amblyopone* as **comb. rev.** and *Amblyopone awa* Xu & Chu, *Amblyopone kangba* Xu & Chu, *Amblyopone meiliana* Xu & Chu, and *Amblyopone zomae* Xu & Chu are transferred to the genus *Stigmatomma* as **comb. n.**

## Introduction

**Figure 1.**
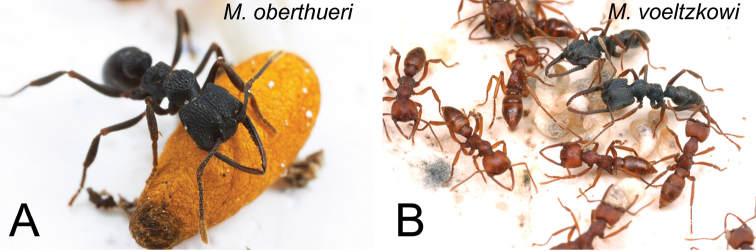
Colonies of *Mystrium*. **A** *Mystrium oberthueri* **B** *Mystrium voeltzkowi*. **A** a cocoon and a worker **B** larvae, workers, and ergatoid queens. Photos by Alex Wild.

The genus *Mystrium* Roger, 1862 was originally described from Madagascar, and ten species names were regarded as valid prior to this revision (Fig. 1). The Malagasy region has an incomparably abundant and rich *Mystrium* fauna with over half of the species endemic to the Malagasy region. After Roger established *Mystrium* with a single species, *Mystrium mysticum* Roger, 1862, Forel described five additional species in the Malagasy region between 1895 and 1899 (Forel 1895; 1897; 1899). The first record of *Mystrium* outside the Malagasy region was *Mystrium camillae* Emery, 1889 from Myanmar, and the second was *Mystrium silvestrii* Santschi, 1914 from Cameroon. Later Menozzi (1929) revised the genus *Mystrium*, including all six Malagasy species, *Mystrium camillae*, and *Mystrium silvestrii*, and provided a key for workers to all of these species. This revision (Menozzi 1929) was the last taxonomic work on the Malagasy *Mystrium*. A subspecies was later established under *Mystrium camillae*, *Mystrium camillae javana* Karavaiev, 1925; however, this subspecies was synonymized with *Mystrium camillae* by Brown (1960). Outside the Malagasy region, *Mystrium oculatum* Xu, 1998, was described from China, and *Mystrium leonie* Bihn & Verhaagh, 2007 and *Mystrium maren* Bihn & Verhaagh, 2007 from West Papua, Indonesia. *Mystrium oculatum* was synonymized with *Mystrium camillae* by Bihn and Verhaagh (2007).

Recent research in Madagascar demonstrated that the previous taxonomic works were insufficient to diagnose the species boundaries of *Mystrium* in the Malagasy region. Specimens collected from a single colony showed remarkable morphological variations within each existing species, and these variations rendered most published species-level diagnostic characters insufficient to delimit species. A reassessment of species boundaries was also needed in light of recent ecological and phylogenetic studies. Ecological studies reported various reproductive systems across *Mystrium* species, including typical winged queens, short-winged queens (Molet et al. 2009), small ergatoid queens (Bouchet et al. 2013; Molet et al. 2007a) (Fig. 1B) and intercaste anomalies intermediate between queen and worker (Molet et al. 2009; Molet et al. 2012). Ergatoid queens, short-winged queens and the intercastes were not described by Menozzi (1929). The genus *Mystrium* was included as an evolutionarily interesting lineage in most recent molecular phylogenetic papers on ants (Brady et al. 2006; Kück et al. 2011; Moreau and Bell 2013; Moreau et al. 2006; Ouellette et al. 2006; Saux et al. 2004), but no functional key was available for establishing identification of the ecological and molecular vouchers. The existing taxonomic system of *Mystrium* was inadequate to incorporate new scientific knowledge into existing species limits. The taxonomic system of the males was in even worse condition. Of the previous male descriptions for four of the ten valid species, only a male of *Mystrium voeltzkowi* Forel, 1897 has been precisely associated with conspecific workers. The other male descriptions, for *Mystrium mysticum*, *Mystrium rogeri*, and *Mystrium oberthueri*, were vague, insufficient, and as we conclude, wrongly associated.

In this study, we revise the species-level taxonomy for the genus *Mystrium* in the Malagasy region. Comprehensive morphological and distributional examinations using recently accumulated material enabled us to recognize ten species in this region; six species are new, and two existing names, *Mystrium fallax* and *Mystrium stadelmanni*, are synonymized with *Mystrium voeltzkowi* and *Mystrium mysticum*, respectively. We propose a new species-group system, and all recognized species, including extralimital species, are assigned to one of the three species groups. Both workers and queens of existing Malagasy species are redescribed with new diagnostic characters. Associations between existing names and males are reexamined, and the males of eight of the ten Malagasy species of *Mystrium* are described or redescribed. Identification keys to species for workers, queens, and males are provided. Furthermore, a neotype for *Mystrium mysticum* is designated, as well as lectotypes for *Mystrium camillae*, *Mystrium rogeri*, *Mystrium fallax*, *Mystrium oberthueri*, *Mystrium stadelmanni*, and *Mystrium voeltzkowi*. Though we do not include species outside of the Malagasy region in this revision, we provide taxonomic remarks on *Mystrium camillae* to better define species in the *camillae* group from the Malagasy region.

In addition to the species-level taxonomy of *Mystrium*, we also propose new genus-level diagnostic characters of *Mystrium*. In our previous work (Yoshimura and Fisher 2012), we proposed that the elongated mandible in the genus *Amblyopone* Erichson, 1842 evolved separately from that in the XMAS (*Xymmer* Santschi, 1914 + *Mystrium* + *Adetomyrma* Ward, 1994 + *Stigmatomma* Roger, 1859) clade; however, we did not propose any diagnostic characters to distinguish between *Amblyopone* and XMAS clade genera. In this study, based on the comparison between *Mystrium*, the other genera in the XMAS clade (Yoshimura and Fisher 2012), and *Amblyopone*, we consider the presence of the dentiform setae on the labrum as a diagnostic character in workers to distinguish the genus *Amblyopone* from genera in the XMAS clade.

## Materials and methods

### Abbreviations of depositories

BMNH The Natural History Museum (formerly British Museum, Natural History), London, U.K.

CASC California Academy of Sciences, San Francisco, California, U.S.A.

MCZC Museum of Comparative Zoology, Cambridge, Massachusetts, U.S.A.

MHNG Muséum d’Histoire Naturelle de la Ville de Genève, Geneva, Switzerland

MNHN Muséum National d’Histoire Naturelle, Paris, France

MSNG Museo Civico di Storia Naturale “Giacomo Doria”, Genova, Italy

NHMB Naturhistorisches Museum, Basel, Switzerland

PBTZ Parc Botanique et Zoologique de Tsimbazaza, Antananarivo, Madagascar

ZMHB Museum für Naturkunde der Humboldt-Universität zu Berlin, Berlin, Germany

**Materials.** The material on which this work is based was collected during arthropod surveys in Madagascar and the nearby islands in the Southwest Indian Ocean conducted by B. Fisher and the Malagasy ant team from the Madagascar Biodiversity Center in Antananarivo, Madagascar. Their work in the region includes more than 6,000 leaf litter samples, 4,000 pitfall traps, 1,000 Malaise trap collections, and 9,000 additional hand collection events throughout Madagascar from 1992 through 2013 (see Fisher 2005 for additional details). All non-type material examined is deposited in CASC and PBTZ.

We used a series of specimens collected from a single colony to determine the associations among queens, workers, and males and the extent of within-colony variation. When it was impossible to find all sexes and castes within the same colony sample, the associations based on morphological characters were confirmed by COI barcoding analysis (Fisher unpublished data).

**Methods.** Observations and dissections were carried out under stereoscopic microscopes (LEICA M125). Digital color images were created using a LEICA DFC425 digital camera. LEICA Application Suite software (ver. 3.8) was used for images, and a compound microscope (Leica DM4000M) and Nikon digital camera (DXm1200) and Helicon Focus version 4.10.2 software were used as well. The images were edited in Adobe Photoshop and Adobe Illustrator. Each imaged or dissected specimen is uniquely identified with a specimen-level unique identifier (e.g. CASENT0003099).

**Figure 2.**
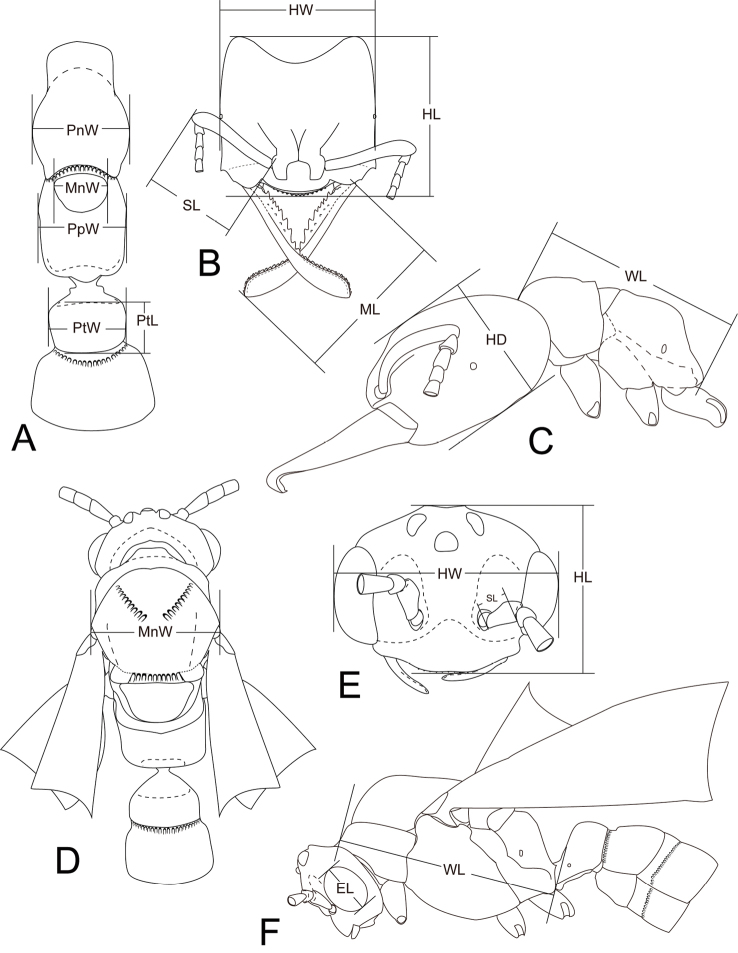
Measurements for *Mystrium* species. **A, B, C** worker **D, E, F** male. **A, D** body in dorsal view **B, E** head in full-face view **C, F** body in lateral view. Abbreviations are explained in Measurements and indices section.

**Measurements and indices.** The following measurements and indices (illustrated in Fig. 2) are cited in the text. The values are presented in mm.

Head length (HL), maximum length of head in full-face view between lines drawn across anterior margin of clypeus and posterior margin of head (worker, queen, male; including ocelli in male and queen).

Head width (HW), maximum width of head in full-face view, including eyes (worker, queen, and male).

Head depth (HD), maximum depth of head in lateral view measured perpendicular to full-face view plane (worker).

Mandible length (ML), maximum length of dorsal surface of the mandible (worker and queen).

Scape length (SL), length of scape excluding radicle (worker, queen, and male).

Eye length (EL), maximum length of eye measured in lateral view (queen and male).

Weber’s length of mesosoma (WL), maximum diagonal distance in lateral view, from base of anterior slope of pronotum to metapleural lobe (worker, queen, and male).

Pronotal width (PnW), maximum width of pronotum in dorsal view (worker).

Mesonotal width (MnW), maximum width of mesonotum in dorsal view (queen, and male).

Propodeal width (PpW), maximum width of propodeum in dorsal view (worker).

Petiolar length (PtL), maximum length of petiolar node in dorsal view (worker and queen).

Petiolar width (PtW), maximum width of petiolar node in dorsal view (worker and queen).

Cephalic index (CI), HW/HL ×100 (worker, queen, and male).

Mandible index (MI), ML/HW ×100 (worker and queen).

Scape index (SI), SL/HW ×100 (worker, queen, and male).

Eye index (EI), EL/HL ×100 (queen and male).

Mesonotal index (MnI), MnW/HW ×100 (queen and male).

Petiolar index (PtI), PtW/PtL ×100 (worker and queen).

**Figure 3.**
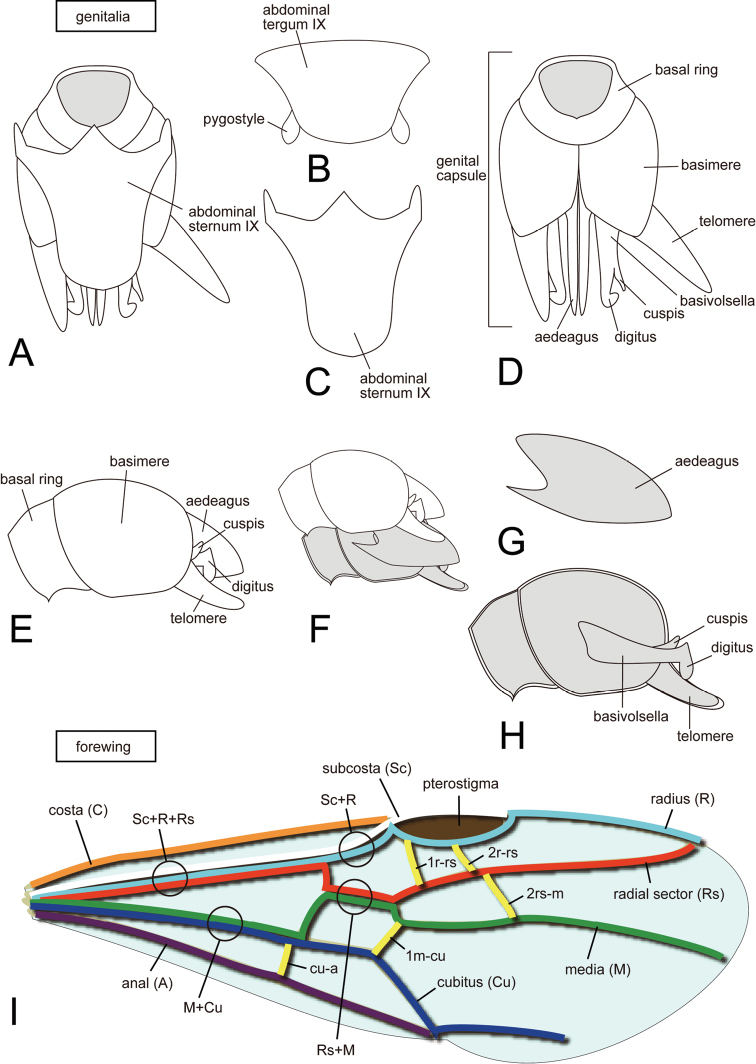
Terminology for male genitalia and forewing of ants. **A, D, E, F, H** genital capsule **B** abdominal tergum IX **C** abdominal sternum IX **G** aedeagus **I** forewing. **A, C, D** in ventral view **B, I** in dorsal view **E–H** in lateral view.

**Terminology.** Morphological terminology for workers and ergatoid queens follows Snodgrass (1935), Gauld and Bolton (1988), Bolton (1994), and Huber and Sharkey (1993); for alates it basically follows our previous works (Yoshimura and Fisher 2007: figs 1, 2; 2009: figs 1–21, 25–34; 2011: figs 1–3, 6, 11, 16–23, 34, 39, 40, 46–61, 76–81; 2012: figs 1–6, 8–13, 16). In addition to the above, the use of the term pygostyle follows Snodgrass (1941) while basimere and telomere (=harpago: Yoshimura and Fisher 2012) follows Snodgrass (1957); the terminology for wing venation in this study is a simplified Yoshimura and Fisher (2012) system based on the concepts in Wootton (1979) and Gauld and Bolton (1988); terminology for specialized conical setae on the anterior clypeal projections follows Ward (1994); the terminology of the genal tooth and the dentiform labral setae follow Keller (2011); the cinctus for the abdominal constriction between pre- and post-sclerites follows Serna and Mackay (2010). The terminology for genitalia and forewing used in this paper is illustrated in Figure 3 (Figs 3A–H for genitalia; Fig. 3I for forewing). Palpal formula is given as maxillary, labial (e.g. 4,3). We use anterior margin of the clypeus to refer to the margin where the clypeal conical setae are located. The distal end of the clypeus, however, may not always be visible in full-face view.

We replaced one genital term, telomere, with harpago in an earlier work (Yoshimura and Fisher 2012) to describe the apical subdivision of the paramere (Fig. 3D, E, and H). Both terms, telomere and harpago, follow the concept proposed by Snodgrass (1957), and are synonyms. Snodgrass originally proposed telomere as a name for the secondary distal division on the paramere, and harpago as another name for the telomere. We concur with Bolton (pers. com.) that because the telomere was consistently used through the manuscript in Snodgrass (1957) to indicate the distal secondary division of the paramere, it is the more appropriate term. In the same work, Snodgrass used the term harpago only once and did not explain the origin of the term. In this study, we use simplified names for wing veins. This simplification is not in conflict with the precise system used in our previous studies (Yoshimura and Fisher 2012). The simplified vein names are shown in Figure 3I, and the precise names were given in parentheses on figure 10c in Yoshimura and Fisher (2012).

**Terminology for caste names.** We classify females into three phenotypes: winged gyne, large wingless phenotype, and small phenotype with or without vestigial wings. Some species lack the winged gyne, and small phenotype acts as a reproductive cast; some species have intermediates between winged gyne and large phenotype. The presence or absence of each caste, and the role of each phenotype are consistent within species groups defined below, but vary among the species groups (Table 1). In this study, we use “queen” in reference only to the winged gyne; intercaste for intermediates between winged gyne and large wingless phenotype; major worker for the large wingless phenotype and minor worker for the small phenotype when the small phenotype does not function as a reproductive; worker for the large wingless phenotype and ergatoid queen for small phenotype when the small phenotype functions as a reproductive. The term female is used as a collective noun to indicate all female phenotypes. Males often display remarkable intra-specific variation in size and shape; however, no clear morphological division among those variations is recognized. Relationships between caste and species groups are given in Table 1.

**Terminology for geographical regions.** Terminology and definition for geographical regions follows Fisher (2010).

**Table 1.** Distribution of castes and phenotypes across species groups. Caste and the function of phenotypes vary across species groups. Caste names are explained in the Terminology section. Morphological characters defining phenotypes are given in the description of each species group.

	female				male
	winged gyne	intermediates	large wingless phenotype	small phenotype	
*camillae* species group	queen	-	major worker	minor worker	male
*mysticum* species group	queen	intercaste	major worker	minor worker	male
*voeltzkowi* species group	-	-	worker	ergatoid queen	male

## Results

### Genus Mystrium

http://species-id.net/wiki/Mystrium

Mystrium Roger, 1862: 245. Type-species: *Mystrium mysticum*, by monotypy.
Mystrium in Ponerinae: Mayr 1862: 715 [in key.]
Mystrium in Amblyoponinae, Amblyoponini: Forel 1893: 162; Bolton 2003: 42, 155.
Mystrium in Ponerinae, Amblyoponini: Emery 1895: 766.

#### Diagnosis of female.

The characters uniquely observed in *Mystrium* within the subfamily Amblyoponinae are given in italics.

**Figure 4.**
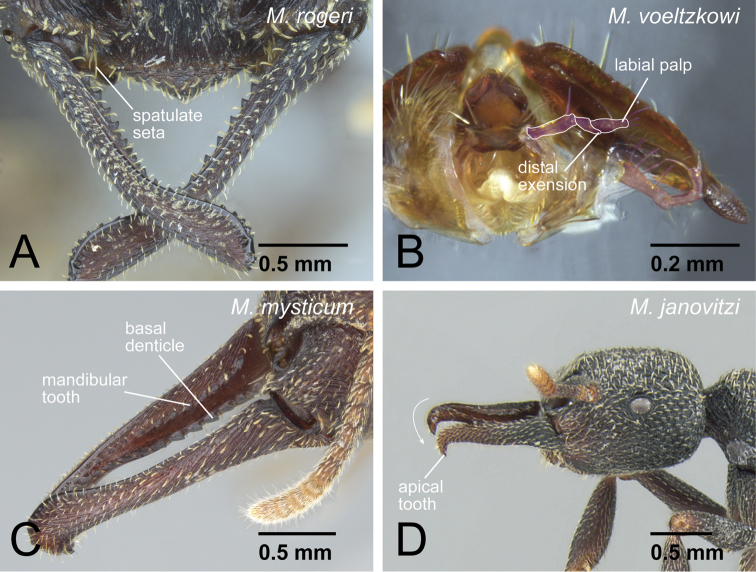
Generic diagnostic characters of *Mystrium*. **A** *Mystrium rogeri* (CASENT0001069) **B** *Mystrium voeltzkowi* (CASENT0317584) **C** *Mystrium mysticum* (CASENT0429965) **D** *Mystrium janovitzi* (CASENT0482696). **A, B, C** worker **D** ergatoid queen. **A** clypeus and mandible in oblique anterior view **B** mouthparts in oblique anterior view (left galea is omitted) **C** mandible in oblique lateral view **D** head in lateral view.

**Figure 5.**
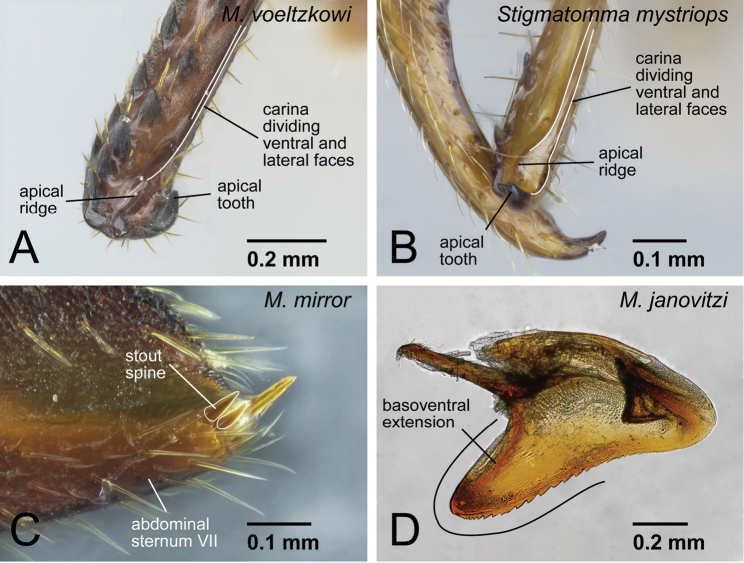
Generic diagnostic characters of *Mystrium* and the differences from *Stigmatomma*. **A** *Mystrium voeltzkowi* (CASENT0002075) **B** *Stigmatomma mystriops* (INB0003690672) **C** *Mystrium mirror* (CASENT0317582) **D** *Mystrium janovitzi* (CASENT0080644). **A, B, C** worker **D** male **A, B** mandible in oblique mesal view **C** abdominal segment VII in lateral view **D** aedeagus in lateral view.

1. Compound eye present.
2. Posterior margin of head strongly expands posteriorly on each side.
3. Anterior margin of clypeus with specialized conical setae.
4. *Spatulate setae present on clypeus mesal of mandible insertion* (Fig. 4A).
5. Labrum lacking small dentiform setae arranged horizontally.
6. Palpal formula 4,3.
7. *Distinct extension present on the distal edge of second labial palpomere* (Fig. 4B).
8. Mandible linear without a distinct basal angle.
9. Masticatory margin of mandible with two rows of projections: true mandibular teeth on dorsal row and a series of basal denticles on the ventral row; basal ventral denticles larger than true mandibular teeth except for apical teeth.
10. *At midlength of mandible, row of basal denticles arranged on ventral edge of mandibular shaft, distant from mandibular teeth* (Fig. 4C).
11. *Mandible twisted inward so that apical tooth is located ventrally* (Fig. 4D).
12. No basal large projection present on basal portion of mandibular inner margin.
13. *Ridge present on apical portion of mandible, which is originally dividing ventral and inner surfaces of mandible, inserting dorsally to the apical tooth* (Fig. 5A vs. 5B: *Stigmatomma mystriops* Brown, 1960, see comments).
14. Constriction between petiole and abdominal segment III present.
15. Cinctus 3, constriction between pre- and post-sclerites on abdominal segment IV, distinctly present.
16. *One or two stout spines present on posterior portion of abdominal sternum VII* (Fig. 5C).
17. Body surface with spatulate to squamose setae in some workers of all species.

#### Diagnosis of male.

The diagnostic characters uniquely observed in *Mystrium* within the subfamily Amblyoponinae are given in italics.

1. Frontal carinae present.
2. Anterior margin (=free margin) of clypeus with specialized conical setae.
3. Antenna consisting of 13 segments.
4. Mandible with single, blunt apical tooth.
5. Palpal formula 4,3.
6. Notaulus distinct or absent.
7. Mesepimeron often lacking distinct posterodorsal lobe (epimeral lobe).
8. Mesotibia with one or two spurs in most cases, rarely indistinct.
9. Metatibia with two spurs.
10. Distinct constriction present between petiole and abdominal segment III in dorsal view.
11. Abdominal segment IV with tergosternal fusion.
12. Pretergite of abdominal segment IV distinctly differentiated from posttergite, with the cinctus between them.
13. Pygostyles absent.
14. Distal margin of abdominal sternum IX convex.
15. Separation between basimere and telomere distinct.
16. Basal projection on cuspis well developed.
17. *Basoventral portion of the aedeagus in lateral view extended basally, distal margin of extension rounded* (Fig. 5D).
18. Serrate denticles present on basal portion of ventral margin of aedeagus in lateral view.
19. Pterostigma well developed on forewing.
20. Radial sector on forewing fully present.
21. Radial sector on forewing reaches costal margin.
22. 2r-rs on forewing connected with radial sector posterior to pterostigma.
23. 2rs-m present on forewing.
24. Position of cu-a on forewing variable, close to or far from junction between media and cubitus.
25. Radius present on hindwing.
26. 1rs-m present on hindwing.
27. Media on hindwing present apical to 1rs-m.

#### Comments on generic diagnosis.

The workers of the genus *Mystrium* can be distinguished easily from those of the other amblyoponine genera by their characteristic head shape and mandibles (Fig. 1). *Mystrium* has a wide head with posterior margin strongly concave, mandible linear and longer than head with rounded apex in full-face view, and two separated rows of “teeth” on the masticatory margin. In this study, we propose further detailed characters as the result of our comparative study. All *Mystrium* females share six characters unique to this genus which distinguish it from the other amblyoponine genera: spatulate setae on clypeus mesal of the mandible insertion (character 4: Fig. 4A), a distinct extension on the distal edge of the second segment on the labial palp (character 6: Fig. 4B), mandibular teeth distant from basal denticles (character 10: Fig. 4C), twisted mandible (character 11: Fig. 4D), a ridge on the apical portion of the mandible inserting dorsally to the apical tooth (character 13: 5A), and one or two lateral spines on abdominal sternum VII (character 16: 5C). Character 10, mandible teeth and basal denticles arranged in two rows (Fig. 4C), was used in Bolton (1994) to separate *Mystrium* from *Amblyopone* and *Stigmatomma*, both of which were in *Amblyopone* at that time. For character 11, Keller (2011: character 29) described the mandibular type as “torqued.” Although all amblyoponine genera share this character state, the mandible in *Mystrium* is unique: in females the mandible is further twisted so that the apical tooth of queens and minors is directed ventrally (Fig. 4D). For character 13, the ridge (= hump in Gronenberg et al. 1998) on the mandible functions as a pivot when the mandibles snap (Gronenberg et al. 1998). We confirmed that all females have this ridge, although it is uncertain if all females can snap their mandibles regardless of the shape of the apical tooth (see description for each species group). For character 16, we have confirmed that some species of *Stigmatomma* have three to nine lateral spines on abdominal sternum VII, while neither *Adetomyrma* nor *Xymmer* have this spine.

Brown (1960) described *Stigmatomma mystriops* (Brown, 1960) as a species that has specialized mandibular characters similar to *Mystrium*, e.g. teeth arranged in two separated rows, the presence of a ventral ridge on the subapical teeth, and a small apical tooth; however, these characters differ distinctly between *Stigmatomma mystriops* and *Mystrium* species. The mandibular teeth and basal denticles are consistently separated and these two rows are almost parallel, except for the apical portion of the mandible in *Mystrium*, while the separation exists only on the basal portion of the mandible in *Stigmatomma mystriops*. The ridge in *Mystrium* is the extension of a carina, which usually divides the lateral and ventral surfaces of the mandible, and the extension of the carina in *Mystrium* is inserted dorsally to the apical tooth (Fig. 5A). However, the ridge in *Stigmatomma mystriops* is developed independently from another carina along the ventral one mentioned above, which in *Stigmatomma mystriops* continues to the ventral margin of the apical tooth the same as a usual mandible of ants (Fig. 5B). The apical tooth in *Stigmatomma mystriops* is directed mesally, not ventrally as in *Mystrium*. In addition to the *Mystrium*-like characters (Brown 1960), we found two pairs of long setae on the anterior clypeal margin in *Stigmatomma mystriops*. These strange characters observed in *Stigmatomma mystriops* may be part of a specialized snapping mandible system.

In addition to characters unique to *Mystrium*, we discuss two generic characters (characters 5 and 9) that are useful for distinguishing amblyoponine genera. Neither character 5 nor 9 distinguish *Mystrium* from the other XMAS genera; however, both of these characters do distinguish *Mystrium* from *Amblyopone*.

**Figure 6.**
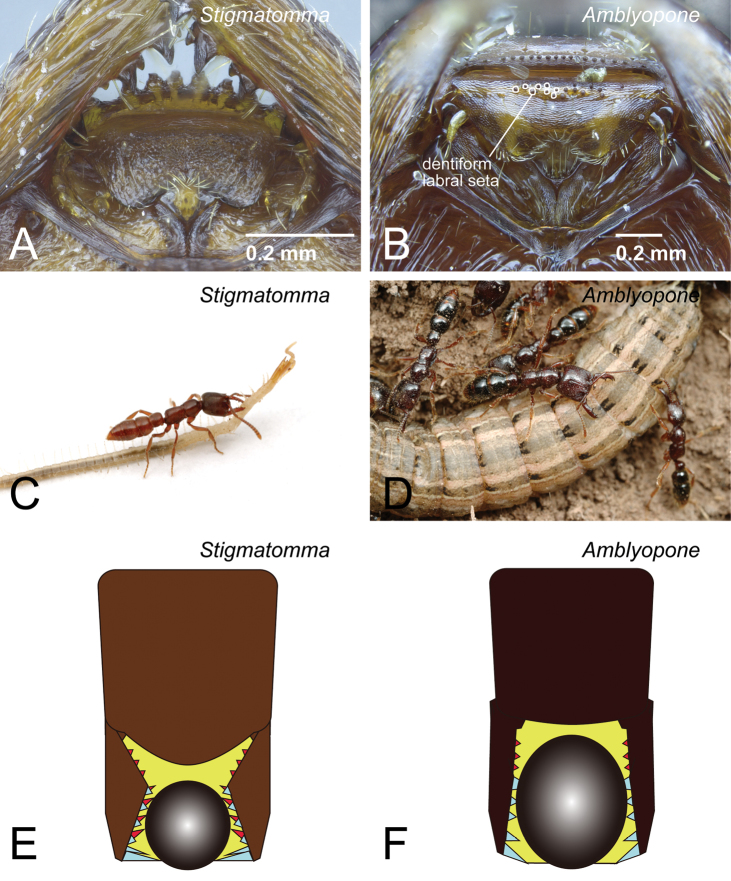
Differences in characters of the worker mandible and labrum between XMAS clade genera and *Amblyopone*. **A, C, E** XMAS clade genera (*Stigmatomma*) **B, D, F** *Amblyopone*. **A** *Stigmatomma pallipes* (CASENT0247077) **B** *Amblyopone australis* (CASENT0100424) **C** *Stigmatomma oregonense* **D** *Amblyopone australis*. **A, B** labrum **C, D** usage of the mandibles **E, F** pattern diagrams for mandibular use. **A** dentiform labral seta is absent **B** dentiform labral setae are present **C, E** the masticatory margin is the main functional part of the mandible in XMAS clade species **D, F** the whole mandibular shaft is the functional part of the mandible in *Amblyopone*. **C, D** Photos by Alex Wild.

Character 5 is provisionally proposed here to separate *Amblyopone* from genera in the XMAS clade. In the XMAS clade, the basal part of the labrum is lacking small dentiform setae (Fig. 6A). However, in *Amblyopone*, small dentiform setae are present on the labrum (Fig. 6B). Mandible character 9 proposed in Yoshimura and Fisher (2012) is unique to the XMAS clade. In our previous work, we described the unique evolution of the mandible in the XMAS clade. We presented as evidence the two-layered “teeth” on the masticatory surface of the mandible, which consists of true mandibular teeth on the dorsal row and apically extended basal denticles on the ventral row (Fig. 6E). We now update the mandibular character description because we have since observed in a few species of *Amblyopone* two-layered mandible dentition, such as in *Amblyopone* au02 (QMT152688). Even though two-layered mandible dentition was found in *Amblyopone*, as we described in character 9, the principal true mandibular teeth on the dorsal row are developed (Fig. 6F) larger than the basal denticles on the ventral row in *Amblyopone*, while the basal denticles are usually larger than true mandibular teeth on the masticatory margin with the exception of apical teeth in the XMAS clade (Fig. 6E).

The differences in mandibular characters between species in the XMAS clade and those in *Amblyopone* are associated with the parts of the mandible used for catching their prey. We assume that the masticatory margin is the main functional part of the mandible in XMAS clade species (Figs 6C, 6E), while the whole mandibular shaft is used for this purpose in *Amblyopone* (Figs 6D, 6F). The presence or absence of dentiform setae on the labrum (character 5: Figs 6B vs. 6A) supports our assumption. *Amblyopone* species probably “hold” prey using teeth along the whole shaft of the mandible (Fig. 6F), conical setae on the flat clypeus, and dentiform setae on the labrum (Fig. 6B); while species of *Stigmatomma* (in XMAS clade) “pinch” prey using their masticatory margin (distal part) of the mandible (Fig. 6E), and support it using anterior clypeal conical setae strongly extended anteriorly (Fig. 6A). When the mandibles of both groups elongated under these two use conditions, their morphology adapted differently. The mandibular shaft in *Amblyopone* became wider and stouter (Fig. 6F), while the basal denticles in XMAS clade became larger than true teeth and distinctly directed basally (Fig. 6E).

Brown (1960) was the first to propose that the dentiform setae on the labrum functioned as a structure to grip active prey. He attributed this character to Amblyoponini but our review suggest that dentiform setae on the basal part of the labrum (as in Fig. 6B) is restricted to three genera: *Amblyopone*, *Onychomyrmex* and *Apomyrma*. Ward (1994) reported that conical setae are present on the labrum in *Apomyrma stygia* Brown, Gotwald & Lévieux, 1971. Saux et al. (2004) described the presence of these setae in *Amblyopone australis* Erichson, 1842, *Amblyopone hackeri* Wheeler, 1927, *Amblyopone longidens* Forel, 1910, and *Stigmatomma gingivale* (Brown, 1960). Keller (2011) reported the presence of these setae in *Amblyopone australis*, *Amblyopone mercovichi* Brown, 1960, *Onychomyrmex doddi* Wheeler, 1916, and *Apomyrma stygia*, but their absence in *Concoctio concenta* Brown, 1974, and *Myopopone castanea* (Smith, 1860). Ward (1994) showed in his figure 9 labral setae in *Onychomyrmex* which is currently identified as *Onychomyrmex* au01 (PSW10030, CASENT0172779) though in Saux et al. (2004), this taxon is referred to as an undescribed species of *Amblyopone*. In addition to the above species, we found the presence of dentiform setae on the labrum in *Amblyopone michaelseni* Forel (CASENT0100441), *Amblyopone* au01 (CASENT0434465), and *Amblyopone* au02 (QMT152688). In *Apomyrma stygia* (CASENT0000077), the dentiform setae are present and distributed not only along the basal portion but also along the whole surface of the labrum; in addition, the conical setae on the anterior clypeal margin are absent. We also confirm the absence of dentiform setae on the labrum in *Prionopelta*, *Concoctio* and *Bannapone*.

Because of the presence of the dentiform labral setae, *Stigmatomma gingivale* is retransferred to *Amblyopone* as *Amblyopone gingivalis* Brown, 1960 comb. rev., although Yoshimura and Fisher (2012) transferred this species from *Amblyopone* to *Stigmatomma*. The dentiform labral setae in *Amblyopone gingivalis* are more abundant than those of *Amblyopone australis*, and covering on the distinct basal convexity on the labrum. In addition to the presence of the dentiform labral setae, true mandibular teeth larger than the basal ventral denticles are also present in *Amblyopone gingivalis* and provide an additional supportive character for this generic transfer.

On the other hand, on the basis of the mandible characters, *Amblyopone awa*, *Amblyopone kangba*, *Amblyopone meiliana*, and *Amblyopone zomae* are transferred to the genus *Stigmatomma* as *Stigmatomma awa* (Xu & Chu, 2012), comb. n., *Stigmatomma kangba* (Xu & Chu, 2012), comb. n., *Stigmatomma meilianum* (Xu & Chu, 2012), comb. n., and *Stigmatomma zomae* (Xu & Chu, 2012), comb. n., respectively. According to the original description (Xu and Chu 2012), the mandibular characters in figures 29, 34, and 52 show that *Stigmatomma zomae*, *Stigmatomma meilianum*, and *Stigmatomma awa* have typical forms observed in *Stigmatomma*, although we did not examine actual specimens of any of these four Chinese species.

The male diagnostic characters used in this revision follow those in Yoshimura and Fisher (2012). All worker diagnostic characters are applicable to both alate and ergatoid queens.

### Species groups in *Mystrium* and list of species

In this study, we divide the species of *Mystrium* into the three species groups listed below. The subdivisions of the genus are useful because of the difficulty of species-level identification.

***camillae* species group:** Alate queen present (Table 1). Dorsal surface of pronotum covered with regular reticulation in worker. Second maxillary palpomere shorter than the third in the Malagasy species. Labrum in females gently convex but without median longitudinal carina. Two lateral spines present on abdominal sternum VII in females. Whole vertex sculptured and not smooth in worker. Petiole in dorsal view in females relatively wide (PtI>210 in queen; PtI>180 in worker). Known from the Afrotropical, Malagasy, Indomalaya, and Australian regions.

***mysticum* species group:** Alate queen present (Table 1). Dorsal surface of pronotum covered with somewhat irregular striae, and central part of pronotal dorsum covered with fine striae in worker. Second maxillary palpomere longer than the third. Labrum in females with longitudinal carina and strongly convex on its central portion. Single lateral spine present on abdominal sternum VII in females. Ventral area of vertex in females not sculptured, smooth. Petiole in dorsal view in females relatively long (PtI<189 in queen; PtI<183 in worker). Known only from the Malagasy region.

***voeltzkowi* species group:** Only ergatoid queen, which is relatively smaller than worker, present; alate queen absent (Table 1). Dorsal surface of pronotum covered with somewhat irregular longitudinal striae in worker. Second maxillary palpomere longer than the third. Labrum in females gently convex but without median longitudinal carina. Two lateral spines present on abdominal sternum VII in females. Whole vertex in females sculptured and not smooth. Petiole in dorsal view relatively long (PtI<195 in queen; PtI<180 in worker). Known only from the Malagasy region.

A list of *Mystrium* species is given below. Sexes and castes known in each species are given in brackets as: w, worker; aq, alate queen; eq, ergatoid queen; m, male. In addition to the species listed below, we recognize two undetermined morphospecies in the collection and provide notes for these taxa in the remarks of the *camillae* species group. The associations between the species names and the species codes used in previous papers are also discussed in the remarks for each species.

*camillae* species group
  ***Mystrium barrybressleri* sp. n.** [w, aq, m] Madagascar
  ***Mystrium camillae*** Emery, 1889 [w, aq, m] South-East Asia, Australia
  =*Mystrium javana* Karavaiev, 1925 [w: minor]
  =*Mystrium oculatum* Xu, 1998 [w, aq]
  ***Mystrium labyrinth* sp. n.** [w, aq] Madagascar
  ***Mystrium leonie*** Bihn & Verhaagh, 2007 [w] Indonesia
  ***Mystrium maren*** Bihn & Verhaagh, 2007 [w] Indonesia
  ***Mystrium silvestrii*** Santschi, 1914 [w] Africa
*mysticum* species group
  ***Mystrium mysticum*** Roger, 1862 [w, aq, m] Madagascar, Comoros
  =*Mystrium stadelmanni* Forel, 1895 **syn. n.**
  ***Mystrium rogeri*** Forel, 1899 [w, aq, m] Madagascar, Comoros
*voeltzkowi* species group
  ***Mystrium eques* sp. n.** [w, eq] Madagascar
  ***Mystrium janovitzi* sp. n.** [w, eq, m] Madagascar
  ***Mystrium mirror* sp. n.** [w, eq, m] Madagascar
  ***Mystrium oberthueri*** Forel, 1897 [w, eq, m] Madagascar
  ***Mystrium shadow* sp. n.** [w, eq, m] Madagascar
  ***Mystrium voeltzkowi*** Forel, 1897 [w, eq, m] Madagascar, Mayotte
  =*Mystrium fallax* Forel, 1897 [eq] **syn. n.**

### Key to species of *Mystrium* in the Malagasy region

#### Workers and queens

**Table:** 

1	Wings present and well developed. Wing sclerites fully developed even with wings removed (Fig. 7A). Both anterior and lateral ocelli distinct (Fig. 7C)	2
–	Wings absent or vestigial. Wing sclerites undeveloped or incomplete (Fig. 7B). Lateral ocelli absent (Fig. 7D)	5
2	Labrum with distinct longitudinal ridge on its middle portion (Fig. 8A). Distal portion of abdominal sternum VII with single pair of stout spines (Fig. 8C). Body size relatively large (HW>1.9 mm)	3 (mysticum species group, part)
–	Labrum without distinct longitudinal ridge on its middle portion (Fig. 8B). Distal portion of abdominal sternum VII with two pairs of stout spines (Fig. 8D). Body size relatively small (HW<1.7 mm)	4 (*camillae* species group, part)
3	Setae (=hairs) on dorsal surface of head and pronotum simple (Fig. 9A). Anterior margin of clypeus convex (Fig. 9C)	*Mystrium mysticum* Roger (queen and intercaste)
–	Setae (=hairs) on dorsal surface of head and pronotum spatulate (Fig. 9B). Anterior margin of clypeus straight or weakly concave (Fig. 9D)	*Mystrium rogeri* Forel (queen and intercaste)
4	Petiole short in dorsal view, 0.5× length of abdominal segment III (Fig. 10A). Setae on pronotal dorsum narrow and sharp on its apex. First flagellomere (third segment of antenna) distinctly shorter than pedicel (second segment of antenna) (Fig. 10C). Body yellow to yellowish brown	*Mystrium barrybressleri* sp. n. (queen)
–	Petiole long in dorsal view, 0.7× length of abdominal segment III (Fig. 10B). Setae on pronotal dorsum spatulate and dull on its apex. First flagellomere as long as pedicel (Fig. 10D). Body blackish brown	*Mystrium labyrinth* sp. n. (queen)
5	Ventral half of vertex unsculptured, smooth (Fig. 11A). Labrum with distinct longitudinal ridge on its middle portion (as in Fig. 8A). Distal portion of abdominal sternum VII with single pair of stout spines (Fig. 8D). Anterior ocellus sometimes present	6 (*mysticum* species group, worker and intercaste)
–	Ventral half of vertex sculptured (Fig. 11B). Labrum without distinct longitudinal ridge on its middle portion (as in Fig. 8B). Distal portion of abdominal sternum VII with two pairs of stout spines (Fig. 8D). Ocelli absent	7
6	Anterior margin of clypeus convex (as in Fig. 9C). Among same-sized individuals, combination of following characters usually observed: sculpture relatively shallow and weak on lateral surface of pronotum; difference in width of dorsal surface of mandible larger between mandibular shaft (narrowest part) and distal portion (widest part) (Fig. 12A); petiole in dorsal view relatively wider	*Mystrium mysticum* Roger (worker and intercaste)
–	Anterior margin of clypeus straight or weakly concave (as in Fig. 9D). Among same sized individuals, combination of following characters usually observed: sculpture relatively deep and strong on lateral surface of pronotum; difference in width of dorsal surface of mandible smaller between mandibular shaft (narrowest part) and distal portion (widest part) (Fig. 12B); petiole in dorsal view relatively narrower	*Mystrium rogeri* Forel (worker and intercaste)
7	Head dorsum and pronotum strongly to weakly and regularly reticulate (Fig. 13A). No deep longitudinal furrow on midline of pronotum. Second maxillary palpomere shorter than third (Fig. 13C)	8 (*camillae* group, worker)
–	Head dorsum and pronotum finely to roughly sculptured longitudinally, but not regularly reticulate (Fig. 13B). Pronotum often with deep longitudinal furrow along midline. Second maxillary palpomere longer than third (Fig. 13D)	9 (*voeltzkowi* group)
8	First flagellomere segment of antenna distinctly shorter than pedicel (as in Fig. 10C). Genal tooth relatively short, distinctly failing to reach to anterior margin of lateral lobe of clypeus (Fig. 14A). No carina present on dorsal edge of metapleural gland bulla. Body size relatively small (HW<1.50 mm). Petiole relatively wider in dorsal view (PtI>195). Body yellowish brown to reddish brown	*Mystrium barrybressleri* sp. n. (worker)
–	First flagellomere as long as pedicel (as in Fig 10D). Genal tooth relatively long, almost reaching anterior margin of lateral lobe of clypeus (Fig. 14B). Distinct carina present on dorsal edge of metapleural gland bulla. Body size relatively large (HW>1.70 mm). Petiole relatively narrower in dorsal view (PtI<185). Body reddish brown to black	*Mystrium labyrinth* sp. n. (worker)
9	Apical tooth strongly sclerotized, its apex darker than basal portion, strongly curving inward (Fig. 15A)	10 (*voeltzkowi* group, worker)
–	Apical tooth moderately sclerotized, its apex same color as basal portion, straight and directed ventrally (Fig. 15B)	15 (*voeltzkowi* group, ergatoid queen)
10	With head in posterior view, posterior face of vertex forming right or even acute angle with its dorsal face on median line, on lateral portion of vertex as steep as median portion (Fig. 16A)	11
–	With head in posterior view, posterior face of vertex forming blunt angle with its dorsal face on median line, lateral portion of vertex distinctly steeper than median portion (Fig. 16B)	13
11	Dorsal surface of pronotum without strong, deep, and thick longitudinal striae (Fig. 17A). Genal tooth undeveloped, distinctly fails to reach anterior margin of lateral lobe of clypeus in full-face view (Fig. 17C). Clypeal conical setae extremely small and short (Fig. 17C). Metanotal groove indistinct in lateral view. Mesonotum often not differentiated from propodeum in dorsal view	*Mystrium janovitzi* sp. n. (worker)
–	Dorsal surface of pronotum with strong, deep, and thick longitudinal striae (Fig. 17B). Genal tooth distinctly developed, almost reach to anterior margin of lateral lobe of clypeus in full-face view (Fig. 17D). Clypeal conical setae long (Fig. 17D). Metanotal groove shallow but distinct in lateral view. Mesonotum always clearly differentiated from propodeum in dorsal view	12
12	Anterior margin of clypeus straight (Fig. 18A). First flagellomere longer than pedicel (Fig. 18C). With mandible in dorsal view, width of visible surface on distal portion almost the same as on mandibular shaft (Fig. 18E)	*Mystrium oberthueri* Forel (worker)
–	Anterior margin of clypeus strongly convex (Fig. 18B). First flagellomere as long as pedicel (Fig. 18D). With mandible in dorsal view, visible surface on distal portion slightly wider than on mandibular shaft (Fig. 18F)	*Mystrium eques* sp. n. (worker)
13	Anterior margin of clypeus strongly convex with long conical setae, of which median pair usually larger than adjacent pair (Fig. 19A)	*Mystrium shadow* sp. n. (worker)
–	Anterior margin of clypeus almost straight, with short conical setae, of which median pair usually smaller than adjacent pair (Fig. 19B)	14
14	Genal tooth relatively short, reaching or slightly exceeding basal line of lateral clypeal lobe (Fig. 20A). Eye relatively larger than in individuals of *Mystrium voeltzkowi* of same body size (Fig. 20C). Metapleural gland bulla weakly developed, posterior margin of propodeal declivity almost straight (Fig. 20E)	*Mystrium mirror* sp. n. (worker)
–	Genal tooth relatively long, reaching to about half of lateral clypeal lobe (Fig. 20B). Eye relatively smaller than *Mystrium mirror* in individuals of same body size (Fig. 20D). Metapleural gland bulla strongly developed, posterior margin of propodeal declivity distinctly convex posteriorly on its ventral side (Fig. 20F)	*Mystrium voeltzkowi* Forel (worker)
15	With head in posterior view, posterior face of vertex forming right or even acute angle with its dorsal face on median line, lateral portion of vertex as steep as on median portion (Fig. 21A)	16
–	With head in posterior view, posterior face of vertex forming blunt angle with its dorsal face on median line, lateral portion of vertex distinctly steeper than median portion (Fig. 21B)	18
16	Anterior margin of clypeus strongly convex (Fig. 22A)	*Mystrium eques* sp. n. (ergatoid queen)
–	Anterior margin of clypeus almost straight (Fig. 22B)	17
17	Clypeal conical setae extremely short (Fig. 23A). First flagellomere as long as pedicel (Fig. 23C)	*Mystrium janovitzi* sp. n. (ergatoid queen)
–	Clypeal conical setae moderately long (Fig. 23B). First flagellomere longer than pedicel (Fig. 23D)	*Mystrium oberthueri* Forel (ergatoid queen)
18	Metapleural gland bulla extremely well developed, expanding above propodeal spiracle (Fig. 24A). Propodeal declivity in lateral view strongly convex posteriorly on its ventral two thirds	*Mystrium voeltzkowi* Forel (ergatoid queen)
–	Metapleural gland bulla weakly to moderately developed, not expanding above propodeal spiracle (Fig. 24B). Propodeal declivity in lateral view usually straight or convex posteriorly on its ventral third	19
19	Setae on pronotal dorsum almost simple, narrowing distally, with sharp apex (Fig. 25A). Anterolateral corner of head usually without spine, but sometimes with small spine. Vestigial wings often unclear and unrecognizable, or existing as quite small appendage (Fig. 25C)	*Mystrium mirror* sp. n. (ergatoid queen)
–	Setae on pronotal dorsum spatulate, widened distally and with sharp apex (Fig. 25B). Anterolateral corner of head with small spine. Vestigial wings clearly recognizable as appendages (Fig. 25D)	*Mystrium shadow* sp. n. (ergatoid queen)

**Figure 7.**
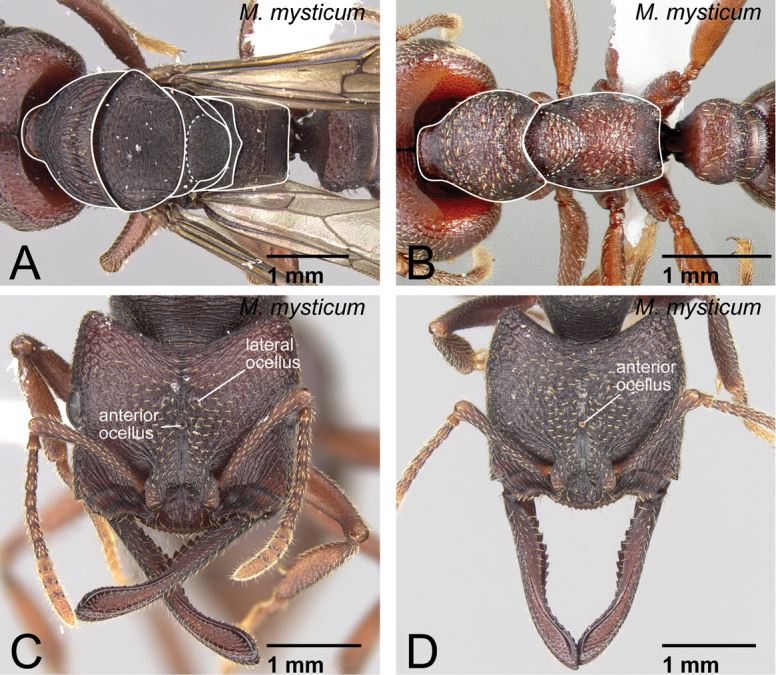
Female castes of *Mystrium mysticum*. **A, C** alate queen (CASENT0429962) **B** worker (CASENT0429955) **D** intercaste (CASENT0429959). **A, B** mesosoma in dorsal view **C, D** head in full-face view.

**Figure 8.**
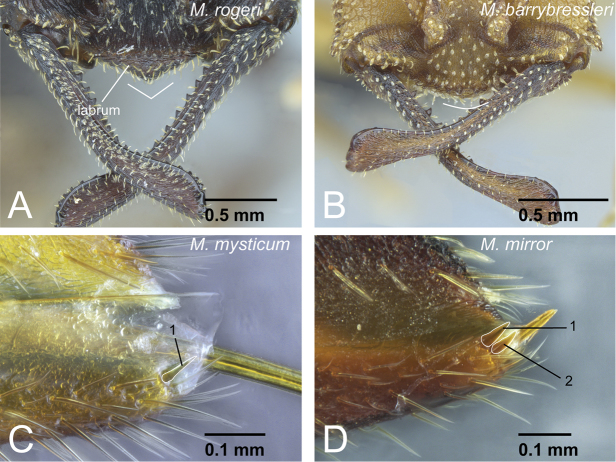
Female diagnostic characters of *mysticum* species group. **A** *Mystrium rogeri* (CASENT0001069) **B** *Mystrium barrybressleri* (CASENT0045009) **C** *Mystrium mysticum* (CASENT0003808) **D** *Mystrium mirror* (CASENT0317582). **A–D** worker. **A, B** mandible in oblique anterior view **C, D** abdominal segment VII in lateral view.

**Figure 9.**
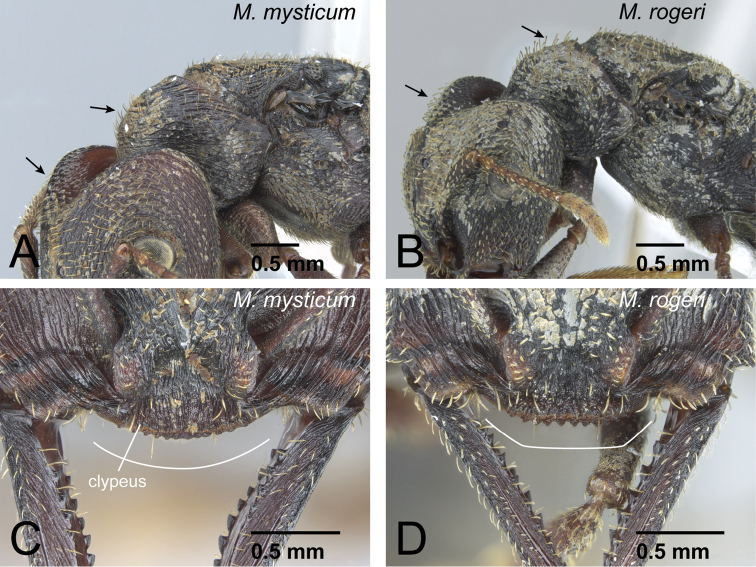
Queens of *mysticum* species group. **A, C** *Mystrium mysticum* (CASENT0429983) **B, D** *Mystrium rogeri* (CASENT0001070). **A, B** head to mesonotum in oblique lateral view **C, D** clypeus in oblique full-face view.

**Figure 10.**
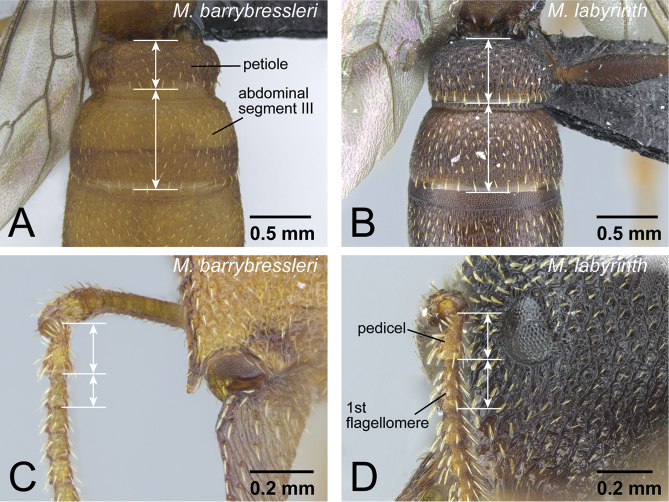
Queens of *camillae* species group. **A, C** *Mystrium barrybressleri* (CASENT0145109) **B, D** *Mystrium labyrinth* (CASENT0084950). **A, B** petiole and abdominal segment III in dorsal view **C, D** pedicel and flagellum in lateral view.

**Figure 11.**
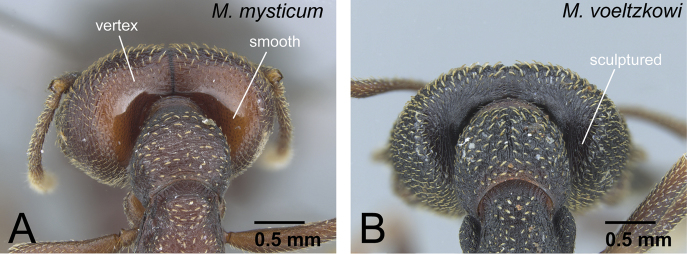
Vertex of *Mystrium* workers. **A** *Mystrium mysticum* (CASENT0429965) **B** *Mystrium voeltzkowi* (CASENT0002075).

**Figure 12.**
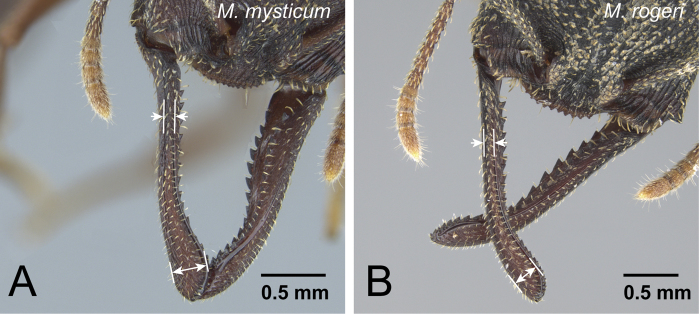
Mandible of females of *mysticum* species group. **A** *Mystrium mysticum* (CASENT0429965) **B** *Mystrium rogeri* (CASENT0001069).

**Figure 13.**
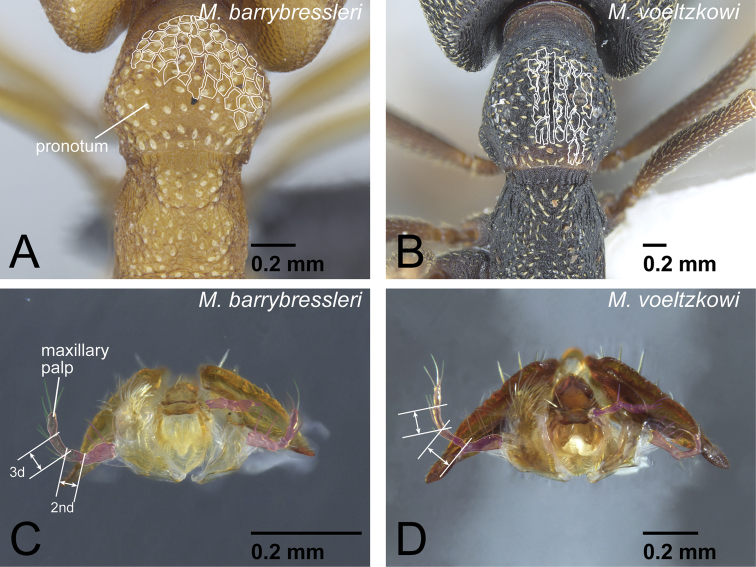
Worker diagnostic characters of *camillae* species group. **A** *Mystrium barrybressleri* (CASENT0045009) **B** *Mystrium voeltzkowi* (CASENT0002075) **C** *Mystrium barrybressleri* (CASENT0047970) **D** *Mystrium voeltzkowi* (CASENT0317584). **A–D** worker. **A, B** pronotum in dorsal view **C, D** mouthparts in oblique lateral view.

**Figure 14.**
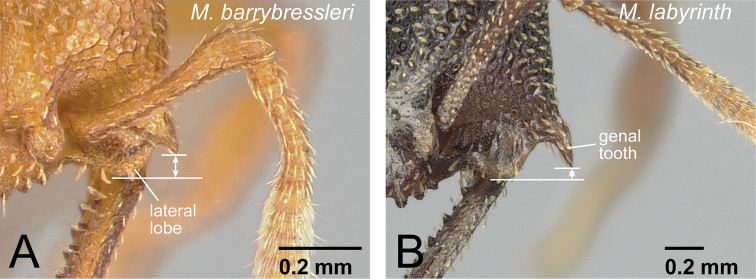
Head of workers in *camillae* species group. **A** *Mystrium barrybressleri* (CASENT0006081) **B** *Mystrium labyrinth* (CASENT0003281).

**Figure 15.**
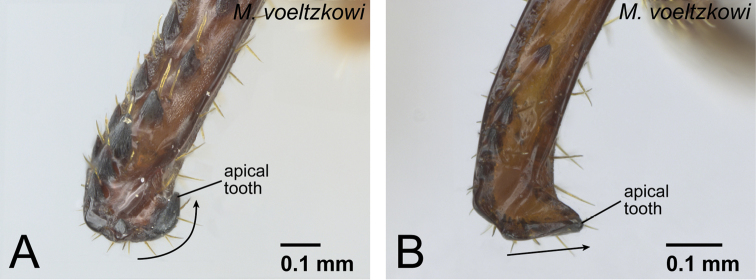
Distal portion of the mandible of *Mystrium voeltzkowi* in mesal view. **A** worker (CASENT0002075) **B** ergatoid queen (CASENT0002074).

**Figure 16.**
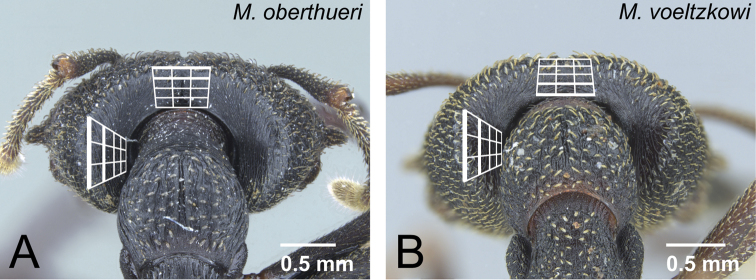
Vertex of workers in *voeltzkowi* species group. **A** *Mystrium oberthueri* (CASENT0499569) **B** *Mystrium voeltzkowi* (CASENT0002075). **A, B** head in posterior view.

**Figure 17.**
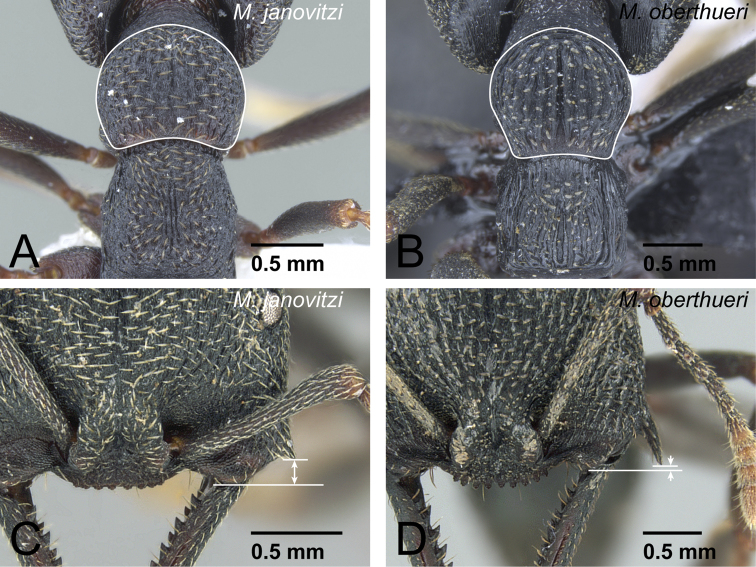
Differences in worker morphology between *Mystrium janovitzi* and *Mystrium oberthueri*. **A** *Mystrium janovitzi* (CASENT0006082) **B** *Mystrium oberthueri* (CASENT0499569) **C** *Mystrium janovitzi* (CASENT0482698: holotype) **D** *Mystrium oberthueri* (CASENT0496066). **A, B** mesosoma in dorsal view **C, D** clypeus and genal tooth in full-face view.

**Figure 18.**
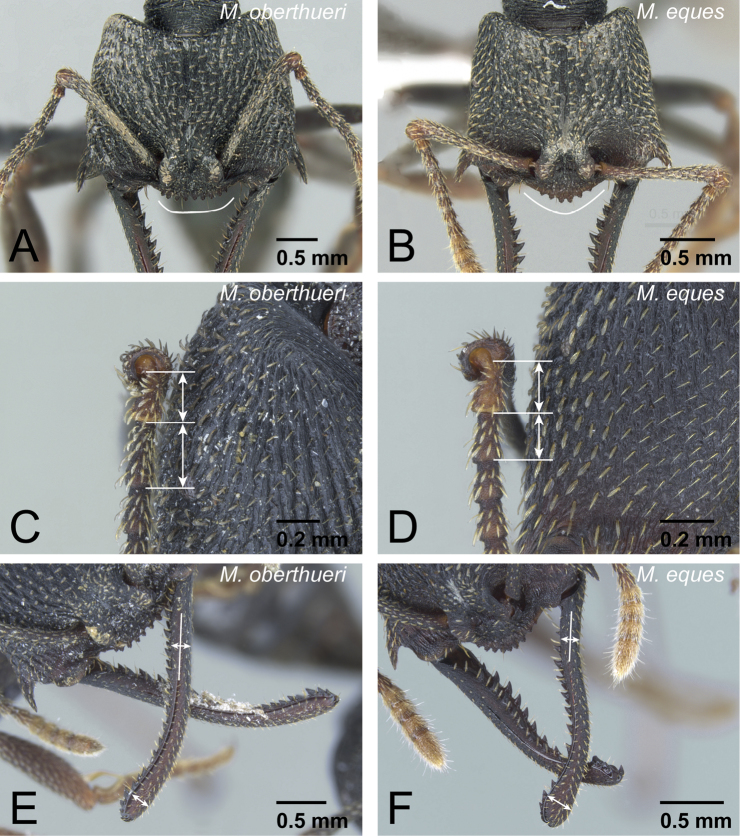
Differences in worker morphology between *Mystrium oberthueri* and *Mystrium eques*. **A** *Mystrium oberthueri* (CASENT0496066) **B** *Mystrium eques* (CASENT0418314: holotype) **C, E** *Mystrium oberthueri* (CASENT0499569) **D, F** *Mystrium eques* (CASENT0317399). **A, B** head in full-face view **C, D** pedicel and flagellum in lateral view **E, F** mandible in dorsal view.

**Figure 19.**
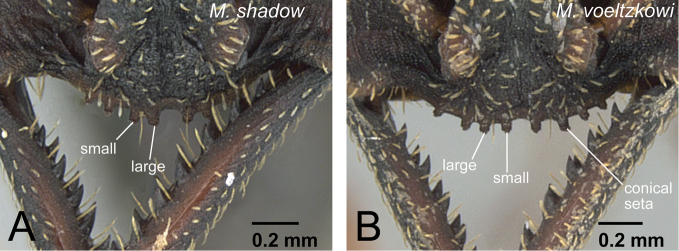
Clypeus of workers in *voeltzkowi* species group. **A** *Mystrium shadow* (CASENT0318933: holotype) **B** *Mystrium voeltzkowi* (CASENT0002075).

**Figure 20.**
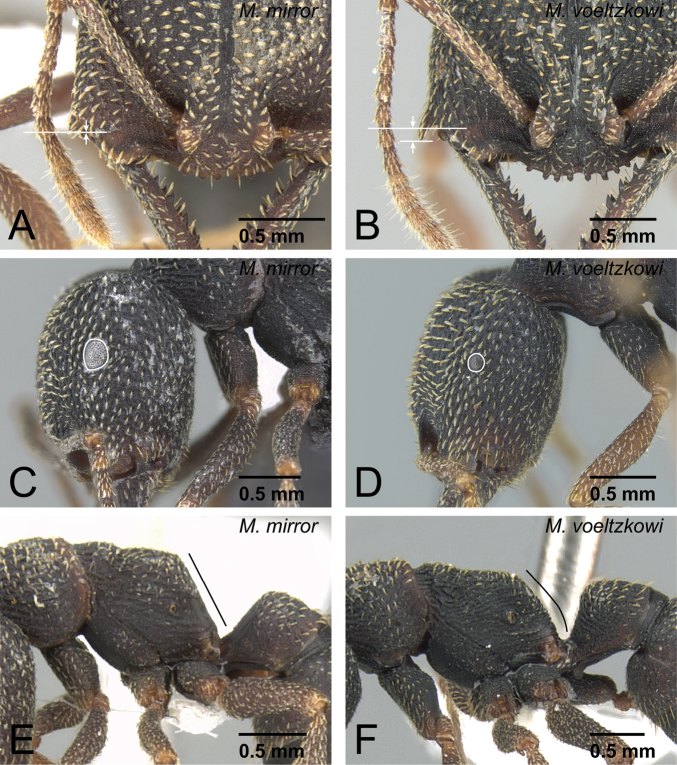
Differences in worker morphology between *Mystrium mirror* and *Mystrium voeltzkowi*. **A, E** *Mystrium mirror* (CASENT0429897) **B, D, F** *Mystrium voeltzkowi* (CASENT0002075) **C** *Mystrium mirror* (CASENT0002340). **A, B** head in full-face view **C, D** head in lateral view **E, F** mesosoma and petiole in lateral view.

**Figure 21.**
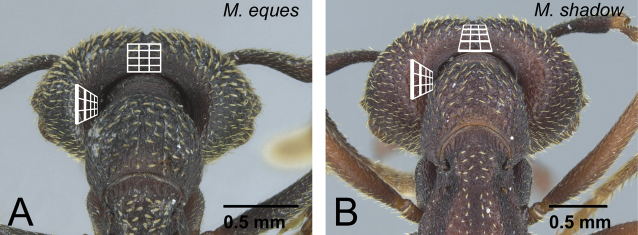
Vertex of ergatoid queens in *voeltzkowi* species group. **A** *Mystrium eques* (CASENT0418311) **B** *Mystrium shadow* (CASENT0077647: paratype). **A, B** head in posterior view.

**Figure 22.**
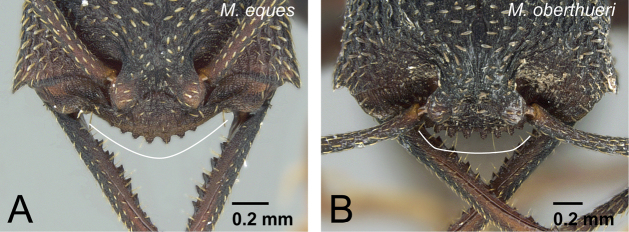
Clypeus of ergatoid queens in *voeltzkowi* species group. **A** *Mystrium eques* (CASENT0418317: paratype) **B** *Mystrium oberthueri* (CASENT0496064). **A, B** head in full-face view.

**Figure 23.**
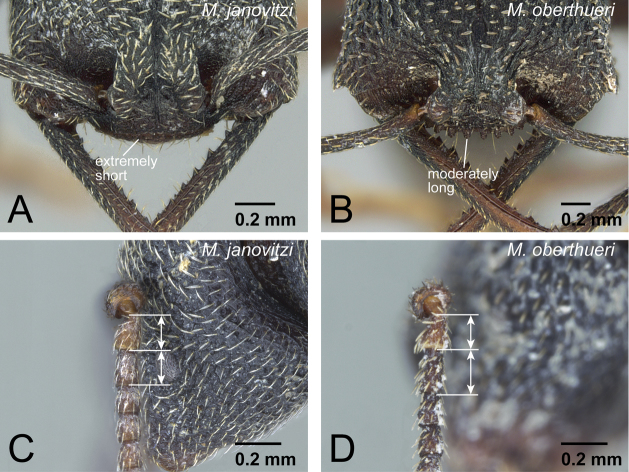
Differences in ergatoid queen characters between *Mystrium janovitzi* and *Mystrium oberthueri*. **A** *Mystrium janovitzi* (CASENT0482696) **B** *Mystrium oberthueri* (CASENT0496064) **C** *Mystrium janovitzi* (CASENT0003302) **D** *Mystrium oberthueri* (CASENT0095743). **A, B** head in full-face view **C, D** pedicel and flagellum in lateral view.

**Figure 24.**
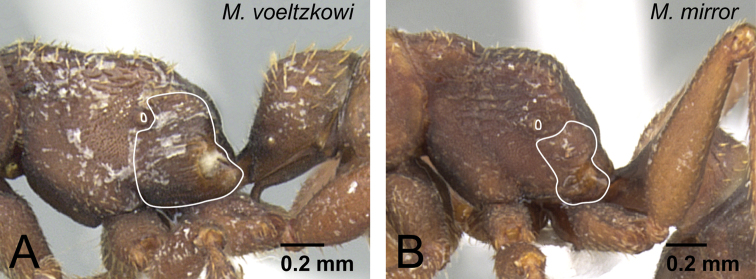
Propodeum of ergatoid queens of *Mystrium voeltzkowi* and *Mystrium mirror* in lateral view. **A** *Mystrium voeltzkowi* (CASENT0002092) **B** *Mystrium mirror* (CASENT0318940: paratype).

**Figure 25.**
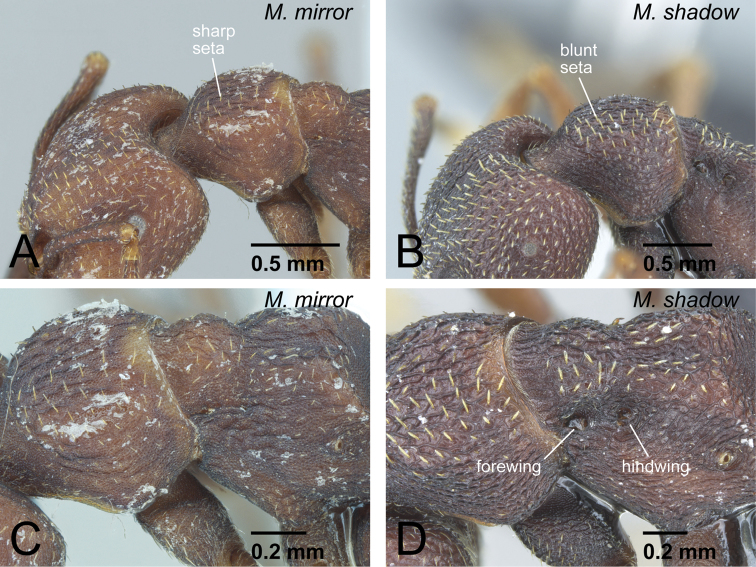
Differences in ergatoid queen characters between *Mystrium mirror* and *Mystrium shadow*. **A, C** *Mystrium mirror* (CASENT0429899) **B, D** *Mystrium shadow* (CASENT0077647: paratype). **A, B** head and pronotum in oblique dorsal view **C, D** mesosoma in oblique dorsal view.

#### Males

Males of *Mystrium labyrinth* and *Mystrium eques* are unknown.

**Table:** 

1	Lateral ocellus relatively large and close to eye; distance between lateral ocellus and eye less than 1.2× maximum diameter of lateral ocellus (Fig. 26A). Eye large, occupying about 0.75× of head length (Fig. 26C). Body relatively brighter	2
–	Lateral ocellus relatively small and distant from eye; distance between lateral ocellus and eye more than 1.2× maximum diameter of lateral ocellus (Fig. 26B). Eye relatively smaller, occupying 0.5-0.6× of head length (Fig. 26D). Body relatively darker	3
2	Distal portion of aedeagus rounded (Fig. 27A). Basal ring gently extending basally (Fig. 27C)	*Mystrium voeltzkowi* Forel
–	Distal portion of aedeagus relatively sharp (Fig. 27B). Basal ring not extending basally (Fig. 27D)	*Mystrium mirror* sp. n.
3	Abdominal tergum VIII punctured (Fig. 28A). Petiolar dorsum covered with rough, deep punctures (Fig. 28C). Eye relatively small, occupying about half of head	4
–	Abdominal tergum VIII smooth and not punctured (Fig. 28B). Petiolar dorsum smooth, or covered with fine punctures, or at most shallow, irregular punctures (Fig. 28D). Eye relatively large, occupying about 0.6× of head length	5
4	Basoventral expansion of aedeagus long, its distal portion relatively narrow and sharp (Fig. 29A)	*Mystrium mysticum* Roger
–	Basoventral expansion of aedeagus short, its distal portion relatively wide and rounded (Fig. 29B)	*Mystrium rogeri* Forel
5	With head in full-face view, dorsal margin of head straight (Fig. 30A)	*Mystrium oberthueri* Forel
–	With head in full-face view, dorsal margin of head somewhat rounded (Fig. 30B)	6
6	Body size smaller (HW<1.20 mm). With head in full-face view, lateral ocelli clearly protruding from dorsal margin of head (Fig. 31A). On forewing, cu-a usually located at junction of Media (M) and Cubitus (Cu), sometimes slightly basal from junction (Fig. 31C). Petiole in dorsal view shorter, its length 0.5× of that of abdominal tergite III	*Mystrium barrybressleri* sp. n.
–	Body size larger (HW>1.50 mm). With head in full-face view, lateral ocelli reaching or failing to reach dorsal margin of head (Fig. 31B). On forewing, cu-a located far basal from junction of Media (M) and Cubitus (Cu) (Fig. 31D). Petiole in dorsal view longer, its length 0.6× or more of that of abdominal tergite III	7
7	Posterior face of vertex gently rising from neck, posterior and dorsal faces almost continuous without division (Fig. 32A). Basoventral expansion of aedeagus relatively short (Fig. 32C)	*Mystrium shadow* sp. n.
–	Posterior face of vertex steeply rising from neck, posterior and dorsal faces clearly divided (Fig. 32B). Basoventral expansion of aedeagus relatively long (Fig. 32D)	*Mystrium janovitzi* sp. n.

**Figure 26.**
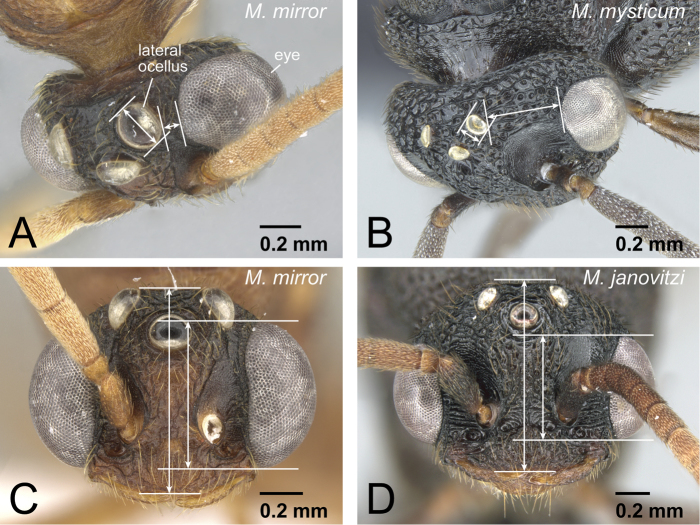
Head of *Mystrium* males. **A, C** *Mystrium mirror* (CASENT0318941) **B** *Mystrium mysticum* (CASENT0429983) **D** *Mystrium janovitzi* (CASENT0007820). **A, B** in oblique dorsal view **C, D** full-face view.

**Figure 27.**
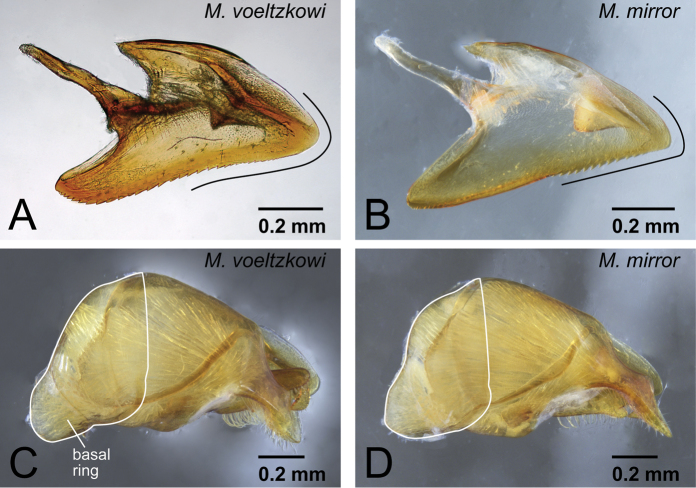
Differences in male characters between *Mystrium voeltzkowi* and *Mystrium mirror*. **A, C** *Mystrium voeltzkowi* (CASENT0317583) **B, D** *Mystrium mirror* (CASENT0317444). **A, B** aedeagus in lateral view **C, D** genital capsule in lateral view.

**Figure 28.**
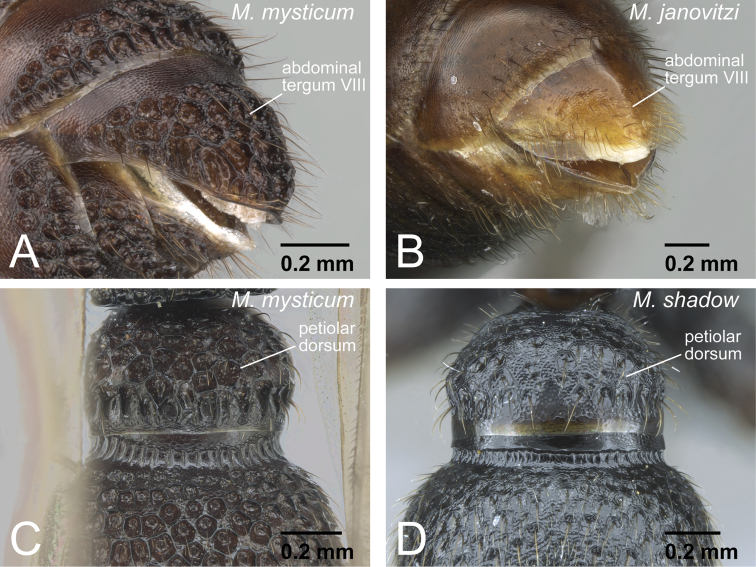
Abdomen of *Mystrium* males. **A** *Mystrium mysticum* (CASENT0318027) **B** *Mystrium janovitzi* (CASENT0007820) **C** *Mystrium mysticum* (CASENT0429983) **D** *Mystrium shadow* (CASENT0303561). **A, B** abdominal segment VIII in oblique lateral view **C, D** petiole in dorsal view.

**Figure 29.**
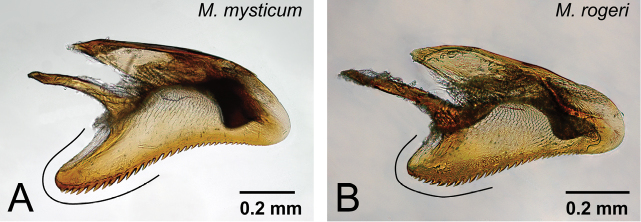
Differences in male genital characters between *Mystrium mysticum* and *Mystrium rogeri*. **A** *Mystrium mysticum* (CASENT0218102) **B** *Mystrium rogeri* (CASENT0001152). **A, B** aedeagus in lateral view.

**Figure 30.**
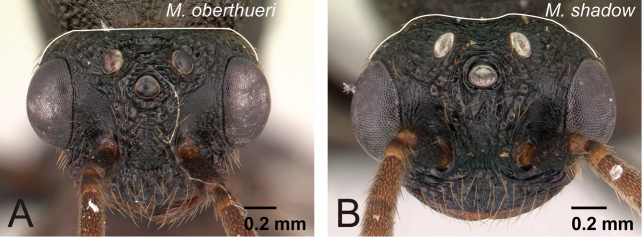
Head of *Mystrium* males in full-face view. **A** *Mystrium oberthueri* (CASENT0179499) **B** *Mystrium shadow* (CASENT0107647).

**Figure 31.**
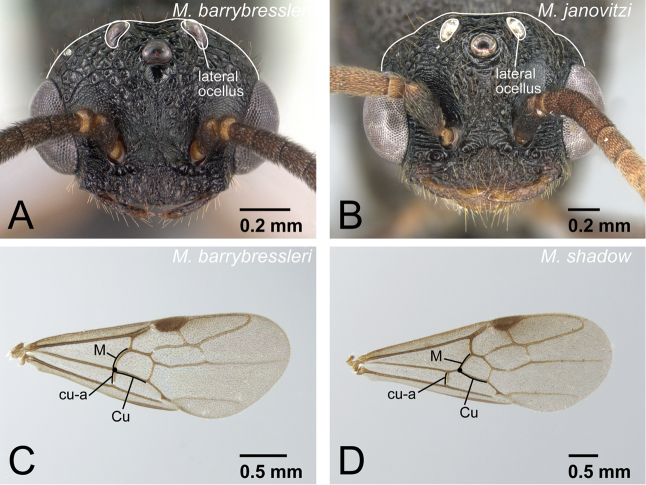
*Mystrium* males. **A** *Mystrium barrybressleri* (CASENT0135866) **B** *Mystrium janovitzi* (CASENT0007820) **C** *Mystrium barrybressleri* (CASENT0078803) **D** *Mystrium shadow* (CASENT0107674). **A, B** head in full-face view **C, D** right forewing in dorsal view.

**Figure 32.**
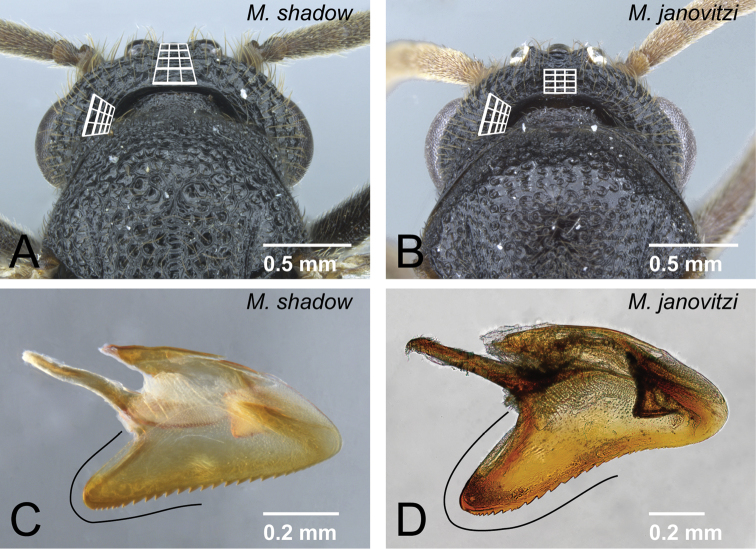
Differences in male characters between *Mystrium shadow* and *Mystrium janovitzi*. **A** *Mystrium shadow* (CASENT0303561) **B** *Mystrium janovitzi* (CASENT0080292) **C** *Mystrium shadow* (CASENT0107647) **D** *Mystrium janovitzi* (CASENT0080644). **A, B** head in posterior view **C, D** aedeagus in lateral view.

### Review of species

Due to the remarkable intra-specific morphological variation in each species, most specific characters are distinct only when individuals belonging to the same caste and in the same body size range are compared. The definition of each caste is given in each species-group description. Characters unique to each species group in the Malagasy region are given in italics.

#### *camillae* species group

The *camillae* species group has two species in the Malagasy region, *Mystrium labyrinth* sp. n. and *Mystrium barrybressleri* sp. n., in addition to the species known from outside of the Malagasy region: *Mystrium camillae* Emery, 1889, *Mystrium leonie* Bihn & Verhaagh, 2007, *Mystrium maren* Bihn & Verhaagh, 2007 from Asia and *Mystrium silvestrii* Santschi, 1914 from Africa. In addition to the four described extralimital species, we recognize two more undescribed morphospecies, one from Indonesia (*Mystrium* id01: CASENT0102169) and one from Mozambique (*Mystrium* mz01: CASENT0318957) in the species group. In this study we include minimal discussion of the species outside the Malagasy region because the material currently available is insufficient for an in-depth analysis. The *camillae* species group is based on the distinctly reticulate head and pronotal dorsum (Fig. 13A). Although several exceptions have been observed outside of the Malagasy region, *the second maxillary palpomere, which is shorter than the third, differentiates this species group from the other* Mystrium *species in the Malagasy region* (Fig. 13C). All known queens have wings, developed eyes and complete ocelli as is usual among ant queens. Rarely, the wings are shrunken and incomplete, as observed in an individual of *Mystrium camillae* collected in Indonesia (CASENT0009854), but the wing sclerites, e.g. the division of mesoscutum, mesoscutellum, and metanotum, are still fully developed (as in Fig. 7A).

Two phenotypes were observed in conspecific workers of *Mystrium barrybressleri*, *Mystrium camillae*, and *Mystrium* mz01 from Mozambique (Table 1): the mandible is stouter and the apical tooth is feebly sclerotized and curved inward in one phenotype (as in Fig. 15A), while the mandible is narrower and the apical tooth is moderately sclerotized and directed ventrally in the other (as in Fig. 15B). The differences between these two worker phenotypes in terms of mandible shape, body size, setae, and body color, are similar to those between workers and ergatoid queens in the *voeltzkowi* species group (see the *voeltzkowi* group description below for detail); however, these differences are relatively less pronounced. The small workers are also lack the wing buds, functional spermatheca, and developed ovaries, which were found in ergatoid queens in the *voeltzkowi* species group (Molet et al. 2009). The function of the two phenotypes in the *camillae* species group has not yet been clarified. All known queens in this group are alate, at least with complete wing sclerites. Hence in the present study, we regard these two phenotypes as a variation of the worker caste. We applied the term major worker to the phenotype with the curved apical tooth, and minor worker to the phenotype with the straight apical tooth.

Although *Mystrium camillae* is not found in the Malagasy region, we designate the lectotype for *Mystrium camillae* and give some remarks below to discuss the diagnostic characters of *Mystrium barrybressleri* more clearly.

##### Mystrium barrybressleri

Yoshimura & Fisher sp. n.

http://zoobank.org/0FFDA298-4426-4C05-88F2-A8712FE01526

http://species-id.net/wiki/Mystrium_barrybressleri

Figs 8B , 10A, 10C , 13A, 13C , 14A , 31A, 31C , 33A–C , 34A–C , 35A–B , 36A, 36C, 36E , 37A , 38A , 39A , 40A , 41A , 42A

###### Holotype.

Worker: CASENT0129838, BLF15450, MADAGASCAR, Toliara, Forêt Ivohibe 55.0 km N Tolagnaro (-24.569°, 47.204°), 200 m alt., 2–4.xii.2006, B.L.Fisher et al. leg. [CASC].

###### Paratypes.

2 workers: CASENT0129839 [BMNH], CASENT0129840 [MHNG], same data as holotype.

###### Worker. 

**Description.** Measurements: holotype. HL 1.26, HW 1.14, SL 0.69, ML 1.08, HD 0.72, WL 1.18, PnW 0.63, PpW 0.50, PtW 0.56, PtL 0.28, CI 90.5, SI 60.7, MI 94.5, PpI 79.7, PtI 198.2.

HL 0.79–1.52, HW 0.71–1.49, SL 0.46–0.89, ML 0.67–1.41, HD 0.47–0.91, WL 0.85–1.51, PnW 0.46–0.78, PpW 0.36–0.60, PtW 0.39–0.68, PtL 0.19–0.30, CI 89.7–100.4, SI 56.0–69.9, MI 86.4–97.5, PpI 77.1–87.5, PtI 199.0–227.8 (10 specimens measured).

Posterolateral corner of head strongly to moderately expanding posteriorly. Posterior face of vertex forming slightly blunt angle with dorsal face on median line of head, so that declivity of vertex on lateral part slightly steeper than on median part. Ventral half of vertex sculptured. Eye small, only with several ommatidia. Anterior margin of clypeus convex with long, conical setae, of which median pair larger than adjacent pair. Genal tooth of head moderately developed, reaching about half of lateral lobe of clypeus. Masticatory margin of mandible slightly visible in full-face view, and dorsal surface on distal portion of mandible slightly wider than that on mandibular shaft. Second maxillary palpomere shorter than third. First flagellomere (third antennal segment) about 0.5× length of pedicel (second antennal segment). Central part of pronotal dorsum strongly to weakly, and regularly reticulate. Lateral surface of pronotum strongly and regularly reticulate on its posterior portion. Mesonotum distinct in dorsal view, its length slightly shorter than that of propodeum. Metanotal groove shallowly impressed and mesonotum higher than pronotum in lateral view. No carina present on dorsal edge of metapleural gland bulla. Petiole extremely wide in dorsal view (PtI>195).

Body color yellowish brown to reddish brown.

###### Queen. 

**Description.** Measurements: HL 1.43, HW 1.52, SL 0.88, ML 1.10, HD 0.99, WL 2.05, MnW 1.23, PtW 1.12, PtL 0.46, CI 106.8, SI 58.0, MI 72.1, MnI 80.8, PtI 243.1 (one specimen measured).

Wings present and well developed. Wing sclerites fully developed even if wings have been removed. Posterolateral corner of head moderately expanding posteriorly; expansion weaker than that of workers. Posterior surface of vertex forming slightly blunt angle with its dorsal surface on median line of head, so that declivity of vertex on lateral part slightly steeper than on median part. Ventral half of vertex weakly sculptured, almost smooth. Eye well developed. Both anterior and lateral ocelli clearly present, median portion of lateral ocelli and posterior portion of anterior ocellus edged by blackish pigment. Anterior margin of clypeus convex with long conical setae, of which median pair larger than adjacent pair. Genal tooth of head moderately developed, reaching about half of lateral lobe of clypeus. Masticatory margin of mandible visible in full-face view, and width of dorsal surface on distal portion slightly wider than that on mandibular shaft. Spoon-shaped seta present on basal side of each basal denticle on masticatory margin of mandible. First flagellar segment on antenna short, about 0.5× length of pedicel. Setae on pronotum almost simple, narrowing distally with strongly sharpened apex. Propodeal declivity in lateral view straight, making right angle with its dorsal margin. Petiole wide and short in dorsal view, 0.25× length of abdominal segment III.

Body color yellow to yellowish brown.

###### Male. 

**Description.** Measurements: HL 0.72–0.86, HW 0.91–1.17, SL 0.16–0.22, EL 0.39–0.45, WL 1.28–1.78, MnW 0.81–1.22, CI 126.8–137.5, SI 16.7–18.9, EI 52.5–55.7, MnI 88.8–108.2 (5 specimens measured).

Eye moderately large, occupying 0.6× head length. Ocelli protruding from dorsal margin of head in full-face view. Dorsal margin of head in full-face view rounded. Both anterior and lateral ocelli small. Lateral ocellus small and distant from eye: distance between these more than 2× maximum diameter of lateral ocellus. Posterior half of vertex clearly differentiated from its dorsal half, its dorsal face distinctly shorter than its posterior face. Palpal formula 4,3. First segment of maxillary palp flattened and distinctly wider than second segment. Second maxillary palpomere shorter than third. Notauli weakly impressed on mesoscutum, but often unclear or absent. Petiole in dorsal view thin, its length 0.5× that of abdominal tergite III. Petiolar dorsum covered with fine punctures. Abdominal tergum VIII without deep punctures, almost smooth.

Distal portion of abdominal sternum IX smooth and not punctured. Basal ring quite short, not extending basally. Telomere slightly extending distally more than digitus. Basoventral expansion of aedeagus well developed basoventrally, distinctly longer than dorsal extension. Ventral margin of aedeagus gently curved ventrally in lateral view. Aedeagus moderately narrowing distally on its distal half and its distal portion narrowly rounded.

On forewing, cu-a usually located at junction of Media (M) and Cubitus (Cu), sometimes slightly basal from junction.

Body color reddish brown to blackish brown.

**Figure 33.**
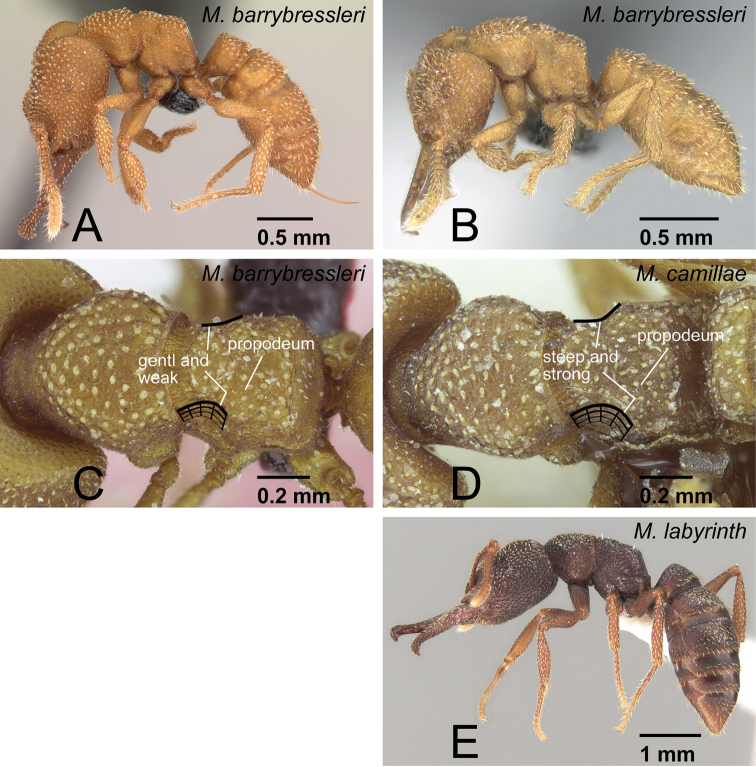
Workers of *camillae* species group. **A, C** *Mystrium barrybressleri* sp. n. (CASENT0129838) **B** *Mystrium barrybressleri* sp. n. (CASENT0129840) **D** *Mystrium camillae* (CASENT0102123) **E** *Mystrium labyrinth* sp. n. (CASENT0003281). **A, C** major worker (holotype) **B** minor worker (paratype) **D** major worker (lectotype) **E** minor worker (holotype). **A, B, E** head to abdomen in lateral view **C, D** mesosoma in oblique dorsal view **C** the lateral portion of the propodeum is gently and weakly convex **D** the lateral portion of the propodeum is steeply and strongly convex.

**Figure 34.**
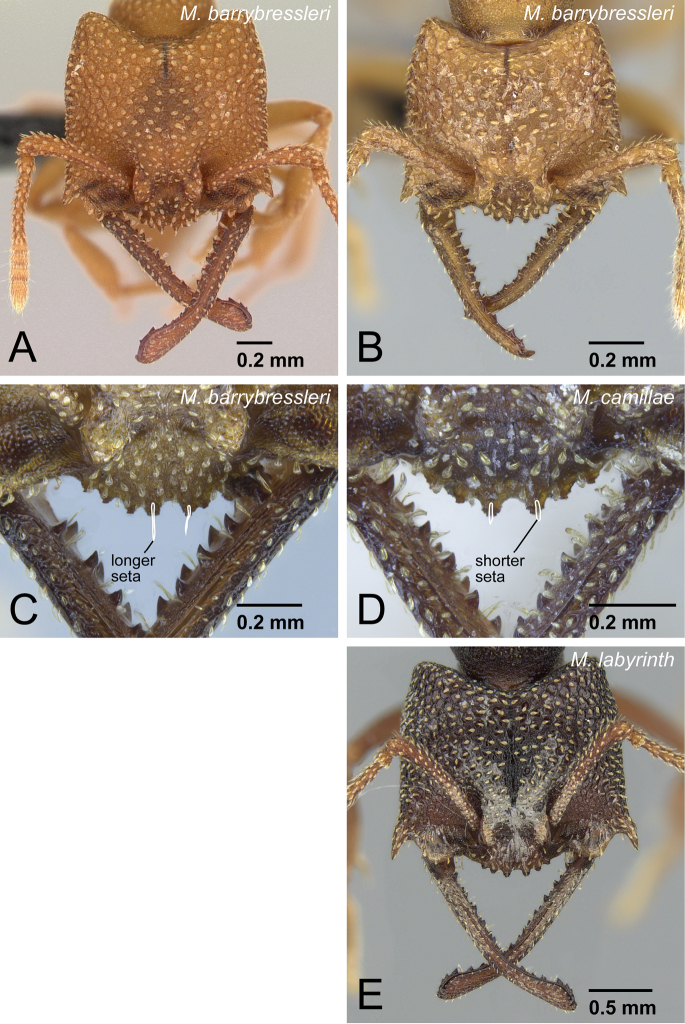
Head of workers of *camillae* species group. **A** *Mystrium barrybressleri* sp. n. (CASENT0129838) **B** *Mystrium barrybressleri* sp. n. (CASENT0129840) **C** *Mystrium barrybressleri* sp. n. (CASENT0045009) **D** *Mystrium camillae* (CASENT0102123) **E** *Mystrium labyrinth* sp. n. (CASENT0003281). **A** major worker (holotype) **B** minor worker (paratype) **C** major worker **D** major worker (lectotype) **E** minor worker (holotype) **A, B, E** in full-face view **C, D** clypeus in oblique anterior view **C** a pair of spatulate setae on the anteromedial portion of clypeus is long **D** a pair of spatulate setae on the anteromedial portion of clypeus is short.

**Figure 35.**
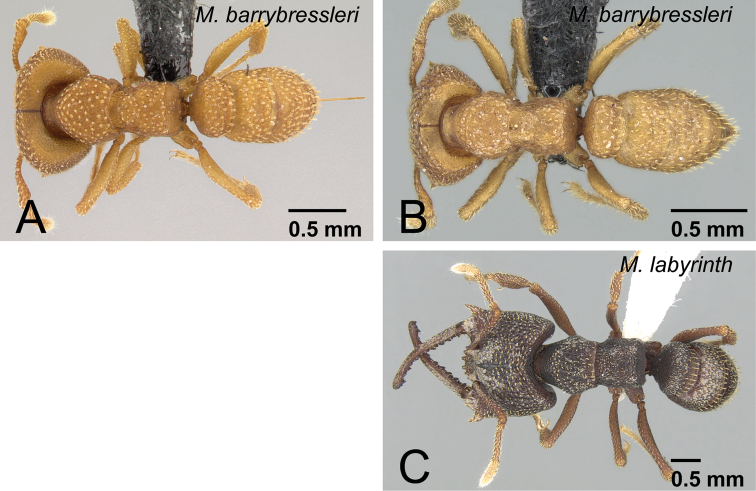
Workers of *camillae* species group in dorsal view. **A** *Mystrium barrybressleri* sp. n. (CASENT0129838) **B** *Mystrium barrybressleri* sp. n. (CASENT0129840) **C** *Mystrium labyrinth* sp. n. (CASENT0003281). **A** major worker (holotype) **B** minor worker (paratype) **C** minor worker (holotype).

**Figure 36.**
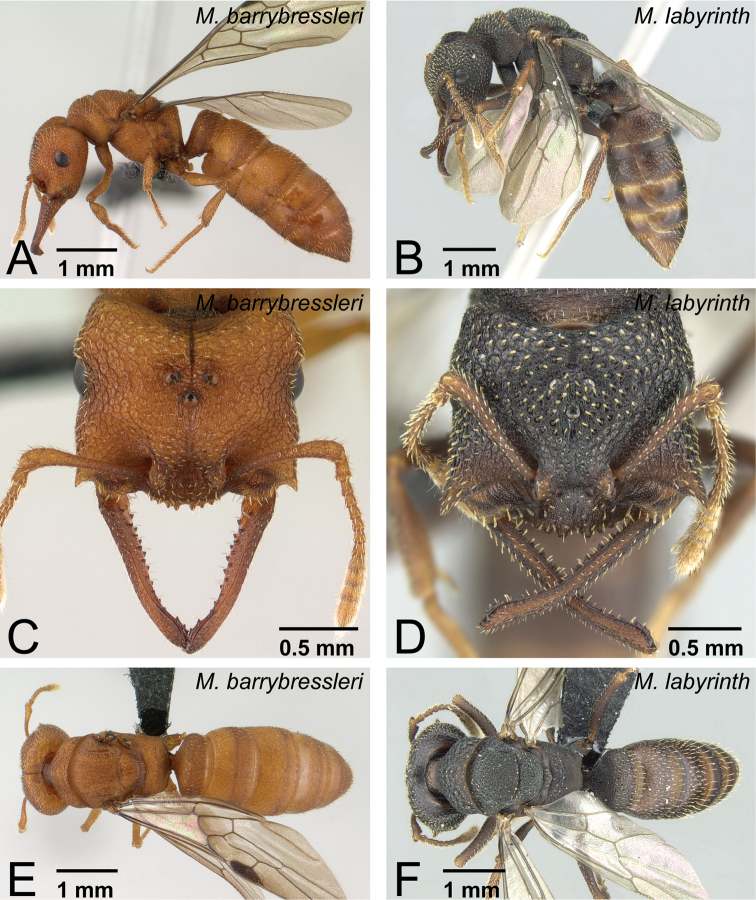
Queens of *camillae* species group. **A, C, E** *Mystrium barrybressleri* sp. n. (CASENT0145109) **B, D, F** *Mystrium labyrinth* sp. n. (CASENT0084950). **A, B** head to abdomen in lateral view **C, D** head in full-face view **E, F** head to abdomen in dorsal view.

**Figure 37.**
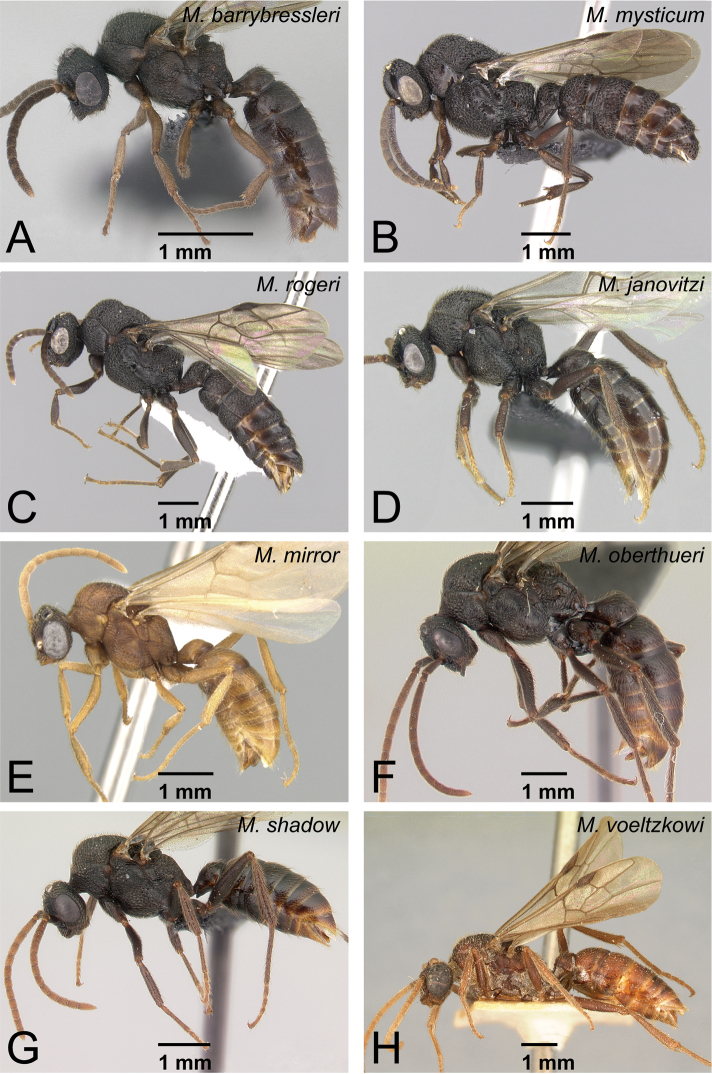
Males of *Mystrium* in lateral view. **A** *Mystrium barrybressleri* sp. n. (CASENT0135866) **B** *Mystrium mysticum* (CASENT0318027) **C** *Mystrium rogeri* (CASENT0001083) **D** *Mystrium janovitzi* sp. n. (CASENT0007820) **E** *Mystrium mirror* sp. n. (CASENT0318941) **F** *Mystrium oberthueri* (CASENT0179499) **G** *Mystrium shadow* sp. n. (CASENT0107647) **H** *Mystrium voeltzkowi* (CASENT0101673).

**Figure 38.**
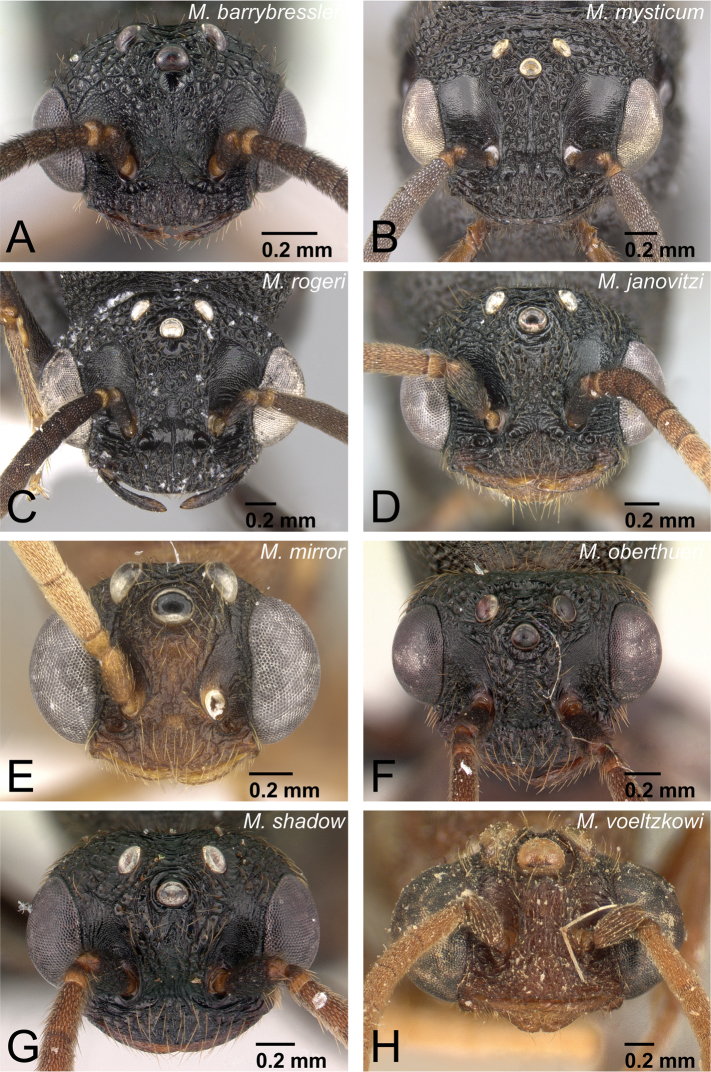
Head of *Mystrium* males in full-face view. **A** *Mystrium barrybressleri* sp. n. (CASENT0135866) **B** *Mystrium mysticum* (CASENT0318027) **C** *Mystrium rogeri* (CASENT0001083) **D** *Mystrium janovitzi* sp. n. (CASENT0007820) **E** *Mystrium mirror* sp. n. (CASENT0318941) **F** *Mystrium oberthueri* (CASENT0179499) **G** *Mystrium shadow* sp. n. (CASENT0107647) **H** *Mystrium voeltzkowi* (CASENT0101673).

**Figure 39.**
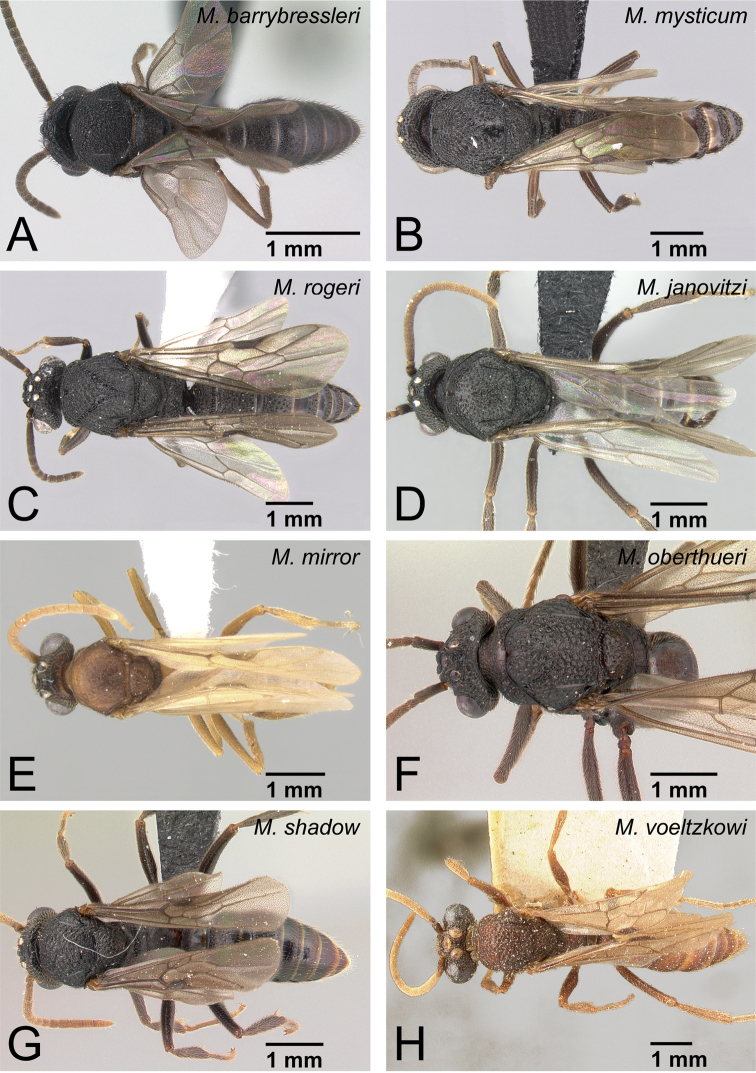
Males of *Mystrium* in dorsal view. **A** *Mystrium barrybressleri* sp. n. (CASENT0135866) **B** *Mystrium mysticum* (CASENT0318027) **C** *Mystrium rogeri* (CASENT0001083) **D** *Mystrium janovitzi* sp. n. (CASENT0007820) **E** *Mystrium mirror* sp. n. (CASENT0318941) **F** *Mystrium oberthueri* (CASENT0179499) **G** *Mystrium shadow* sp. n. (CASENT0107647) **H** *Mystrium voeltzkowi* (CASENT0101673).

**Figure 40.**
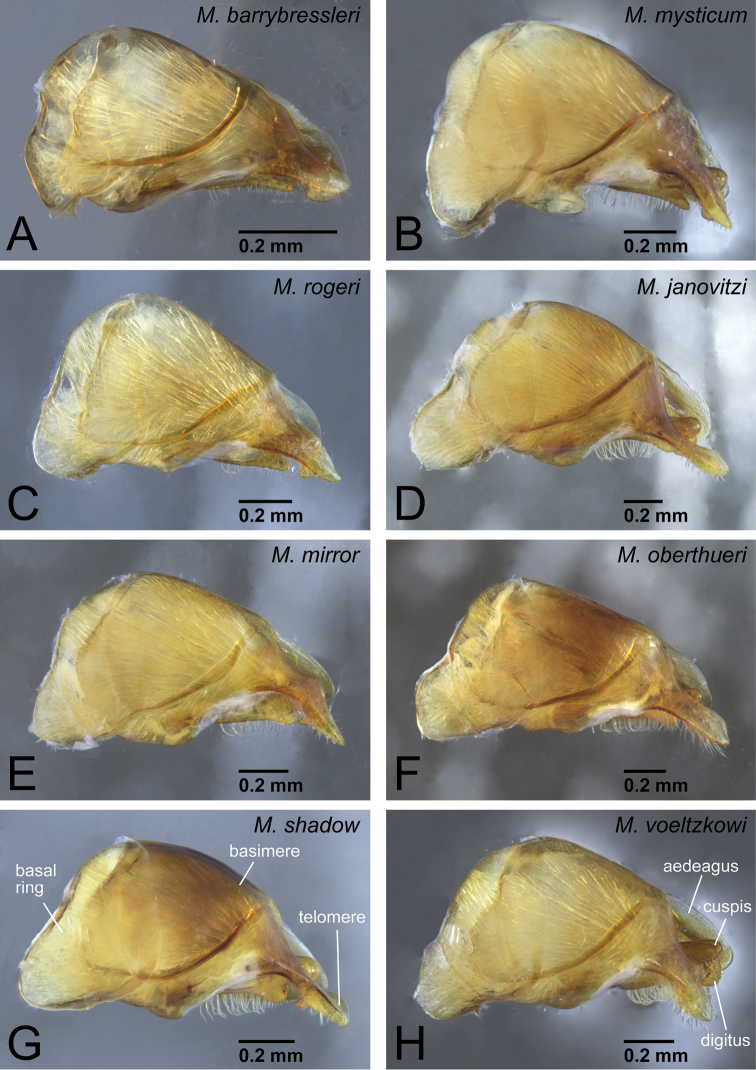
Genital capsule of *Mystrium* males in lateral view. **A** *Mystrium barrybressleri* sp. n. (CASENT0078803) **B** *Mystrium mysticum* (CASENT0218102) **C** *Mystrium rogeri* (CASENT0001152) **D** *Mystrium janovitzi* sp. n. (CASENT0080644) **E** *Mystrium mirror* sp. n. (CASENT0317444) **F** *Mystrium oberthueri* (CASENT0317422) **G** *Mystrium shadow* sp. n. (CASENT0107647) **H** *Mystrium voeltzkowi* (CASENT0317583).

**Figure 41.**
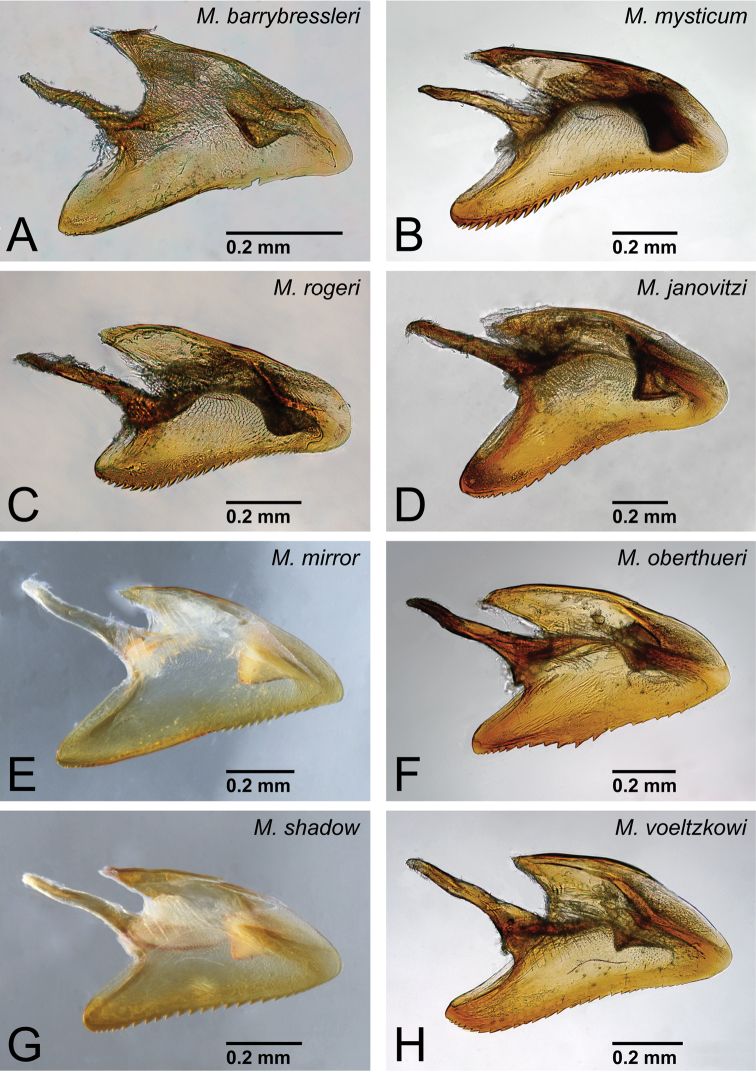
Aedeagus of *Mystrium* males in lateral view. **A** *Mystrium barrybressleri* sp. n. (CASENT0078803) **B** *Mystrium mysticum* (CASENT0218102) **C** *Mystrium rogeri* (CASENT0001152) **D** *Mystrium janovitzi* sp. n. (CASENT0080644) **E** *Mystrium mirror* sp. n. (CASENT0317444) **F** *Mystrium oberthueri* (CASENT0317422) **G** *Mystrium shadow* sp. n. (CASENT0107647) **H** *Mystrium voeltzkowi* (CASENT0317583).

**Figure 42.**
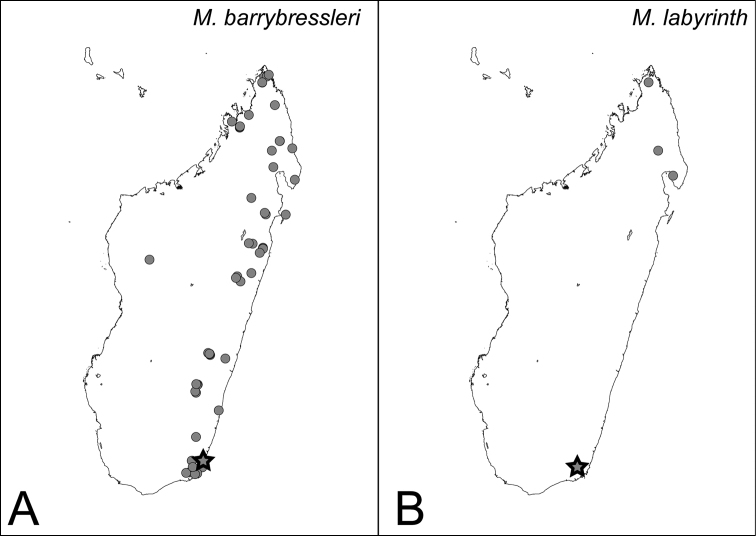
Distribution maps for *camillae* species group. **A** *Mystrium barrybressleri* sp. n. **B** *Mystrium labyrinth* sp. n. Star symbols represent the type locality.

###### Etymology.

The specific epithet is the patronym of Dr. Barry Lee Bressler, retired physicist, former adjunct professor of physics at Virginia Polytechnic Institute and State University, and amateur naturalist, in recognition of his interest in myrmecology and his support for research on ants.

###### Distribution.

MADAGASCAR: as in Figure 42A.

###### Additional material examined.

In addition to the type material, specimens from the following localities were examined in this study: MADAGASCAR. Diego-Suarez. Sakalava Beach [vegetated beach dunes] (-12.26278°, 49.3975°), across sandy trail in dwarf littoral forest, 10 m alt.; Montaigne Francais (-12.325°, 49.33333°), along forested limestone ridge, 150 m alt.; 7 km N Joffreville [camp 2 of Fisher] (-12.33333°, 49.25°), in dry forest, 360 m alt.; Parc National Montagne d’Ambre [1st campsite] (-12.51444°, 49.18139°), rainforest, 960 m alt.; [Petit Lac road] (-12.52028°, 49.17917°), rainforest, 1125 m alt.; Antsiranana. Forêt de Binara, 9.4 km 235° SW Daraina (-13.26333°, 49.6°), montane rainforest, 1100 m alt.; 9.1 km 233° SW Daraina (-13.26333°, 49.60333°), rainforest, 800 m alt.; Ampasindava, Forêt d’Ambilanivy, 3.9 km 181° S Ambaliha (-13.79861°, 48.16167°), rainforest, 600 m alt.; R.S. Manongarivo, 10.8 km 229° SW Antanambao (-13.96167°, 48.43333°), rainforest, 400 m alt.; Manongarivo, 12.8 km 228° SW Antanambao (-13.97667°, 48.42333°), rainforest, 780 m alt.; 14.5 km 220° SW Antanambao (-13.99833°, 48.42833°), montane rainforest, 1175 m alt.; Parc National de Marojejy, Manantenina River, 27.6 km 35° NE Andapa, 9.6 km 327° NNW Manantenina (-14.435°, 49.76°), rainforest, 775 m alt.; 28.0 km 38° NE Andapa, 8.2 km 333° NNW Manantenina (-14.43667°, 49.775°), rainforest, 450 m alt.; SAVA Region, district of Sambava, Marojejy National Park, 5 km W of Manantenina village, 1st campsite (Mantella) (-14.43817°, 49.774°), low altitude rainforest, 487 m alt.; Forêt Ambanitaza, 26.1 km 347° Antalaha (-14.67933°, 50.18367°), rainforest, 240 m alt.; 6.5 km SSW Befingotra, Rés. Anjanaharibe-Sud (-14.75°, 49.5°), rainforest, 875 m alt.; Fotodriana, Cap Masoala (-15.69694°, 50.27028°), lowland rainforest, 25 m alt.; Toamasina. Montagne d’Akirindro 7.6 km 341° NNW Ambinanitelo (-15.28833°, 49.54833°), rainforest, 600 m alt.; Réserve Spéciale Ambatovaky, Sandrangato river (-16.7633°, 49.26692°), rainforest, 520 m alt.; (-16.81753°, 49.29498°), rainforest, 360 m alt.; Ile Sainte Marie, Forêt Ambohidena, 22.8 km 44° Ambodifotatra (-16.82433°, 49.96417°), littoral rainforest, 20 m alt.; Parc National de Zahamena, Tetezambatana forest, near junction of Nosivola and Manakambahiny Rivers (-17.74298°, 48.72936°), rainforest, 860 m alt.; Onibe River (-17.75908°, 48.85468°), rainforest, 780 m alt.; Reserve Betampona, Camp Vohitsivalana, 37.1 km 338° Toamasina (-17.88667°, 49.2025°), rainforest, 520 m alt.; Réserve Naturelle Betampona, 34.1 km 332° Toamasina (-17.916135°, 49.20185°), rainforest, 550 m alt.; 34.08 km 332° Toamasina (-17.91977°, 49.20039°), rainforest, 525 m alt.; Camp Rendrirendry 34.1 km 332° Toamasina (-17.924°, 49.19967°), rainforest, 390 m alt.; F.C. Sandranantitra (-18.04833°, 49.09167°), rainforest, 450 m alt.; F.C. Andriantantely (-18.695°, 48.81333°), rainforest, 530 m alt.; Analamay (-18.80623°, 48.33707°), montane rainforest, 1068 m alt.; Ambatovy, 12.4 km NE Moramanga (-18.84773°, 48.29568°), montane rainforest, 1000 m alt.; Torotorofotsy (-18.87082°, 48.34737°), montane rainforest, marsh edge, 1070 m alt.; 7 km SE Andasibe National Park Headquarters (-18.96278°, 48.45267°), tropical forest, 1050 m alt.; Mahajanga. Réserve Spéciale Marotandrano, Marotandrano 48.3 km S Mandritsara (-16.28322°, 48.81443°), transition humid forest, 865 m alt.; Toliara. Réserve Spéciale d’Ambohijanahary, Forêt d’Ankazotsihitafototra, 35.2 km 312° NW Ambaravaranala (-18.26667°, 45.40667°), montane rainforest, 1050 m alt.; 11 km NW Enakara, Rés. Andohahela (-24.56667°, 46.83333°), rainforest, 800 m alt.; 10 km NW Enakara, Rés. Andohahela (-24.56667°, 46.81667°), rainforest, 430 m alt.; Parc National Andohahela, Col de Tanatana, 33.3 km NW Tolagnaro (-24.7585°, 46.85367°), rainforest, 275 m alt.; 2.7 km WNW 302° Ste. Luce (-24.77167°, 47.17167°), littoral rainforest, 20 m alt.; 29.5 km WNW Tolanaro, Vasiha Mt. (-24.92306°, 46.74083°), rainforest, 700 m alt.; Tsimelahy - Parcel II, Andohahela National Park, transition forest (-24.93683°, 46.62667°), transition forest, 180 m alt.; Mandena, 8.4 km NNE 30° Tolagnaro (-24.95167°, 47.00167°), littoral rainforest, 20 m alt.; Manatantely, 8.9 km NW Tolagnaro (-24.9815°, 46.92567°), rainforest, 100 m alt. Fianarantsoa. Vohiparara broken bridge, Fianarantsoa Prov. (-21.22617°, 47.36983°), high altitude rainforest, 1110 m alt.; 7 km W Ranomafana National Park (-21.25°, 47.41667°), montane rainforest, 900 m alt.; radio tower, Ranomafana National Park, Fianarantsoa Prov. (-21.25083°, 47.40717°), forest edge, mixed tropical forest, open area, 1130 m alt.; Belle Vue trail, Ranomafana National Park, Fianarantsoa Prov. (-21.2665°, 47.42017°), mixed tropical forest, 1020 m alt.; Parc National de Ranomafana, Vatoharanana River, 4.1 km 231° SW Ranomafana (-21.29°, 47.43333°), montane rainforest, 1100 m alt.; 7.6 km 122° Kianjavato, Forêt Classée Vatovavy (-21.4°, 47.94°), rainforest, 175 m alt.; 36 km S Ambalavao, Rés. Andringitra (-22.2°, 46.96667°), montane rainforest, 820 m alt.; 45 km S. Ambalavao (-22.21667°, 47.01667°), rainforest, 785 m alt.; 43 km S Ambalavao, Rés. Andringitra (-22.23333°, 47°), rainforest, 825 m alt.; 9.0 km NE Ivohibe (-22.42667°, 46.93833°), rainforest, 900 m alt.; R.S. Ivohibe, 7.5 km ENE Ivohibe (-22.47°, 46.96°), rainforest, 900 m alt.; Manombo Special Reserve, 32 km SE of Farafangana (-23.02183°, 47.72°), lowland rainforest, 36 m alt.; Parc National Befotaka-Midongy, Papango 27.7 km S Midongy-Sud, Mount Papango (-23.83517°, 46.96367°), rainforest, 940 m alt.

###### Remarks.

The workers of *Mystrium barrybressleri* can be distinguished from other *Mystrium* workers and queens in the Malagasy region by a combination of the following characters: mesosoma lacking wing-sclerites; first flagellomere (third segment of antenna) shorter than pedicel (second segment of antenna) (as in Fig. 10C); and second maxillary palpomere shorter than third (Fig. 13C). The wider petiole in dorsal view (Figs 35A, B) separates *Mystrium barrybressleri* workers from the other *Mystrium* workers in the Malagasy region. A combination of smaller body size, small lateral ocelli distant from the eye (as in Fig. 26B), rounded posterior margin of head with protruding lateral ocelli in full-face view (Fig. 31A), cu-a on the forewing located at junction of Media (M) and Cubitus (Cu) (Fig. 31C), wide and short petiole in dorsal view, and fine punctation on abdominal tergite III separates *Mystrium barrybressleri* males from other known *Mystrium* males in the Malagasy region.

The workers of *Mystrium barrybressleri* are most similar to those of *Mystrium camillae* Emery. *Mystrium camillae* appears to be a complex of several species (see discussion in *Mystrium camillae*) but we did not have sufficient material to fully evaluate the castes and worker variation throughout the range of the species complex. *Mystrium barrybressleri* workers can be distinguished by the shape of the propodeum. In workers, the lateral portion of the propodeum is steeply and strongly convex in *Mystrium* “*camillae*” (Fig. 33D), while the convexity is gentle and weak in *Mystrium barrybressleri* (Fig. 33C). Also, in *Mystrium barrybressleri*, minor workers have setae much narrower and simpler than those of major workers (Fig. 35A: major vs. 35B: minor), while in *Mystrium* “*camillae*” minor and major workers have similar setae.

There are additional differences with *Mystrium camillae* (sensu stricto, as defined by the syntype series from Myanmar). The pair of spatulate setae on the anteromedial portion of clypeus is distinctly longer in *Mystrium barrybressleri* (Fig. 34C) than those in the syntype series of *Mystrium camillae* (Fig. 34D). The queen of *Mystrium barrybressleri* can be distinguished by the presence of simple setae on the pronotum, while in *Mystrium camillae* sensu stricto, the setae are clavate. Additional diagnostic characters can be presented once the species limits are further refined for *Mystrium camillae* and related species.

*Mystrium barrybressleri* is found from the rainforests and montane rainforests, and the distribution pattern of this species is similar to that of a forest species *Tetramorium andrei* Forel (Hita Garcia and Fisher 2012: fig. 141). No remarkable difference was observed between specimens from NW Ambaravaranala (-18.26667°, 45.40667°) and those from the other localities, even the former forest is currently isolated from the others.

##### Mystrium camillae

Emery, 1889

http://species-id.net/wiki/Mystrium_camillae

Figs 33D , 34D

Mystrium camillae Emery, 1889. MYANMAR, Bhamo. Syntype: workers and queen [The lectotype is designated below].

###### Lectotype of *Mystrium camillae*

**[here designated].** Worker: CASENT0102123, MYANMAR, Bhamo, iv.1886, Fea leg. [MSNG: examined].

###### Remarks.

The lectotype for *Mystrium camillae* is designated here. We confirm that five workers collected from vi.1885 to iv.1886 are part of the syntype series: two workers in MSNG [CASENT0102123, CASENT0102124]; one worker in MNHN [CASENT0101450]; and two workers in MHNG [CASENT0101809, CASENT0101784]. Although Emery (1889) described a queen in his original description, we could not find the syntype queen. According to the original description (Emery 1889), the queen is dealate, and was not collected from the same colony as any of the syntype workers.

According to the revision of *Mystrium* in the Indo-Australian region (Bihn and Verhaagh 2007), *Mystrium camillae* is widely distributed in the Indomalaya, and Australian regions: from Australia to Brunei, China, India, Indonesia, Malaysia, Myanmar, Papua New Guinea, the Philippines and Singapore. We find that specimens currently determined as *Mystrium camillae* display remarkable morphological variation, some of which appears not to be intra-specific but rather due to differences among species. For example, we found a small-sized queen with vestigial wings in Indonesian material (CASENT0009854), workers with longer setae on the anteromedial portion of the clypeus in specimens from New Guinea, a large queen with simple setae on the pronotal dorsum in specimens from China (CASENT0275389), and a strange yellow male from Australia (CASENT0172083).

We have found that clarifying species boundaries in the genus *Mystrium* is much more complicated than would be expected from previous studies. The differences among some sibling species similar to *Mystrium camillae* could be recognizable only in a particular sex, caste, or phenotype, as in the case of *Mystrium camillae* and *Mystrium barrybressleri*. A reexamination of the species boundaries of *Mystrium camillae* based on a detailed comparative study using comprehensive colony samples from each local region will be necessary.

##### Mystrium labyrinth

Yoshimura & Fisher sp. n.

http://zoobank.org/0E7BCDE0-AF81-4120-9FC0-C1287C4BED46

http://species-id.net/wiki/Mystrium_labyrinth

Figs 10B, 10D , 14B , 33E , 34E , 35C , 36B, 36D, 36F , 42B

###### Holotype.

Worker: CASENT0003281, BLF00976, MADAGASCAR, Toliara, 5 km N Isaka-Invondro (-24.75°, 46.8°), 350 m alt., 12.xi.1992, B.L.Fisher leg. [CASC].

###### Worker. 

**Description.** Measurements: holotype. HL 1.73, HW 1.76, SL 1.08, ML 1.77, HD 1.03, WL 1.96, PnW 1.01, PpW 0.84, PtW 0.90, PtL 0.51, CI 101.7, SI 61.2, MI 100.5, PpI 83.0, PtI 176.1.

HL 1.67–1.79, HW 1.72–1.84, SL 1.05–1.09, ML 1.70–1.76, HD 1.03–1.09, WL 1.90–2.02, PnW 1.02–1.08, PpW 0.81–0.87, PtW 0.82–0.95, PtL 0.45–0.52, CI 102.0–102.8, SI 58.0–61.1, MI 92.2–99.4, PpI 79.0–82.7, PtI 180.4–183.8 (3 specimens measured).

Posterolateral corner of head moderately expanding posteriorly. Posterior face of vertex forming almost right angle with its dorsal face on median line of head, so that declivity of vertex on lateral part as steep as that on median part. Ventral half of vertex sculptured. Eye relatively larger than that of *Mystrium barrybressleri*. Anterior margin of clypeus convex with long conical setae, of which median pair larger than adjacent pair. Genal tooth of head relatively long, as long as lateral lobe of clypeus. Masticatory margin of mandible in full-face view slightly visible on its basal half, invisible on its distal half. Width of dorsal surface of mandible almost identical from mandibular shaft to distal portion. Second maxillary palpomere shorter than third. First flagellomere (third antennal segment) as long as pedicel (second antennal segment). Central part of pronotal dorsum and lateral surface of pronotum strongly and regularly reticulate. Mesonotum often not differentiated and indistinct from propodeum in dorsal view, its length as long as that of propodeum. Metanotal groove indistinct in lateral view, but mesonotum slightly higher than pronotum. Short, but distinct ridge present on dorsal edge of metapleural gland bulla. Petiole wide in dorsal view, but narrower than that of *Mystrium barrybressleri* (PtI<185).

Body color reddish brown to black.

###### Queen. 

**Description.** Measurements: HL 1.65, HW 1.69, SL 0.99, ML 1.48, HD 1.10, WL 2.33, MnW 1.27, PtW 1.08, PtL 0.51, CI 102.5, SI 58.5, MI 87.6, MnI 74.8, PtI 212.2 (one specimens measured).

Wings present, well developed. Wing sclerites fully developed even if wings have dropped off. Posterolateral corner of head moderately expanding posteriorly; expansion weaker than that of workers. Posterior face of vertex forming almost right angle with its dorsal face on median line of head, so that declivity of vertex on lateral part as steep as that on median part. Ventral half of vertex sculptured. Eye well developed. Both anterior and lateral ocelli clearly present, median portion of lateral ocelli and posterior portion of anterior ocellus edged by blackish pigment. Anterior margin of clypeus convex, with long conical setae, of which median pair larger than adjacent pair. Genal tooth of head distinctly developed, reaching slightly posterior of lateral lobe of clypeus. Masticatory margin of mandible almost invisible in full-face view, and dorsal surface on distal portion as wide as that on mandibular shaft. Spatulate seta present on basal side of each basal denticle on masticatory margin of mandible. First flagellar segment on antenna as long as pedicel. Setae on pronotum distinctly spatulate, widened distally. Propodeal declivity in lateral view slightly convex on dorsal part of metanotal gland bulla, making blunt angle with its dorsal margin. Petiole relatively long in dorsal view, 0.5× length of abdominal segment III.

Body color blackish brown.

Male unknown.

###### Etymology.

This species name is the English word labyrinth, inspired by the strong reticulation covering the body surface of the new species. The species epithet is a noun, and thus invariant.

###### Distribution.

MADAGASCAR: as in Figure 42B.

###### Additional material examined.

In addition to the type material, specimens from the following localities were examined in this study: MADAGASCAR. Diego-Suarez. Parc National Montagne d’Ambre (-12.51444°, 49.18139°), rainforest, 960 m alt.; Antsiranana. 6.5 km SSW Befingotra, Rés. Anjanaharibe-Sud (-14.75°, 49.5°), rainforest, 875 m alt.; Toamasina. 6.9 km NE Ambanizana, Ambohitsitondroina (-15.56667°, 50°), rainforest, 825 m alt.; Toliara. 5 km N Isaka-Invondro (-24.75°, 46.8°), rainforest, 350 m alt.

###### Remarks.

*Mystrium labyrinth* females can be distinguished easily from the other *Mystrium* females in the Malagasy region by a combination of the following characters: the pronotal dorsum covered with strong regular reticulation (as in Fig. 13A); the second maxillary palpomere shorter than the third (as in Fig. 13C); the long genal tooth of head nearly reaching anterior end of the lateral lobe of the clypeus (Fig. 14B); and the first flagellomere (third antennal segment) as long as the pedicel (second antennal segment) (Fig. 10D). The worker of *Mystrium labyrinth* is similar to that of *Mystrium silvestrii*; however, *Mystrium labyrinth* can be distinguished from *Mystrium silvestrii* by an anterior clypeal margin with long, well-developed conical setae (weak in *Mystrium silvestrii* and *Mystrium* mz01), and a moderately long petiole (Fig. 35C) (short and wide in *Mystrium silvestrii* as in *Mystrium barrybressleri*).

The queen described here is not associated with workers from the same colony (collected alone from -12.51444°, 49.18139°); however, the similarities between workers and queens are sufficient to determine their relationship. The male of this species is unknown.

#### *mysticum* species group

The *mysticum* species group is endemic to the Malagasy region, and consists of two species, *Mystrium mysticum* Roger, 1862 and *Mystrium rogeri* Forel, 1899. Unique characters in this group are: *a longitudinal carina on the central portion of the labrum in worker and queen* (Fig. 8A); *a single lateral spine on abdominal sternum VII in worker and queen* (Fig. 8C); *and unsculptured ventral area on the vertex in worker* (Fig. 11A). The separation of the queens is usually straightforward in this species group except for intercastes, because queens have wings and completely developed wing-sclerites. The intercastes, which are the intermediate individuals between worker and queen having the lateral and mid ocelli and the wing sclerites, are sometimes found and all characters of this caste could be varied from absent to incompletely developed in variable degrees. These intercastes could be keyed out as either workers or queens; however, the description of the intercastes was included in the queen section.

The two phenotypes mentioned for the *camillae* species group are present in the workers of *mysticum* species group, similarly to the *voeltzkowi* species group (Table 1). One has a somewhat curved apical tooth (as in Fig. 15A), larger body size, and darker body color; and the other has a straight apical tooth, smaller body size, somewhat narrower body setae, and brighter body color. However, the division of the two phenotypes is weaker than that in the *camillae* species group, and no other distinct differences could be found between the two phenotypes except for the four characters mentioned above. The “small bright color” phenotype does not seem to be functional in reproduction as in the ergatoid queens of the *voeltzkowi* species group. In this study, we regard these two phenotypes as morphological variation observed in the worker caste, and we reference the larger one as a major worker and the smaller one as a minor worker when necessary.

##### Mystrium mysticum

Roger, 1862

http://species-id.net/wiki/Mystrium_mysticum

Figs 4C , 7 , 8C , 9A, 9C , 11A , 12A , 26B , 28A, 28C , 29A , 37B , 38B , 39B , 40B , 41B , 43A, 43C , 44A, 44C , 45A, 45C , 46A, 46C , 47A, 47C , 48A, 48C , 49A

Mystrium mysticum Roger, 1862. MADAGASCAR. Syntypes: two queens [lost: the neotype is designated below].
Mystrium mysticum : Forel 1892, in part (queen); Menozzi 1929.
Mystrium mysticum (Not) (male): Forel 1891; Menozzi 1929 [assumed from descriptions].
Mystrium stadelmanni Forel, 1895. **syn. n.**
Mystrium stadelmanni : Forel 1895, in part; Menozzi 1929.

###### Neotype

**[here designated].** Alate queen: CASENT0429914, BLF4770, MADAGASCAR, Toliara, Parc National de Kirindy Mite, 16.3 km 127° SE Belo sur Mer (-20.79528°, 44.147°), 80 m alt., 6-10.xii.2001, Fisher-Griswold Arthropod Team leg. [CASC].

###### Lectotype of *Mystrium stadelmanni*

**[here designated].** Worker: CASENT0104561, MADAGASCAR, Est de I’Imeriua, Sikora leg. [ZMHB: examined].

###### Worker.

**Description.** Measurements: lectotype of *Mystrium stadelmanni*. HL 2.05, HW 1.93, SL 1.16, ML 2.09, HD 1.26, WL 1.99, PnW 0.94, PpW 0.84, PtW 0.80, PtL 0.53, CI 94.3, SI 59.9, MI 108.2, PpI 89.5, PtI 153.0.

HL 1.44–2.27, HW 1.42–2.18, SL 0.86–1.35, ML 1.32–2.31, HD 0.98–1.39, WL 1.63–2.44, PnW 0.77–1.2, PpW 0.69–1.09, PtW 0.6–0.94, PtL 0.4–0.6, CI 96.0–102.0, SI 56–64, MI 91–108, PpI 86–95, PtI 146–168 (10 specimens measured).

Posterolateral corner of head strongly expanding posteriorly. Posterior face of vertex forming slightly blunt angle with its dorsal face on median line of head, so that declivity of vertex on lateral part slightly steeper than on median part. Ventral half of vertex smooth and not sculptured. Eye small to moderately small. Anterior margin of clypeus convex and with short conical setae. Genal tooth of head undeveloped: anterolateral corner of head angulate. Masticatory margin of mandible widely visible in full-face view, difference in width of dorsal surface of mandible relatively large between mandibular shaft and distal portion. Second maxillary palpomere longer than third. First flagellomere (third antennal segment) as long as pedicel (second antennal segment). Shallow and fine longitudinal striae irregularly impressed on central part of pronotal dorsum, sometimes with shallow reticulation around them. On lateral surface of pronotum, shallow, fine longitudinal striae impressed, wide and shallow punctures usually arranged on central horizontal line. Mesonotum differentiated from propodeum in dorsal view, length as long as that of propodeum in large individuals, shorter than propodeum in small individuals. Metanotal groove shallowly impressed in lateral view, mesonotum as high as pronotum in large individuals, higher than pronotum in small individuals. Metanotum weakly developed in largest individuals. Metapleural gland bulla developed, so that propodeal declivity in lateral view weakly convex posteriorly on its ventral portion. Petiole in dorsal view moderately wide, and relatively wider than that of *Mystrium rogeri*.

Body color brown to black.

###### Queen. 

**Description.** Measurements: neotype. HL 2.44, HW 2.51, SL 1.49, ML 2.24, HD 1.54, WL 3.25, MnW 1.73, PtW 1.11, PtL 0.66, CI 102.7, SI 59.3, MI 89.2, MnI 69.1, PtI 168.2.

HL 2.14–2.73, HW 2.08–2.84, SL 1.26–1.60, ML 1.85–2.56, HD 1.32–1.72, WL 2.60–3.48, MnW 1.27–1.92, PtW 0.89–1.33, PtL 0.55–0.77, CI 97.2–104.2, SI 56.3–60.7, MI 87.2–98.2, MnI 61.0–71.3, PtI 160.2–188.8 (10 specimens measured).

Wings usually present and well developed; lacking in intercaste. Wing sclerites fully developed even if wings have dropped off in queen; developed to undeveloped in variable degrees in intercaste. Posterolateral corner of head strongly expanding posteriorly; expansion even stronger than that of workers. Posterior face of vertex forming slightly blunt angle with dorsal face on median line of head, so that declivity of vertex on lateral part slightly steeper than on median part. Ventral half of vertex smooth and not sculptured. Eye well developed. Both anterior and lateral ocelli clearly present and developed in most cases, lateral ones rarely smaller; ocelli varied from absent to developed in intercaste. Anterior margin of clypeus convex with small conical setae. Anterolateral portion of head angulate or with short spine. Masticatory margin of mandible widely visible in full-face view, and dorsal surface on distal portion distinctly wider that on mandibular shaft, difference in width much larger than that in *Mystrium rogeri*. Mandibular teeth (a row of dentition on dorsal side) often lacking on mid-portion of mandibular shaft. A spatulate seta present on basal side of each basal denticle on masticatory margin of mandible. First flagellar segment on antenna as long as pedicel. Setae on pronotum almost simple, narrowing distally with strongly sharpened apex. Propodeal declivity in lateral view almost straight and forming right to slight obtuse angle with dorsal margin, ventral portion with small convexity by metapleural gland. Petiole relatively long in dorsal view, about 0.6× length of abdominal segment III.

Body color black.

###### Male.

**Description.** Measurements: HL 1.13–1.33, HW 1.52–1.81, SL 0.22–0.28, EL 0.62–0.68, WL 2.26–2.86, MnW 1.46–1.89, CI 129.5–136.0, SI 14.3–17.0, EI 49.0–54.8, MnI 96.3–104.4 (7 specimens measured).

Eye relatively small, occupying about 0.5× length of head. Ocelli relatively distant from dorsal margin of head or just failing to reach dorsal margin in full-face view. Dorsal margin of head in full-face view rounded. Both anterior and lateral ocelli small. Distance between lateral ocellus and eye relatively long, about 3× longer than diameter of lateral ocellus. Posterior half of vertex clearly differentiated from dorsal half, its dorsal face almost as long as its posterior face. Palpal formula 4,3. First segment of maxillary palp flattened and distinctly wider than second segment. Second maxillary palpomere longer than third. Notauli clearly impressed on mesoscutum. Petiole in dorsal view thin, its length about 0.65× that of abdominal tergite III. Petiolar dorsum covered with rough, deep punctures. Abdominal tergum VIII roughly and deeply punctured.

Abdominal sternum IX punctured on its distal portion. Basal ring short, not extending basally. Telomere extending slightly further distally than digitus. Basoventral expansion of aedeagus well developed basoventrally, distinctly longer than dorsal extension. Ventral margin of aedeagus strongly curved ventrally in lateral view. Aedeagus weakly narrowing distally, its distal portion widely rounded.

On forewing, cu-a located at junction of Media (M) and Cubitus (Cu).

Body color reddish brown to black.

**Figure 43.**
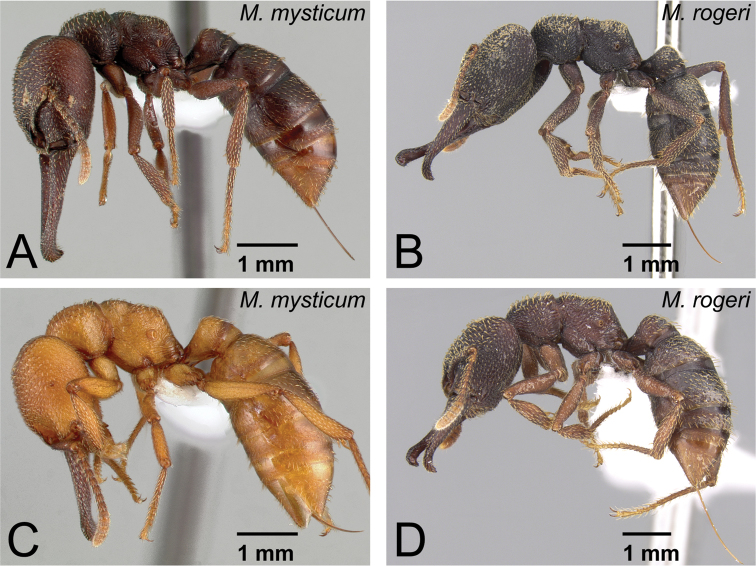
Workers of *mysticum* species group in lateral view. **A, C** *Mystrium mysticum* **B, C** *Mystrium rogeri*. **A** major worker (CASENT0429955) **B** major worker (CASENT0001069) **C** minor worker (CASENT0429953) **D** minor worker (CASENT0001154).

**Figure 44.**
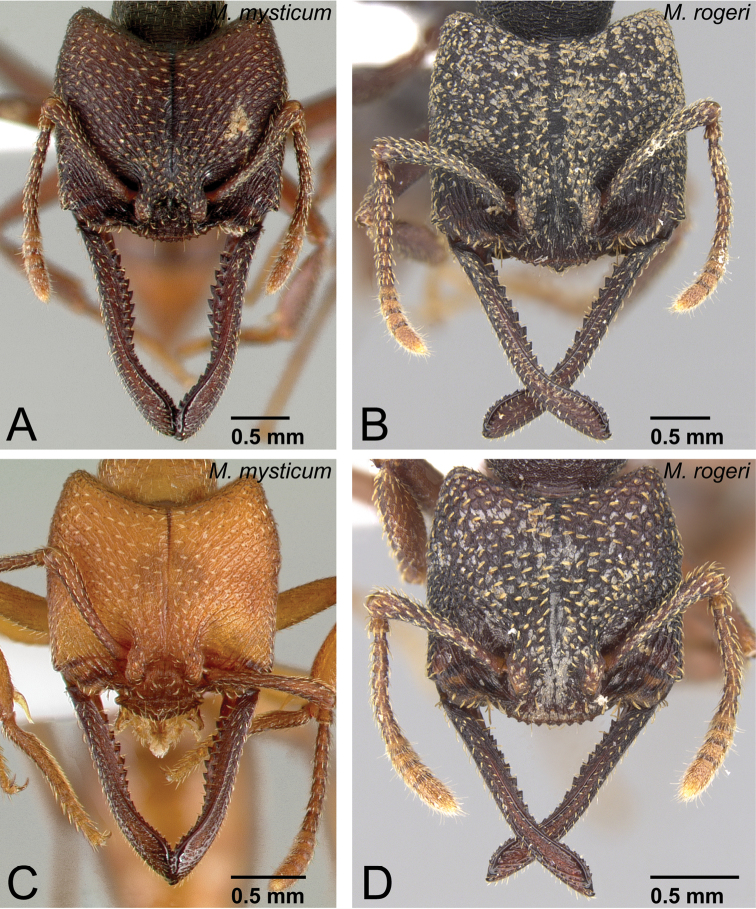
Head of the workers of *mysticum* species group in full-face view. **A, C** *Mystrium mysticum* **B, D** *Mystrium rogeri*. **A** major worker (CASENT0429955) **B** major worker (CASENT0001069) **C** minor worker (CASENT0429953) **D** minor worker (CASENT0001154).

**Figure 45.**
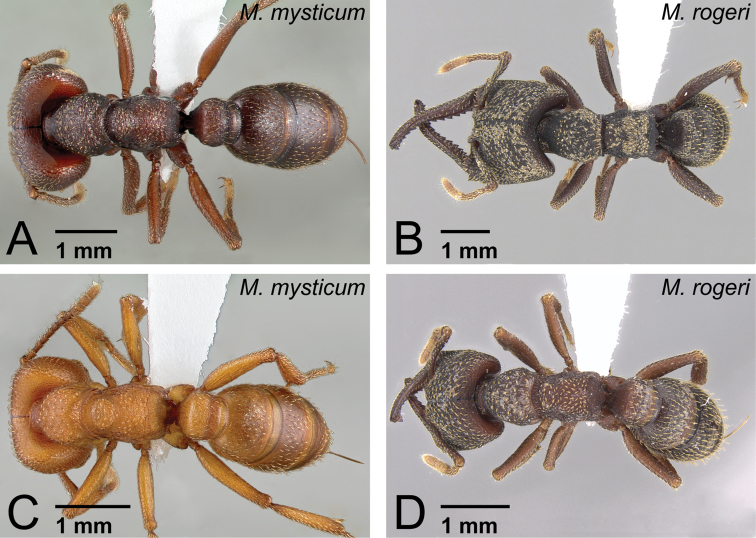
Workers of *mysticum* species group in dorsal view. **A, C** *Mystrium mysticum* **B, D** *Mystrium rogeri*. **A** major worker (CASENT0429955) **B** major worker (CASENT0001069) **C** minor worker (CASENT0429953) **D** minor worker (CASENT0001154).

**Figure 46.**
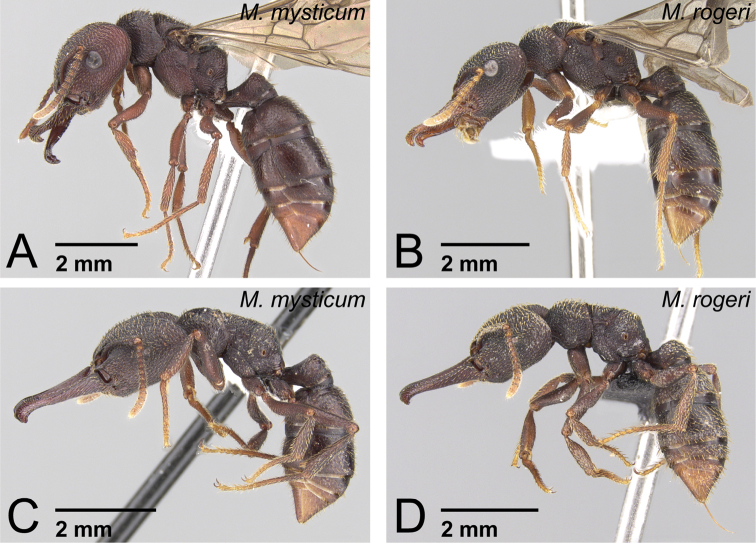
Queens and intercastes of *mysticum* species group in lateral view. **A, C** *Mystrium mysticum* **B, D** *Mystrium rogeri*. **A** alate queen (CASENT0429962) **B** alate queen (CASENT0001079) **C** intercaste (CASENT0429959) **D** intercaste (CASENT0497221).

**Figure 47.**
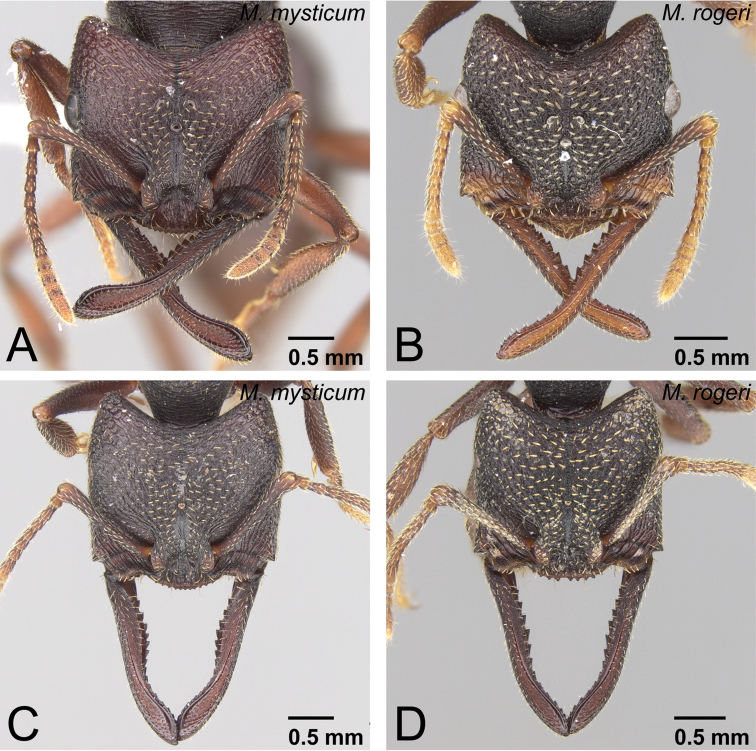
Head of the queens and intercastes of *mysticum* species group in full-face view. **A, C** *Mystrium mysticum* **B, D** *Mystrium rogeri*. **A** alate queen (CASENT0429962) **B** alate queen (CASENT0001079) **C** intercaste (CASENT0429959) **D** intercaste (CASENT0497221).

**Figure 48.**
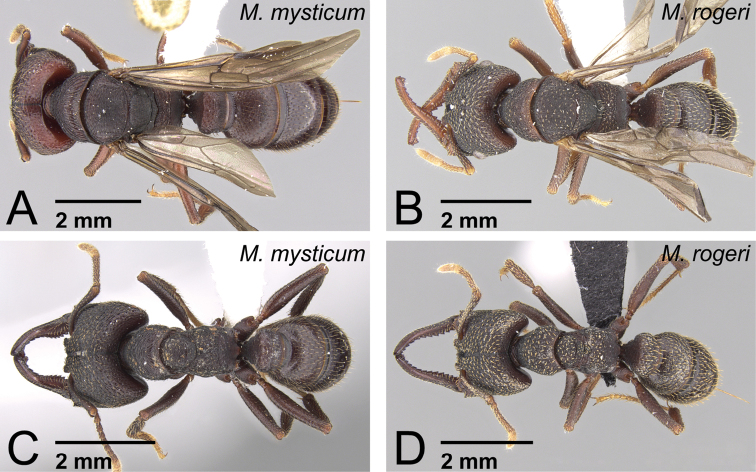
Queens and intercastes of *mysticum* species group in dorsal view. **A, C** *Mystrium mysticum* **B, D** *Mystrium rogeri*. **A** alate queen (CASENT0429962) **B** alate queen (CASENT0001079) **C** intercaste (CASENT0429959) **D** intercaste (CASENT0497221).

**Figure 49.**
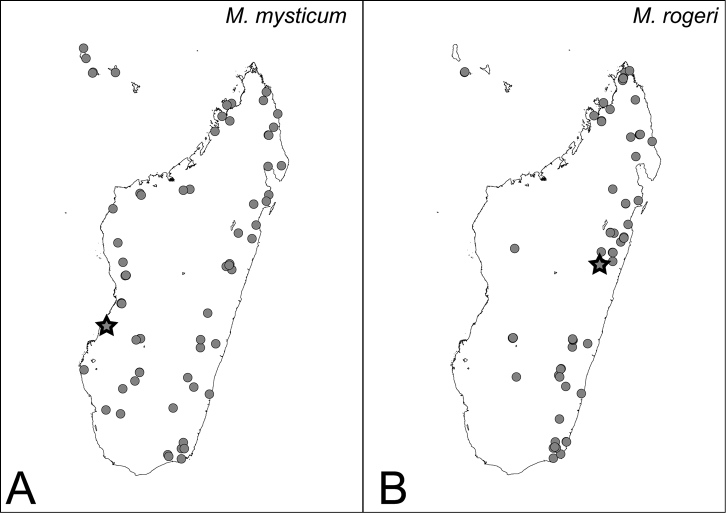
Distribution maps for *mysticum* species group. **A** *Mystrium mysticum* **B** *Mystrium rogeri*. Star symbols represent the type locality: the locality for *Mystrium mysticum* is that of the neotype, and for *Mystrium rogeri* is approximate, which is assumed from the original description.

###### Distribution.

MADAGASCAR and COMOROS: as in Figure 49A.

###### Additional material examined.

In addition to the type material, specimens from the following localities were examined in this study: MADAGASCAR. Antsiranana. Forêt d’Ampondrabe, 26.3 km 10° NNE Daraina (-12.97°, 49.7°), tropical dry forest, 175 m alt.; Forêt de Binara, 9.1 km 233° SW Daraina (-13.26333°, 49.60333°), rainforest, 650-800 m alt.; 9.4 km 235° SW Daraina (-13.26333°, 49.6°), montane rainforest, 1100 m alt.; Nosy Faly (-13.3624°, 48.49101°), open secondary vegetation, 15 m alt.; (-13.36435°, 48.49137°), open secondary vegetation, 40 m alt.; Nosy Be, Réserve Naturelle Intégrale de Lokobe, 6.3 km 112° ESE Hellville (-13.41933°, 48.33117°), rainforest, 30 m alt.; Ambondrobe, 41.1 km 175° Vohemar (-13.71533°, 50.10167°), littoral rainforest, 10 m alt.; Ampasindava, Forêt d’Ambilanivy, 3.9 km 181° S Ambaliha (-13.79861°, 48.16167°), rainforest, 600 m alt. R.S. Manongarivo, 10.8 km 229° SW Antanambao (-13.96167°, 48.43333°), rainforest, 400 m alt.; Makirovana forest (-14.17066°, 49.95409°), rainforest, 225 m alt.; Forêt d’Anabohazo, 21.6 km 247° WSW Maromandia (-14.30889°, 47.91433°), tropical dry forest, 120 m alt.; Parc National de Marojejy, Manantenina River, 28.0 km 38° NE Andapa, 8.2 km 333° NNW Manantenina (-14.43667°, 49.775°), rainforest, 450 m alt.; Marojejy R.N.I. #12 (-14.44533°, 49.78564°), rainforest, 375 m alt.; R. Onive (-15.47°, 50.225°), 150 m alt. Toamasina. Nosy Mangabe, 7.43 km S Maroantsetra (-15.4973°, 49.76223°), littoral rainforest, 3 m alt.; Parc National Mananara-Nord, 7.1 km 261° Antanambe (-16.455°, 49.7875°), rainforest, 225 m alt.; Res. Ambodiriana, 4.8 km 306°Manompana, along Manompana river (-16.67233°, 49.70117°), rainforest, 125 m alt.; Manakambahiny, near Vavatenina Forest (-17.46667°, 49.35°); Parc National de Zahamena (-17.73359°, 48.72625°), rainforest, 950 m alt.; Réserve Nationale Intégrale Betampona, Betampona 35.1 km NW Toamasina (-17.91801°, 49.20074°), rainforest, 500 m alt.; Corridor Forestier Analamay-Mantadia, Tsaravoniana (-18.75737°, 48.42302°), rainforest, 1018 m alt.; Tsaravoniana (-18.76124°, 48.42134°), rainforest, 939 m alt.; Ambatoharanana (-18.8024°, 48.40612°), rainforest, 950 m alt.; (-18.80388°, 48.40506°), rainforest, 1013 m alt.; Bevolota 17.1 km N Andasibe (-18.77071°, 48.43164°), montane rainforest, 995 m alt.; Ambatovy, 12.4 km NE Moramanga (-18.83937°, 48.30842°), montane rainforest, 1080 m alt.; Perinet (-18.95°, 48.5°), 800 m alt.; Mahajanga. Réserve d’Ankoririka, 10.6 km 13° NE de Tsaramandroso (-16.26722°, 47.04861°), tropical dry forest, 210 m alt.; Parc National d’Ankarafantsika, Ampijoroa Station Forestière, 5.4 km 331° NW Andranofasika (-16.29889°, 46.813°), tropical dry forest, 70 m alt.; Parc National de Namoroka, 16.9 km 317° NW Vilanandro (-16.40667°, 45.31°), tropical dry forest, 100 m alt.; 9.8 km 300° WNW Vilanandro (-16.46667°, 45.35°), tropical dry forest, 140 m alt.; Réserve Spéciale de Bemarivo, 23.8 km 223° SW Besalampy (-16.925°, 44.36833°), tropical dry forest, 30 m alt.; Réserve forestière Beanka, 54.3 km E Maintirano (-18.06009°, 44.54086°), tropical dry forest on tsingy, 262 m alt.; Parc National Tsingy de Bemaraha, 10.6 km ESE 123° Antsalova (-18.70944°, 44.71817°), tropical dry forest on tsingy, 150 m alt.; 2.5 km 62° ENE Bekopaka, Ankidrodroa River (-19.13222°, 44.81467°), tropical dry forest on tsingy, 100 m alt.; 3.4 km 93° E Bekopaka, Tombeau Vazimba (-19.14194°, 44.828°), tropical dry forest, 50 m alt.; Bekopaka (-19.14667°, 44.796°), village, 100 m alt. Toliara. Forêt de Kirindy, 15.5 km 64° ENE Marofandilia (-20.045°, 44.66222°), tropical dry forest, 100 m alt.; 48 km ENE Morondava, Kirindy Forest (-20.06667°, 44.65°), tropical dry forest, 30 m alt.; (-20.07444°, 44.67611°), tropical dry forest, 100 m alt.; Parc National de Kirindy Mite, 16.3 km 127° SE Belo sur Mer (-20.79528°, 44.147°), tropical dry forest, 80 m alt.; Makay Mts. (-21.21836°, 45.3106°), gallery forest on sandy soil, 510 m alt.; (-21.21985°, 45.32396°), gallery forest on sandy soil, 500 m alt.; (-21.22284°, 45.32477°), gallery forest on sandy soil, 490 m alt.; (-21.22336°, 45.32628°), gallery forest on sandy soil, 480 m alt.; (-21.25978°, 45.17158°), gallery forest with palm and pandanus, 430 m alt.; Forêt de Beroboka, 5.9 km 131° SE Ankidranoka (-22.23306°, 43.36633°), tropical dry forest, 80 m alt.; 29 km NNW Ranohira, Isalo N.P. (-22.31614°, 45.29625°), gallery forest, 490 m alt.; Parc National de Zombitse, 19.8 km 84° E Sakaraha (-22.84333°, 44.71°), tropical dry forest, 770 m alt.; Réserve Spéciale Kalambatritra, Ampanihy (-23.4635°, 46.4631°), montane rainforest, 1270 m alt.; Forêt de Mite, 20.7 km 29° WNW Tongobory (-23.52417°, 44.12133°), gallery forest, 75 m alt.; Beza-Mahafaly, 27 km E Betioky (-23.65°, 44.63333°), tropical dry forest, 135 m alt.; 10 km NW Enakara, Rés. Andohahela (-24.56667°, 46.81667°), rainforest, 425 m alt.; Parc National Andohahela, Col de Tanatana, 33.3 km NW Tolagnaro (-24.7585°, 46.85367°), rainforest, 275 m alt.; Parc National d’Andohahela, Col du Sedro, 3.8 km 113° ESE Mahamavo, 37.6 km 341° NNW Tolagnaro (-24.76389°, 46.75167°), montane rainforest, 900 m alt.; Réserve Privé Berenty, Forêt de Bealoka, Mandraré River, 14.6 km 329° NNW Amboasary (-24.95694°, 46.2715°), gallery forest, 35 m alt.; Forêt de Malaza, Mandraré River, 8.6 km 314° NW Amboasary (-25.00778°, 46.306°), gallery forest, 40 m alt.; Grand Lavasoa, 25.9 km W Tolagnaro (-25.08767°, 46.749°), rainforest, 450 m alt. Fianarantsoa. SE of Fandriana, Korikory, (-20.38444°, 47.66722°), 1670 m alt.; 7 km W Ranomafana National Park (-21.25°, 47.41667°), montane rainforest, 900 m alt.; 8 km E Kianjavato, Vatovavy Forest (-21.38861°, 47.94361°), lowland forest, 145 m alt.; Andrambovato along river Tatamaly (-21.50967°, 47.40762°), cultivated land (tavy), 984 m alt.; Parc National d’Isalo, Sahanafa River, 29.2 km 351° N Ranohira (-22.31333°, 45.29167°), gallery forest, 500 m alt.; 30 km NNW Ranohira, Isalo N.P. (-22.31722°, 45.29333°), gallery forest, 455 m alt.; R.S. Ivohibe 8.0 km E Ivohibe (-22.48333°, 46.96833°), montane rainforest, 1200 m alt.; Forêt d’Analalava, 29.6 km 280° W Ranohira (-22.59167°, 45.12833°), tropical dry forest, 700 m alt.; Forêt de Vevembe, 66.6 km 293° Farafangana (-22.791°, 47.18183°), rainforest, transition to montane forest, 600 m alt.; Réserve Speciale Manombo 24.5 km 228° Farafangana (-23.01583°, 47.719°), rainforest, 30 m alt.

COMOROS. Grande Comore. Grillé (-11.47578°, 43.34669°), montane rainforest, 995 m alt.; Karthala (-11.82699°, 43.4295°), montane rainforest, 1000 m alt. Mohéli. Ouallah (-12.29696°, 43.67392°), rainforest, 680 m alt.; (-12.32717°, 43.65952°), coastal scrub, 10 m alt. Anjouan. (-12.30537°, 44.45031°), along roadside, mango, banana, 500 m alt.

###### Remarks.

*Mystrium mysticum* females can be distinguished easily from other *Mystrium* females by a combination of a distinct longitudinal carina on the central portion of the labrum (Fig. 8A) or a single lateral spine on abdominal sternum VII (Fig. 8C), and the convexity of the anterior margin of the clypeus (Fig. 9C). In addition to these diagnostic characters, a combination of unsculptured and smooth areas on the ventral portion of the vertex (Fig. 11A) and the convexity of the anterior margin of the clypeus (as in Fig. 9C) separates *Mystrium mysticum* from the other *Mystrium* species in workers; in larger-sized alate queens (HW>2.00), simple setae on the pronotal dorsum (Fig. 9A) separate *Mystrium mysticum* from the queens of other species. For males, the small eye and small ocelli that are separated from each other by more than 3× the maximum diameter of lateral ocellus (Fig. 26B), petiolar dorsum (Fig. 28C) and abdominal tergum VIII (Fig. 28A) with rough and deep punctures, and well-developed basoventral expansion of aedeagus (Fig. 29A) separate *Mystrium mysticum* from the other *Mystrium* males in the Malagasy region.

Several diagnostic characters for *Mystrium mysticum* have been given in previous papers, (e.g. Forel 1891; Menozzi 1929); however, our comparative study revealed most of those characters could not cover the whole range of the morphological variation observed in *Mystrium* species. Here we propose a new set of diagnostic characters to identify *Mystrium mysticum*. The members of the *mysticum* species group display remarkable morphological variation in body size, relative mandibular length, relative shape of petiolar node in dorsal view, and body color. The available material revealed that the morphological variation within *Mystrium mysticum* is larger than the difference between *Mystrium mysticum* and the species it most resembles, *Mystrium rogeri*. For example, in order to separate *Mystrium rogeri* from *Mystrium mysticum*, Menozzi (1929) used the relatively wider shape of the petiolar node in dorsal view and the shape of setae (=hairs). However, in these two species the index of the petiolar node largely overlaps (PtI 153-183 vs. PtI 146-168) and the shape of setae varies gradually from spoon-shaped (small minor worker) to simple (large major worker) in single colony members. The separable characters for workers proposed in Forel (1895, 1899) cannot distinguish species in the *mysticum* species group. In fact, his syntypes for *Mystrium stadelmanni* consisted of minor workers of *Mystrium mysticum* and *Mystrium rogeri* (see below). In addition to the above variation, Molet et al. (2012) reported the presence of intercaste “mosaic monsters” in *Mystrium rogeri*, which are intermediate individuals having zero to three ocelli and mesosoma with incompletely developed wing sclerites. We confirmed the same intercastes in *Mystrium mysticum* as well, and these individuals will be keyed out with either workers or queens in our new identification key.

The redescription of the male of *Mystrium mysticum* in this study may be the first actual description of the male of this species. Previous male descriptions for *Mystrium mysticum* have been given in Forel (1891), Emery (1899), and Menozzi (1929). Of these descriptions, Forel (1891) and Menozzi (1929) are assumed to share the same specimen in ZMHB, Berlin. Although we have not examined the actual male specimen in ZMHB, the character description of the specimen does not fit the males in the *mysticum* species group. For example, the color of the abdomen is light brown in Forel (1891), and the eye occupies almost the entire lateral margin of the head in Menozzi (1929). Rather, those characters fit males in the *voeltzkowi* species group. We could not find useful information in Emery’s description (Emery 1899); however, he did not mention any distinct differences from known males at that point. We have already confirmed that the male in the type series of *Mystrium rogeri* is actually a male of *Mystrium oberthueri*, so only males of the *voeltzkowi* species group, i.e. *Mystrium voeltzkowi* and *Mystrium oberthueri* were known in 1899. Hence, we consider all male descriptions for *Mystrium mysticum* in previous studies unreliable.

Clarification of the male-worker association in *Mystrium mysticum* in this study marks a great advance; however, some problems remain, especially in the level of confidence in diagnosing males. External characters, e.g. strong and rough punctures on the petiolar dorsum (Fig. 28C) and abdominal tergite VIII (Fig. 28A), distinguish the *mysticum* species group from the other Malagasy species. However, a specific character is neither possible nor practical to distinguish *Mystrium mysticum* males from *Mystrium rogeri* males. In fact, although we proposed the aedeagal character to separate *Mystrium mysticum* and *Mystrium rogeri* (Fig. 29), this difference cannot be confirmed without dissection. Unexpected remarkable variation could exist in the genital character as well as the other external characters. Further and more thorough taxonomic examinations of males are necessary to find diagnostic characters able to separate these two species.

The neotype of *Mystrium mysticum* was designated in this study because we concluded that the original type specimens of this “mysterious species” are lost. Our study revealed that no one examined any of the original type specimens after the publication of the original description by Roger (1862). According to that paper, the description was made based on two queens, and at least one of these was alate. The two queens must have been deposited in the museum in Paris; however, we could not find these queens in the MNHN collection. Although the depository of *Mystrium mysticum* was given again in the original description of *Mystrium camillae* Emery, 1889, Emery did not provide any further information. Santschi (1914) discussed the differences between *Mystrium silvestrii* and *Mystrium mysticum* when he described *Mystrium silvestrii* as a new species; however, he only provided information about workers, not queens. Forel described five species in the genus *Mystrium* from 1895 to 1899, and he noted in the latest description for *Mystrium rogeri* Forel, 1899 that he never examined the actual syntype of *Mystrium mysticum*. In spite of these difficulties, we still conclude that the species having queens with simple setae is *Mystrium mysticum* for the following four reasons: (1) we recognize only two species in the *mysticum* species group from a large number of *Mystrium* specimens accumulated from the whole range of the Malagasy region; (2) when Forel (1899) described *Mystrium rogeri* only queens with simple setae were known from Madagascar and were constantly identified as *Mystrium mysticum*; (3) as Forel (1899) mentioned, Roger (1862) did not describe any specialized character for the queen’s setae; and (4) the queens of *Mystrium rogeri* have spatulate setae as Forel (1899) expected. In fact, the oldest queen specimen with spatulate setae, which is found in MNHN, was collected after Forel’s description (collection date is 1919).

A queen was chosen as the neotype because the original syntypes were two queens. The queen caste also offers a greater number of characters to distinguish this taxon from others in this species group. The location of the neotype was not based on a proximity to the original syntype series, but on the availably of a large collection series that included workers, males and queens.

*Mystrium stadelmanni* Forel, 1895 is synonymized with *Mystrium mysticum* in this study. *Mystrium stadelmanni* is described as a species having a longer petiole, narrower mesosoma, and less constricted abdomen compared with *Mystrium mysticum*; however, our observations reveal that the boundary of this species is doubtful. None of the diagnostic characters above represent more than intraspecific variation observed in a series of workers from a single colony of *Mystrium mysticum*. The fact that syntypes of *Mystrium stadelmanni* consist of two minor workers of *Mystrium mysticum* and one minor worker of *Mystrium rogeri* certifies that Forel’s determination (Forel 1895) was not sufficient to clarify proper species boundaries in this group. In addition to the above, we should note one more important point. Forel (1895) determined specimens in ZMHB to be the new species *Mystrium stadelmanni* based on comparisons with workers of *Mystrium rogeri* that he probably misidentified as *Mystrium mysticum*. Forel believed his specimens, which were given by Emery (Forel 1892), were *Mystrium mysticum* by that time (Forel 1892; 1895); however, he described *Mystrium rogeri* in 1899 by using workers from the same collection. Our conclusion of a misidentification of *Mystrium rogeri* is supported by his description of a “longer petiolar node in *Mystrium stadelmanni*” which is correct if Forel (1895) studied the two workers of *Mystrium mysticum* in ZMHB. According to our measurements, *Mystrium rogeri* has the shorter petiolar node (PtI 153-183) while *Mystrium mysticum* has the longer node (PtI 146-168). Hence we synonymize *Mystrium stadelmanni* with *Mystrium mysticum* by designating a *Mystrium mysticum* worker in the syntypes of *Mystrium stadelmanni* as the lectotype. Consequently, the description of *Mystrium stadelmanni* in Forel (1895) is determined to be the first actual description for the *Mystrium mysticum* worker.

Updating species boundaries and providing easier identification tools for *Mystrium mysticum* will help reorganize and connect existing ecological and phylogenetic information to proper species names in *Mystrium*. This taxonomic revision permits us to update identifications in recent ecological publications (Molet et al. 2009; Molet et al. 2007a; Molet et al. 2012), changing some of their identification from *Mystrium rogeri* to *Mystrium mysticum*: for example, five colonies (BLF00519, BLF04466, BLF04770, BLF06144, and BLF10994) out of 16 colonies listed as *Mystrium rogeri* in Table 1 of Molet et al. (Molet et al. 2007a) and out of material for Molet et al. (2009) are those of *Mystrium mysticum*; a queen of *Mystrium rogeri* in Figure 2 of Molet et al. (2009) and in Figure 1 and supplemental Figure A1 of Molet et al. (2012) is that of *Mystrium mysticum*. A worker of *Mystrium mysticum* was referred to as *Mystrium* ‘red’ in Figure 2 of Molet et al. (2009), as was supplemental Figure A1 of Molet et al. (2012) (see also in *Mystrium mirror*). On the other hand, “*Mystrium mysticum*” in the material of three recent phylogenetic papers (Brady et al. 2006; Kück et al. 2011; Moreau and Bell 2013; Ouellette et al. 2006) are *Mystrium voeltzkowi*, not *Mystrium mysticum*. “*Mystrium mysticum*” in Molet et al. (2009) is *Mystrium shadow*. Outellette et al. (2006) listed two more related undetermined specimens as *Mystrium mysticum*2 and *Mystrium mysticum*3; those are *Mystrium voeltzkowi* and *Mystrium mirror*, respectively.

##### Mystrium rogeri

Forel, 1899

http://species-id.net/wiki/Mystrium_rogeri

Figs 4A , 8A , 9B, 9D , 12B , 29B , 37C , 38C , 39C , 40C , 41C , 43B, 43D , 44B, 44D , 45B, 45D , 46B, 46D , 47B, 47D , 48B, 48D , 49B

Mystrium rogeri Forel, 1899 (in part: worker only, not male). MADAGASCAR. Syntypes: worker and male [The lectotype is designated below].
Mystrium rogeri : Menozzi 1929.
Mystrium mysticum [Not Roger, 1862]: Forel 1892, in part (worker only).
Mystrium stadelmanni : Forel 1895, in part.

###### Lectotype

**[here designated].** Worker: CASENT0101670, MADAGASCAR, Amparafaravantsiv Moramangoufer [MHNG: examined].

###### Worker.

**Description.** Measurements: lectotype. HL 1.87, HW 1.85, SL 1.13, ML 1.72, HD 1.24, WL 2.09, PnW 1.14, PpW 0.98, PtW 0.92, PtL 0.53, CI 98.7, SI 61.4, MI 93.3, PpI 86.5, PtI 174.6.

HL 1.19–2.27, HW 1.12–2.28, SL 0.70–1.37, ML 1.18–2.38, HD 0.83–1.47, WL 0.18–2.52, PnW 0.67–1.23, PpW 0.57–1.05, PtW 0.51–1.04, PtL 0.33–0.65, CI 93.9–101.4, SI 59.2–64.6, MI 90.9–105.7, PpI 79.3–90.1, PtI 153.1–182.6 (10 specimens measured).

Posterolateral corner of head strongly expanding posteriorly. Posterior face of vertex forming slightly blunt angle with dorsal face on median line of head, so that declivity of vertex on lateral part slightly steeper than on median part. Ventral half of vertex smooth and not sculptured. Eye small to moderately small. Anterior margin of clypeus straight or weakly concave and with small conical setae. Genal tooth of head undeveloped, but anterolateral corner of head angulate. Masticatory margin of mandible narrowly visible in full-face view, and difference in width of dorsal surface of mandible relatively small between mandibular shaft and distal portion. Second maxillary palpomere longer than third. First flagellomere (third antennal segment) as long as pedicel (second antennal segment). Shallow and fine longitudinal striae irregularly impressed on central part of pronotal dorsum, and sometimes shallowly reticulated around those striae. On lateral surface of pronotum, deep, fine longitudinal striae impressed and wide and deep punctures arranged on central horizontal line. Mesonotum differentiated from propodeum in dorsal view, length as long as that of propodeum in large individuals, shorter than propodeum in small individuals. Metanotal groove shallowly impressed, mesonotum in lateral view as high as pronotum in large individuals, higher than pronotum in small individuals. Metanotum weakly developed in largest individuals. Metapleural gland bulla developed, so that propodeal declivity in lateral view weakly convex posteriorly on ventral portion. Petiole in dorsal view moderately narrow, relatively narrower than that of *Mystrium mysticum*.

Body color brown to black.

###### Queen. 

**Description.** Measurements: HL 1.94–2.31, HW 1.99–2.40, SL 1.20–1.42, ML 1.87–2.48, HD 1.29–1.51, WL 2.78–3.31, MnW 1.42–1.83, PtW 1.03–1.32, PtL 0.61–0.78, CI 99.8–104.2, SI 58.1–62.8, MI 90.4–103.1, MnI 68.1–76.1, PtI 155.3–185.1 (10 specimens measured).

Wings usually present and well developed; but lacking in intercaste. Wing sclerites fully developed even if wings have dropped off in queen; developed to undeveloped in variable degrees in intercaste. Posterolateral corner of head strongly expanding posteriorly, expansion not differentiated from that of workers. Posterior face of vertex forming slightly blunt angle with dorsal face on median line of head, so that declivity of vertex on lateral part slightly steeper than on median part. Ventral half of vertex smooth and not sculptured. Eye well developed. Both anterior and lateral ocelli clearly present in queen; ocelli varied from absent to developed in intercaste. Anterior margin of clypeus straight or weakly concave with small conical setae. Anterolateral portion of head weakly to strongly angulate or with short spine. Masticatory margin of mandible narrowly, slightly visible in full-face view, dorsal surface on distal portion moderately wider than on mandibular shaft, difference in width much smaller than that in *Mystrium mysticum*. Teeth always present on whole mandibular shaft. Spatulate seta present on basal side of each basal denticle on masticatory margin of mandible. First flagellar segment on antenna as long as pedicel. Setae on pronotum spatulate, widened distally with sharp or blunt apex. Propodeal declivity in lateral view almost straight and forming right to slightly obtuse angle with its dorsal margin, of which ventral portion with small convexity by metapleural gland. Petiole relatively long in dorsal view, about 0.6× length of abdominal segment III.

Body color black.

###### Male.

**Description.** Measurements: HL 1.10–1.41, HW 1.43–1.85, SL 0.25–0.34, EL 0.59–0.76, WL 2.27–3.03, MnW 1.46–1.96, CI 127.9–132.0, SI 16.2–20.0, EI 49.9–54.1, MnI 101.4-107.6 (6 specimens measured).

Eye relatively small, occupying about half of head length. Ocelli relatively distant from dorsal margin of head in full-face view, or just failing to reach dorsal margin. Dorsal margin of head in full-face view rounded. Both anterior and lateral ocelli small. Distance between lateral ocellus and eye long, 3× longer than diameter of lateral ocellus. Posterior half of vertex clearly differentiated from dorsal half, dorsal face almost as long as posterior face. Palpal formula 4,3. First segment of maxillary palp flattened and distinctly wider than second segment. Second maxillary palpomere longer than third. Notauli clearly impressed on mesoscutum. Petiole in dorsal view thin, its length about 0.5-0.65× that of abdominal tergite III. Petiolar dorsum covered with rough, deep punctures. Abdominal tergum VIII roughly and deeply punctured.

Abdominal sternum IX punctured on its distal portion. Basal ring short, not extending basally. Telomere extending slightly further distally than digitus. Basoventral expansion of aedeagus less developed basoventrally, as long as dorsal extension. Ventral margin of aedeagus gently curved ventrally in lateral view. Aedeagus weakly narrowing distally, its distal portion widely rounded.

On forewing, cu-a located at junction of Media (M) and Cubitus (Cu).

Body color reddish brown to black.

###### Distribution.

MADAGASCAR and COMOROS: as in Figure 49B.

###### Additional material examined.

In addition to the type material, specimens from the following localities were examined in this study: MADAGASCAR. Diego-Suarez. Sakalava Beach [vegetated beach dunes] (-12.26278°, 49.3975°), across sandy trail in dwarf littoral forest, 10 m alt.; 7 km N Joffreville [camp 2 of Fisher] (-12.33333°, 49.25°), in dry forest, 360 m alt.; Parc National Montagne d’Ambre [lemur trail] (-12.51667°, 49.18333°), rainforest, 975 m alt.; Parc National Montagne d’Ambre [Petit Lac road] (-12.52028°, 49.17917°), rainforest, 1125 m alt.; Antsiranana. Parc National Montagne d’Ambre, Antomboka (-12.51269°, 49.17807°), montane rainforest, 970 m alt.; Parc National Montagne d’Ambre (-12.51389°, 49.17784°), montane rainforest, 984 m alt.; Pic Bades (-12.5186°, 49.18625°), montane rainforest, 900 m alt.; (-12.52306°, 49.17901°), montane rainforest, 1100 m alt.; Roussettes (-12.52574°, 49.17238°), montane rainforest, 1025 m alt.; Roussettes Camp 7 km SW Park entrance (-12.51444°, 49.18139°), rainforest, 960 m alt.; Mahasarika (-12.53176°, 49.17662°), montane rainforest, 1135 m alt.; (-12.53417°, 49.17607°), montane rainforest, 1325 m alt.; 3.6 km 235° SW Joffreville (-12.53444°, 49.1795°), montane rainforest, 925 m alt.; Petit lac (-12.53664°, 49.17412°), montane rainforest, 1130 m alt.; Lac Maudit (-12.58502°, 49.15147°), montane rainforest, 1250 m alt.; 12.2 km 211° SSW Joffreville (-12.59639°, 49.1595°), montane rainforest, 1300 m alt.; Forêt de Binara, 9.1 km 233° SW Daraina (-13.26333°, 49.60333°), rainforest, 800 m alt.; (-13.26333°, 49.6°), montane rainforest, 1100 m alt.; Nosy Faly (-13.3624°, 48.49101°), open secondary vegetation, 15 m alt.; Galoko chain, Mont Galoko (-13.58487°, 48.71818°), rainforest, 520 m alt.; Ampasindava, Forêt d’Ambilanivy, 3.9 km 181° S Ambaliha (-13.79861°, 48.16167°), rainforest, 600 m alt.; R.S. Manongarivo, 12.8 km 228° SW Antanambao (-13.97667°, 48.42333°), rainforest, 780 m alt.; 14.5 km 220° SW Antanambao (-13.99833°, 48.42833°), montane rainforest, 1175 m alt.; Parc National de Marojejy, Manantenina River, 27.6 km 35° NE Andapa, 9.6 km 327° NNW Manantenina (-14.435°, 49.76°), rainforest, 775 m alt.; 28.0 km 38° NE Andapa, 8.2 km 333° NNW Manantenina (-14.43667°, 49.775°), rainforest, 450 m alt.; Antranohofa, 26.6 km 31° NNE Andapa, 10.7 km 318° NW Manantenina (-14.44333°, 49.74333°), montane rainforest, 1325 m alt.; Betaolana Forest, along Bekona River (-14.52996°, 49.44039°), rainforest, 880 m alt.; Forêt Ambanitaza, 26.1 km 347° Antalaha (-14.67933°, 50.18367°), rainforest, 240 m alt.; Toamasina. Montagne d’Anjanaharibe, 18.0 km 21° NNE Ambinanitelo (-15.18833°, 49.615°), rainforest, 470 m alt.; Res. Ambodiriana, 4.8 km 306°Manompana, along Manompana river (-16.67233°, 49.70117°), rainforest, 125 m alt.; Réserve Spéciale Ambatovaky, Sandrangato river (-16.7633°, 49.26692°), rainforest, 520 m alt.; (-16.7702°, 49.26638°), rainforest, 470 m alt.; (-16.77274°, 49.26551°), rainforest, 450 m alt.; Manakambahiny, near Vavatenina Forest (-17.46667°, 49.35°), alt.; Parc National de Zahamena (-17.73359°, 48.72625°), rainforest, 950 m alt.; Tetezambatana forest, near junction of Nosivola and Manakambahiny Rivers (-17.74298°, 48.72936°), rainforest, 860 m alt.; Onibe River (-17.75908°, 48.85468°), rainforest, 780 m alt.; Reserve Betampona, Camp Vohitsivalana, 37.1 km 338° Toamasina (-17.88667°, 49.2025°), rainforest, 520 m alt.; Réserve Nationale Intégrale Betampona, Betampona 35.1 km NW Toamasina (-17.91801°, 49.20074°), rainforest, 500 m alt.; Réserve Naturelle Betampona, 34.08 km 332° Toamasina (-17.91977°, 49.20039°), rainforest, 525 m alt.; Reserve Betampona, Camp Rendrirendry 34.1 km 332° Toamasina (-17.924°, 49.19967°), rainforest, 390 m alt.; F.C. Sandranantitra (-18.04833°, 49.09167°), rainforest, 450 m alt.; Station forestière Analamazaotra, Analamazaotra 1.3 km S Andasibe (-18.38466°, 48.41271°), montane rainforest, 980 m alt.; Ankerana (-18.40062°, 48.81311°), rainforest, 865 m alt.; Ankerana (-18.4017°, 48.80605°), montane forest, 1035 m alt.; (-18.4061°, 48.82029°), rainforest, 725 m alt.; (-18.40829°, 48.82107°), rainforest, 750 m alt.; (-18.4104°, 48.8189°), rainforest, 855 m alt.; F.C. Andriantantely (-18.695°, 48.81333°), rainforest, 530 m alt.; Corridor Forestier Analamay-Mantadia, Tsaravoniana (-18.75641°, 48.42195°), rainforest, 1036 m alt.; (-18.75737°, 48.42302°), rainforest, 1018 m alt.; (-18.76124°, 48.42134°), rainforest, 939 m alt.; (-18.76465°, 48.41938°), rainforest, 1039 m alt.; Ambohibolakely (-18.76087°, 48.37128°), rainforest, 1044 m alt.; (-18.76131°, 48.36437°), rainforest, 983 m alt.; (-18.77898°, 48.36375°), rainforest, 918 m alt.; Ambohibolakely (-18.77908°, 48.36628°), rainforest, 1014 m alt.; Ambatoharanana (-18.79956°, 48.4028°), rainforest, 1058 m alt.; (-18.80388°, 48.40506°), rainforest, 1013 m alt.; (-18.80398°, 48.40358°), rainforest, 1064 m alt.; (-18.80424°, 48.40081°), rainforest, 968 m alt.; (-18.80438°, 48.40735°), rainforest, 960 m alt.; Bevolota 17.1 km N Andasibe (-18.77071°, 48.43164°), montane rainforest, 995 m alt.; Analamay (-18.80623°, 48.33707°), montane rainforest, 1068 m alt.; Parc National d´ Andasibe-Mantadia, Forêt de Mantadia, 25.7 km 248° Moramanga (-18.81402°, 48.43028°), rainforest, 1040 m alt.; Ambatovy, 12.4 km NE Moramanga (-18.83937°, 48.30842°), montane rainforest, 1080 m alt.; Forêt Ambatovy, 14.3 km 57° Moramanga (-18.85083°, 48.32°), montane rainforest, 1075 m alt.; Torotorofotsy (-18.87082°, 48.34737°), montane rainforest, marsh edge, 1070 m alt.; Toliara. Réserve Spéciale d’Ambohijanahary, Forêt d’Ankazotsihitafototra, 35.2 km 312° NW Ambaravaranala (-18.26667°, 45.40667°), montane rainforest, 1050 m alt.; Makay Mts. (-21.20978°, 45.34184°), Gallery forest on sandy soil, 525 m alt.; (-21.21013°, 45.35462°), Gallery forest with bamboo, 540 m alt.; (-21.22336°, 45.32628°), Gallery forest on sandy soil, 480 m alt.; (-21.227°, 45.33222°), gallery forest on sandy soil, 475 m alt.; Forêt Ivohibe 55.6 km N Tolagnaro (-24.56167°, 47.20017°), rainforest, 650 m alt.; 11 km NW Enkara, Rés Andohahela (-24.56667°, 46.81667°), rainforest, 950 m alt.; Forêt Ivohibe 55.0 km N Tolagnaro (-24.569°, 47.204°), rainforest, 200 m alt.; 6 km SSW Eminiminy, Res. Andohahela (-24.73333°, 46.8°), wet forest, 330 m alt.; Rés. Andohahela, 6 km SSW Eminiminy (-24.73333°, 46.8°), rainforest, 330 m alt.; Parc National Andohahela, Col de Tanatana, 33.3 km NW Tolagnaro (-24.7585°, 46.85367°), rainforest, 275 m alt.; Manampanihy River, 5.4 km 113° ESE Mahamavo, 36.7 km 343° NNW Tolagnaro (-24.76389°, 46.76683°), rainforest, 650 m alt.; Col du Sedro, 3.8 km 113° ESE Mahamavo, 37.6 km 341° NNW Tolagnaro (-24.76389°, 46.75167°), montane rainforest, 900 m alt.; Forêt Mandena 8.5 km N Tolagnaro (-24.95267°, 47.0025°), littoral rainforest, 20 m alt.; Grand Lavasoa, 25.9 km W Tolagnaro (-25.08767°, 46.749°), rainforest, 450 m alt.; Toamasina. Res. Ambodiriana, 4.8 km 306° Manompana, along Manompana river (-16.67233°, 49.70117°), rainforest, 125 m alt.; Réserve Spéciale Ambatovaky, Sandrangato river (-16.7633°, 49.26692°), rainforest, 520 m alt.; (-16.7702°, 49.26638°), rainforest, 470 m alt.; (-16.77274°, 49.26551°), rainforest, 450 m alt.; Manakambahiny, near Vavatenina Forest (-17.46667°, 49.35°), alt.; Parc National de Zahamena (-17.73359°, 48.72625°), rainforest, 950 m alt.; Tetezambatana forest, near junction of Nosivola and Manakambahiny Rivers (-17.74298°, 48.72936°), rainforest, 860 m alt.; Onibe River (-17.75908°, 48.85468°), rainforest, 780 m alt.; Reserve Betampona, Camp Vohitsivalana, 37.1 km 338° Toamasina (-17.88667°, 49.2025°), rainforest, 520 m alt.; Réserve Nationale Intégrale Betampona, Betampona 35.1 km NW Toamasina (-17.91801°, 49.20074°), rainforest, 500 m alt.; Réserve Naturelle Betampona, 34.08 km 332° Toamasina (-17.91977°, 49.20039°), rainforest, 525 m alt.; Reserve Betampona, Camp Rendrirendry 34.1 km 332° Toamasina (-17.924°, 49.19967°), rainforest, 390 m alt.; F.C. Sandranantitra (-18.04833°, 49.09167°), rainforest, 450 m alt.; Station forestière Analamazaotra, Analamazaotra 1.3 km S Andasibe (-18.38466°, 48.41271°), montane rainforest, 980 m alt.; Ankerana (-18.40062°, 48.81311°), rainforest, 865 m alt.; Ankerana (-18.4017°, 48.80605°), montane forest, 1035 m alt.; (-18.4061°, 48.82029°), rainforest, 725 m alt.; (-18.40829°, 48.82107°), rainforest, 750 m alt.; (-18.4104°, 48.8189°), rainforest, 855 m alt.; F.C. Andriantantely (-18.695°, 48.81333°), rainforest, 530 m alt.; Corridor Forestier Analamay-Mantadia, Tsaravoniana (-18.75641°, 48.42195°), rainforest, 1036 m alt.; (-18.75737°, 48.42302°), rainforest, 1018 m alt.; (-18.76124°, 48.42134°), rainforest, 939 m alt.; (-18.76465°, 48.41938°), rainforest, 1039 m alt.; Ambohibolakely (-18.76087°, 48.37128°), rainforest, 1044 m alt.; (-18.76131°, 48.36437°), rainforest, 983 m alt.; (-18.77898°, 48.36375°), rainforest, 918 m alt.; Ambohibolakely (-18.77908°, 48.36628°), rainforest, 1014 m alt.; Ambatoharanana (-18.79956°, 48.4028°), rainforest, 1058 m alt.; (-18.80388°, 48.40506°), rainforest, 1013 m alt.; (-18.80398°, 48.40358°), rainforest, 1064 m alt.; (-18.80424°, 48.40081°), rainforest, 968 m alt.; (-18.80438°, 48.40735°), rainforest, 960 m alt.; Bevolota 17.1 km N Andasibe (-18.77071°, 48.43164°), montane rainforest, 995 m alt.; Analamay (-18.80623°, 48.33707°), montane rainforest, 1068 m alt.; Parc National d´ Andasibe-Mantadia, Forêt de Mantadia, 25.7 km 248° Moramanga (-18.81402°, 48.43028°), rainforest, 1040 m alt.; Ambatovy, 12.4 km NE Moramanga (-18.83937°, 48.30842°), montane rainforest, 1080 m alt.; Forêt Ambatovy, 14.3 km 57° Moramanga (-18.85083°, 48.32°), montane rainforest, 1075 m alt.; Torotorofotsy (-18.87082°, 48.34737°), montane rainforest, marsh edge, 1070 m alt.; Toliara. Réserve Spéciale d’Ambohijanahary, Forêt d’Ankazotsihitafototra, 35.2 km 312° NW Ambaravaranala (-18.26667°, 45.40667°), montane rainforest, 1050 m alt.; Makay Mts. (-21.20978°, 45.34184°), gallery forest on sandy soil, 525 m alt.; (-21.21013°, 45.35462°), gallery forest with bamboo, 540 m alt.; (-21.22336°, 45.32628°), gallery forest on sandy soil, 480 m alt.; (-21.227°, 45.33222°), gallery forest on sandy soil, 475 m alt.; Forêt Ivohibe 55.6 km N Tolagnaro (-24.56167°, 47.20017°), rainforest, 650 m alt.; 11 km NW Enkara, Rés Andohahela (-24.56667°, 46.81667°), rainforest, 950 m alt.; Forêt Ivohibe 55.0 km N Tolagnaro (-24.569°, 47.204°), rainforest, 200 m alt.; 6 km SSW Eminiminy, Res. Andohahela (-24.73333°, 46.8°), wet forest, 330 m alt.; Rés. Andohahela, 6 km SSW Eminiminy (-24.73333°, 46.8°), rainforest, 330 m alt.; Parc National Andohahela, Col de Tanatana, 33.3 km NW Tolagnaro (-24.7585°, 46.85367°), rainforest, 275 m alt.; Manampanihy River, 5.4 km 113° ESE Mahamavo, 36.7 km 343° NNW Tolagnaro (-24.76389°, 46.76683°), rainforest, 650 m alt.; Col du Sedro, 3.8 km 113° ESE Mahamavo, 37.6 km 341° NNW Tolagnaro (-24.76389°, 46.75167°), montane rainforest, 900 m alt.; Forêt Mandena 8.5 km N Tolagnaro (-24.95267°, 47.0025°), littoral rainforest, 20 m alt.; Grand Lavasoa, 25.9 km W Tolagnaro (-25.08767°, 46.749°), rainforest, 450 m alt. Mahajanga. Réserve Spéciale Marotandrano, Marotandrano 48.3 km S Mandritsara (-16.28322°, 48.81443°), transition humid forest, 865 m alt. Fianarantsoa. Ranomafana Nat. Park, Miaranony Forest (-21.25°, 47.41667°), montane forest, 700 m alt.; 7 km W Ranomafana (-21.26667°, 47.41667°), montane rainforest, 1000 m alt.; Parc National de Ranomafana, Vatoharanana River, 4.1 km 231° SW Ranomafana (-21.29°, 47.43333°), montane rainforest, 1100 m alt.; 7.6 km 122° Kianjavato, Forêt Classée Vatovavy (-21.4°, 47.94°), rainforest, 175 m alt.; Andrambovato along river Tatamaly (-21.50967°, 47.40762°), cultivated land (tavy), 984 m alt.; (-21.51089°, 47.40987°), rainforest, 994 m alt.; 2 km W Andrambovato, along river Tatamaly (-21.51167°, 47.41°), montane rainforest, 1075 m alt.; 45 km S. Ambalavao (-22.21667°, 47.01667°), rainforest, 785 m alt.; 43 km S Ambalavao, Rés. Andringitra (-22.23333°, 47°), rainforest, 825 m alt.; 9.0 km NE Ivohibe (-22.42667°, 46.93833°), montane rainforest, 900 m alt.; Parc National d’Isalo, 9.1 km 354° N Ranohira (-22.48167°, 45.46167°), gallery forest, 725 m alt.; R.S. Ivohibe 8.0 km E Ivohibe (-22.48333°, 46.96833°), montane rainforest, 1200 m alt.; Forêt de Vevembe, 66.6 km 293° Farafangana (-22.791°, 47.18183°), rainforest, transition to montane forest, 600 m alt.; Réserve Speciale Manombo 24.5 km 228° Farafangana (-23.01583°, 47.719°), rainforest, 30 m alt.; Parc National Befotaka-Midongy, Papango 27.7 km S Midongy-Sud, Mount Papango (-23.83517°, 46.96367°), rainforest, 940 m alt.

COMOROS. Mohéli, Ouallah (-12.32717°, 43.65952°), coastal scrub, 10 m alt.

###### Remarks.

The females of *Mystrium rogeri* can be distinguished easily from other *Mystrium* females by the combination of a distinct longitudinal carina on the central portion of the labrum (Fig. 8A) or a single lateral spine on abdominal sternum VII (Fig. 8C), and the straight anterior margin of the clypeus (Fig. 9D). In addition to these diagnostic characters, a combination of unsculptured and smooth areas on the ventral portion of the vertex (as in Fig. 11A) and the straight anterior clypeal margin separates *Mystrium rogeri* from the other *Mystrium* species workers; and a larger-sized alate queen (HW>1.99) with spatulate setae on the pronotal dorsum (Fig. 9B) separate *Mystrium rogeri* from the queens of other species. For males, the small eye and small ocelli (as in Fig. 26B) that are separated from each other by a distance of more than 3× the maximum diameter of lateral ocellus, petiolar dorsum (as in Fig. 28C) and abdominal tergum VIII (as in Fig. 28A) with rough and deep punctures, and less developed basoventral expansion of aedeagus (Fig. 29B) separate *Mystrium rogeri* from the other *Mystrium* males in the Malagasy region.

We propose a new set of diagnostic characters in this study to correctly identify *Mystrium rogeri*. The diagnostic characters listed above are constant in females regardless of their body size. Because of the remarkable morphological variation in each species, most diagnostic characters for *Mystrium rogeri* proposed in previous papers (e.g. Emery 1899; Menozzi 1929) cannot separate *Mystrium rogeri* from the other *Mystrium* species, especially from *Mystrium mysticum*. *Mystrium rogeri* also displays distinct variation in body size, color, relative mandible length, and shape of the petiole. The difference in the petiolar shape (Emery 1899; Menozzi 1929) cannot be used for complete separation between *Mystrium rogeri* and *Mystrium mysticum* (PtI 153-183 vs. PtI 146-168). As mentioned in their discussion of *Mystrium mysticum*, Molet et al. (2012) reported “mosaic monster” intermediates between queen and worker. The existence of this intercaste is not unique to *Mystrium rogeri*, but rather a character of the *mysticum* species group that we confirmed in *Mystrium mysticum* as well. In this study, the intercastes are keyed out with either of the workers or queens of each species.

As we discussed in *Mystrium mysticum*, further studies are necessary to better separate the males of *Mystrium rogeri* and *Mystrium mysticum*. Here we propose only an aedeagal character to separate these very similar males (Fig. 29).

The lectotype for *Mystrium rogeri* is designated. *Mystrium rogeri* was described by Forel in 1899 using at least two workers and one male collected in Madagascar, and we confirm one worker and one male in his collection. Although we could not find any information to identify which type specimens were used in his original description (Forel 1899), Forel mentioned that the workers he determined as *Mystrium rogeri* were the same specimens he determined as *Mystrium mysticum* (Forel 1892), and which Emery had sent him. According to the description in Forel (1892), the worker of *Mystrium rogeri* is smaller and stouter than the worker of *Mystrium mysticum*; however, we could not deduce how he determined the boundary of “*Mystrium mysticum*” in the worker caste. To further complicate the story, when Forel described *Mystrium rogeri* in 1899, he probably determined workers of actual *Mystrium mysticum* as *Mystrium stadelmanni* (see remarks for *Mystrium mysticum*). Moreover, once he determined Emery’s material as *Mystrium rogeri*, the worker caste of *Mystrium mysticum* should have been considered “unknown” once again. In addition to the misidentification of the worker caste, his description did not provide any details to identify which male specimens he studied. We also confirm Forel’s suspicions (Forel 1899) that the male described as *Mystrium rogeri* is not conspecific with the worker. We verify that the male specimen in his collection, which was probably used for the description of *Mystrium rogeri*, is *Mystrium oberthueri*.

*Mystrium rogeri* is one of the most common *Mystrium* species used in recent ecological and phylogenetic studies (Molet et al. 2009; Molet et al. 2007a; Molet et al. 2012; Moreau et al. 2006; Saux et al. 2004). However, the identification of their voucher material is now reassessed: part of the material identified as *Mystrium rogeri* in Molet et al. (Molet et al. 2009; Molet et al. 2007a; Molet et al. 2012) is not *Mystrium rogeri* (see remarks under *Mystrium mysticum*).

#### *voeltzkowi* species group

The *voeltzkowi* species group is endemic to the Malagasy region, and consists of six species: *Mystrium eques* sp. n., *Mystrium janovitzi* sp. n., *Mystrium mirror* sp. n., *Mystrium oberthueri* Forel, 1897, *Mystrium shadow* sp. n., and *Mystrium voeltzkowi* Forel, 1897. *The complete lack of alate queens and the presence of ergatoid queens that are relatively smaller than conspecific workers* are unique characters in this group.

**Figure 50.**
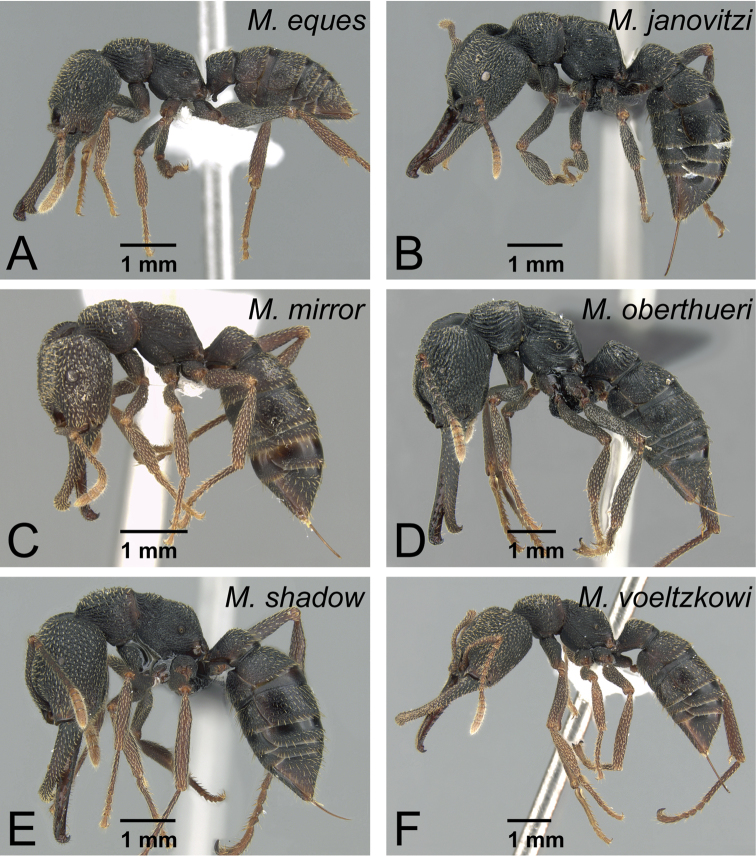
Workers of *voeltzkowi* species group in lateral view. **A** *Mystrium eques* sp. n. (CASENT0418314: holotype) **B** *Mystrium janovitzi* sp. n. (CASENT0482698: holotype) **C** *Mystrium mirror* sp. n. (CASENT0429897: holotype) **D** *Mystrium oberthueri* (CASENT0496066) **E** *Mystrium shadow* sp. n. (CASENT0318933: holotype) **F** *Mystrium voeltzkowi* (CASENT0002075).

In this study, we define ergatoid queens as the individuals having a mandible with an apical tooth that is moderately sclerotized and directed ventrally (Fig. 15B; Table 1). On the other hand, the workers are defined as individuals having a mandible with an apical tooth that is hardly sclerotized and curved inward (Fig. 15A). This definition for the ergatoid queen in the *voeltzkowi* species group is assumed on the basis of previous ecological reports by Molet et al. (Molet et al. 2007a; Molet et al. 2007b), although we did not confirm whether the “ergatoid queens” defined here are functional queens in all species. The morphological differences between workers and ergatoid queens in *voeltzkowi* species group are distinctly pronounced (as in Fig. 50F vs. 53F), which are shown in Molet et al. (2009) as well, compared with the differences observed between major and minor workers (as in Fig. 43B vs. 43D) in *camillae* and *mysticum* species groups (see also description of each group above). The body size of the ergatoid queens is usually smaller than that of workers in the same colony; however, the ranges largely overlap in species level. Both castes in some species display remarkable morphological variation in body size and shape, setae, or body sculpture, while the differences among species are often slight. The identification of specimens unassociated with the other castes or sexes is often problematic. Most specific differences are observed when individuals within the same body size range are compared.

##### Mystrium eques

Yoshimura & Fisher sp. n.

http://zoobank.org/0FF584D9-44A0-44C5-AF5E-BA17BF4ACE7B

http://species-id.net/wiki/Mystrium_eques

Figs 18B, 18D, 18F , 21A , 22A , 50A , 51A , 52A , 53A , 54A , 55A , 56A

###### Holotype.

Worker: CASENT0418314, BLF04691, MADAGASCAR, Toamasina, Tampolo, Masoala Peninsula, 40.4 km 154° SSE Maroantsetra (-15.73°, 49.96°), 30 m alt., 28.xi.–3.xii.2001, B.L.Fisher & H.J.Ratsirarson leg. [CASC].

###### Paratypes.

13 workers: CASENT0317390 [CASC], CASENT0317393 [CASC], CASENT0317394 [CASC], CASENT0317395 [CASC], CASENT0317396 [MSNG], CASENT0317397 [MHNG], CASENT0317398 [BMNH], CASENT0317399 [MCZC], CASENT0317443 [CASC], CASENT0418312 [MNHN], CASENT0418313 [CASC], CASENT0418316 [NHMB], CASENT0418318 [ZMHB]; 5 ergatoid queens: CASENT0418311 [CASC], CASENT0418315 [BMNH], CASENT0418317 [MHNG], CASENT0317391 [MCZC], CASENT0317392 [NHMB], with same data as holotype.

###### Worker.

**Description.** Measurements: holotype. HL 1.78, HW 1.65, SL 1.23, ML 1.87, HD 1.08, WL 2.01, PnW 0.94, PpW 0.83, PtW 0.85, PtL 0.57, CI 92.6, SI 74.4, MI 113.8, PpI 88.6, PtI 150.4.

HL 1.72–2.11, HW 1.59–2, SL 1.26–1.42, ML 1.81–2.18, HD 1.08–1.33, WL 2.07–2.34, PnW 0.92–1.1, PpW 0.83–1.02, PtW 0.83–1.03, PtL 0.54–0.67, CI 91.4–97.3, SI 70.4–78.9, MI 107.4–114.2, PpI 84.7–92.7, PtI 149.5–161.2 (10 specimens measured).

Posterolateral corner of head strongly to moderately expanding posteriorly. Posterior face of vertex forming almost a right angle with dorsal face on median line of head, so that declivity of vertex on lateral part as steep as that on median part. Whole region of vertex finely striated. Eye relatively smaller than that of *Mystrium oberthueri*. Anterior margin of clypeus strongly convex with long conical setae. Genal tooth of head relatively long, as long as lateral lobe of clypeus. Masticatory margin of mandible almost invisible in full-face view. Width of dorsal surface of mandible on distal portion slightly wider than that on mandibular shaft. Second maxillary palpomere longer than third. First flagellomere (third antennal segment) as long as pedicel (second antennal segment). Strong, deep and thick longitudinal striae regularly impressed on whole central part of pronotal dorsum. Strong, deep, and thick longitudinal striae impressed on lateral surface of pronotum. Mesonotum differentiated from propodeum in dorsal view, length slightly shorter than that of propodeum. Metanotal groove shallowly and gently impressed, and mesonotum higher than pronotum in lateral view. Metapleural gland bulla moderately developed, and propodeal declivity in lateral view almost straight. Petiole widened on posterior 1/4 and gently narrowed anteriorly in dorsal view, anterior margin straight to gently rounded and sometimes edged by thin striae.

Body color blackish brown. Appendages brighter, and four distal segments of antennal club yellowish.

###### Ergatoid queen.

**Description.** Measurements: HL 1.48–1.62, HW 1.41–1.57, SL 1.10–1.25, ML 1.38–1.56, HD 0.99–1.09, WL 1.95–2.10, PnW 0.85–0.95, PpW 0.83–0.94, PtW 0.87–0.97, PtL 0.52–0.56, CI 94.0–97.2, SI 77.7–83.9, MI 93.2–107.0, PpI 93.8–101.6, PtI 160.5–172.6 (7 specimens measured).

Wings vestigial and forming small but distinct appendages. Wing sclerites undeveloped. Posterolateral corner of head strongly expanding posteriorly, expansion relatively weaker than that of workers. Posterior face of vertex forming almost a right angle with dorsal face on median line of head, so that declivity of vertex on lateral part as steep as on median part. Ventral half of vertex sculptured. Eye small but distinct. Ocelli absent. Anterior margin of clypeus distinctly convex with long to short conical setae. Anterolateral portion of head with short spine. Masticatory margin of mandible almost invisible in full-face view. Dorsal surface of mandible on distal portion slightly wider than that on mandibular shaft. Spatulate or narrow spoon-shaped seta present on basal side of each basal denticle on masticatory margin of mandible. First flagellar segment on antenna about 1.0–1.2× length of pedicel. Setae on pronotum distinctly spatulate, widened distally with sharp or blunt apex. Metapleural gland bulla moderately developed, not expanding to dorsum of propodeal spiracle, so that propodeal declivity in lateral view weakly convex and rounded posteriorly on its ventral 1/3. Petiole relatively long in dorsal view, about 1.0–0.8× length of abdominal segment III.

Body color blackish to reddish brown.

###### Male

unknown.

**Figure 51.**
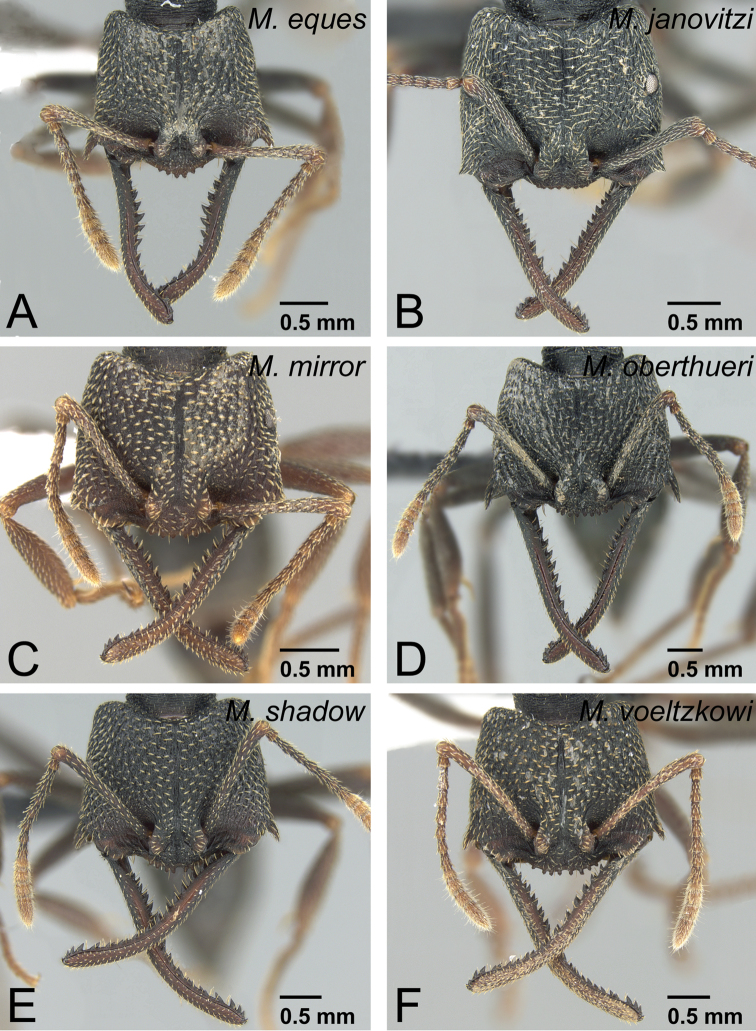
Head of the workers of *voeltzkowi* species group in full-face view. **A** *Mystrium eques* sp. n. (CASENT0418314: holotype) **B** *Mystrium janovitzi* sp. n. (CASENT0482698: holotype) **C** *Mystrium mirror* sp. n. (CASENT0429897: holotype) **D** *Mystrium oberthueri* (CASENT0496066) **E** *Mystrium shadow* sp. n. (CASENT0318933: holotype) **F** *Mystrium voeltzkowi* (CASENT0002075).

**Figure 52.**
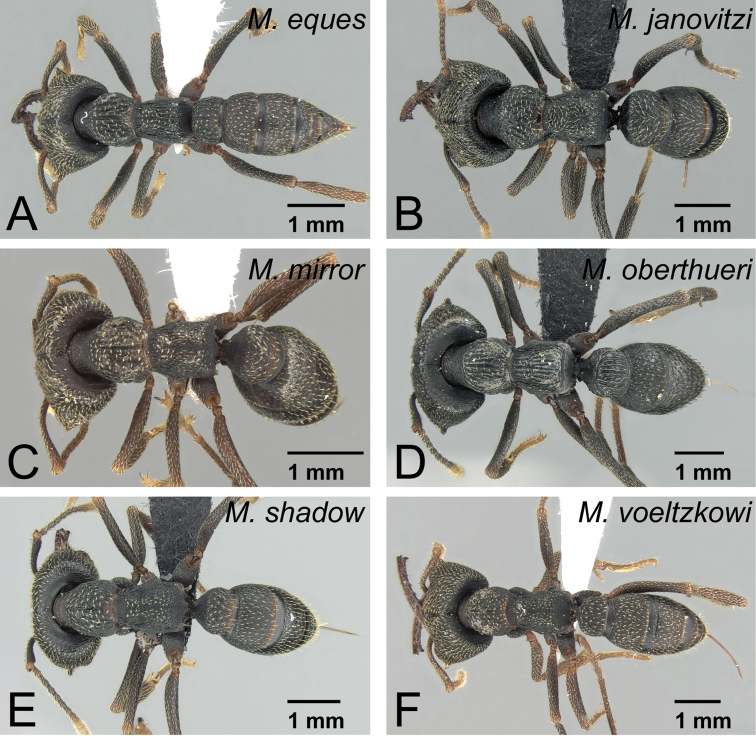
Workers of *voeltzkowi* species group in dorsal view. **A** *Mystrium eques* sp. n. (CASENT0418314: holotype) **B** *Mystrium janovitzi* sp. n. (CASENT0482698: holotype) **C** *Mystrium mirror* sp. n. (CASENT0429897: holotype) **D** *Mystrium oberthueri* (CASENT0496066) **E** *Mystrium shadow* sp. n. (CASENT0318933: holotype) **F** *Mystrium voeltzkowi* (CASENT0002075).

**Figure 53.**
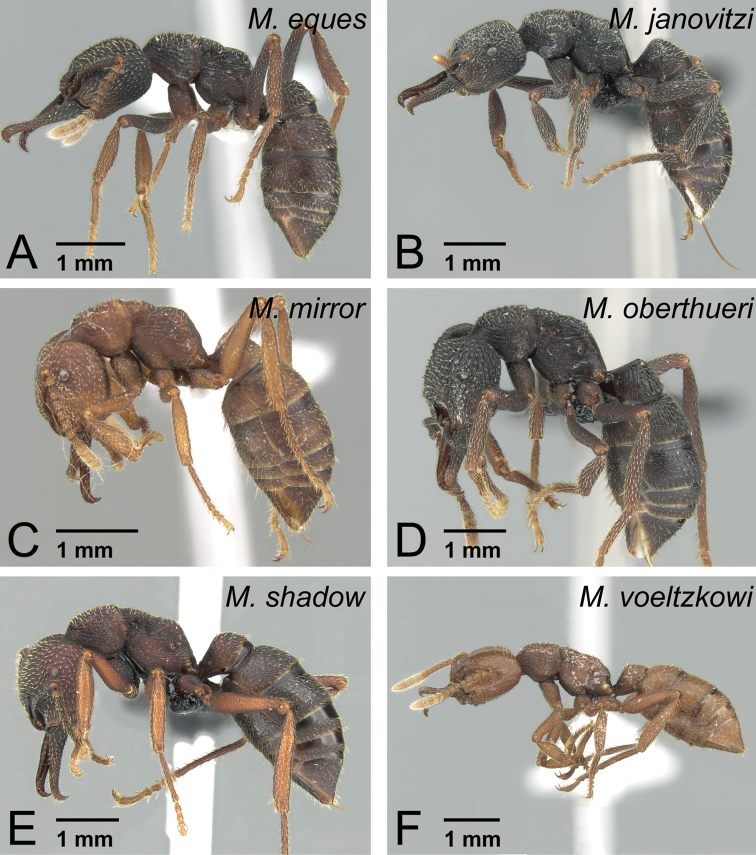
Ergatoid queens of *voeltzkowi* species group in lateral view. **A** *Mystrium eques* sp. n. (CASENT0418317: paratype) **B** *Mystrium janovitzi* sp. n. (CASENT0482696) **C** *Mystrium mirror* sp. n. (CASENT0318940: paratype) **D** *Mystrium oberthueri* (CASENT0496064) **E** *Mystrium shadow* sp. n. (CASENT0077647: paratype) **F** *Mystrium voeltzkowi* (CASENT0002092).

**Figure 54.**
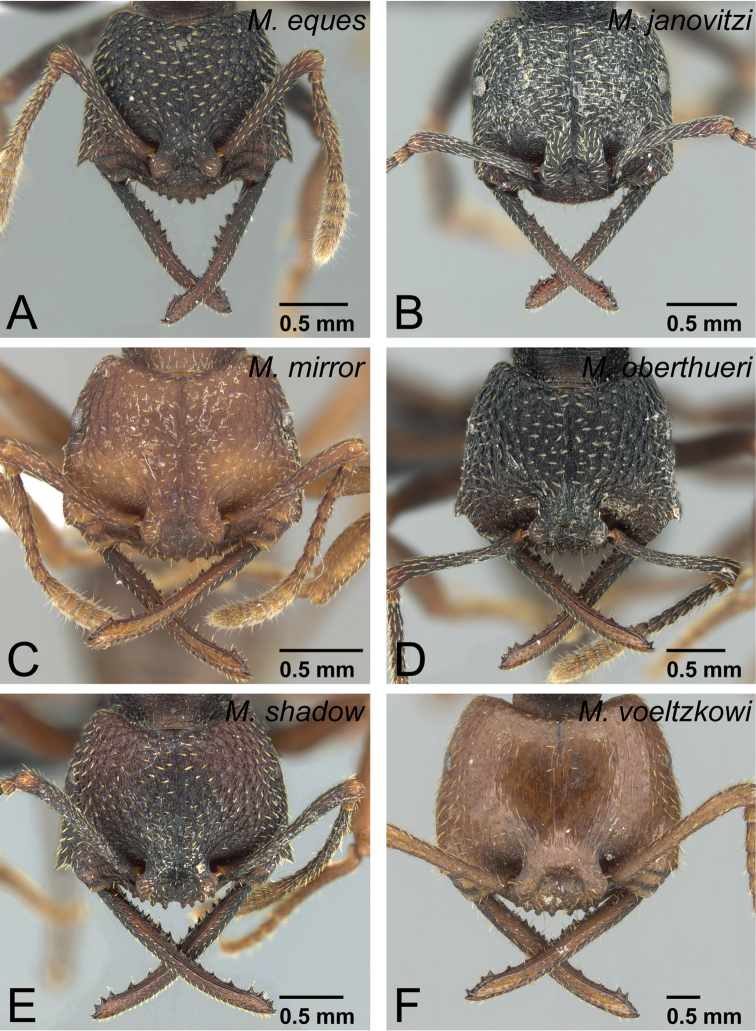
Head of the ergatoid queens of *voeltzkowi* species group in full-face view. **A** *Mystrium eques* sp. n. (CASENT0418317: paratype) **B** *Mystrium janovitzi* sp. n. (CASENT0482696) **C** *Mystrium mirror* sp. n. (CASENT0318940: paratype) **D** *Mystrium oberthueri* (CASENT0496064) **E** *Mystrium shadow* sp. n. (CASENT0077647: paratype) **F** *Mystrium voeltzkowi* (CASENT0002092).

**Figure 55.**
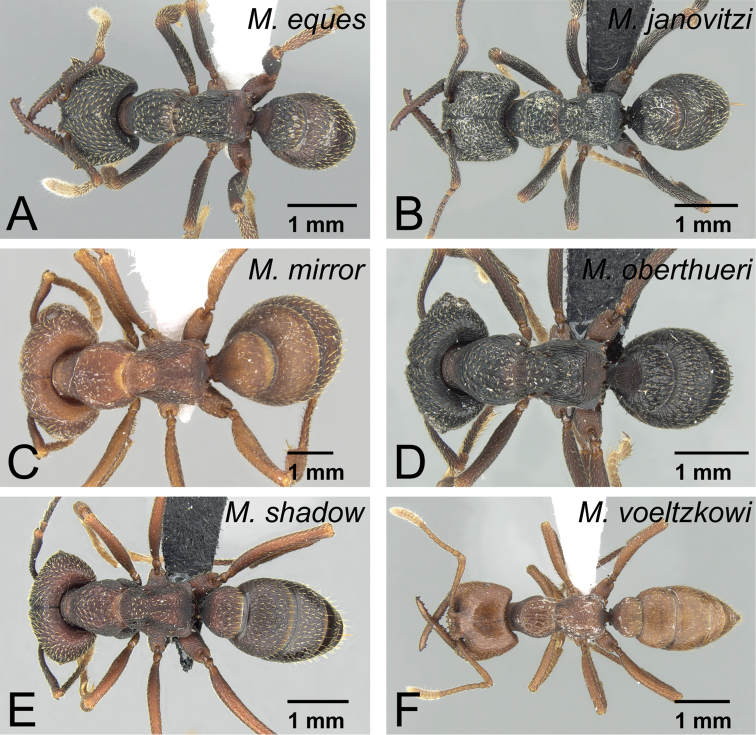
Ergatoid queens of *voeltzkowi* species group in dorsal view. **A** *Mystrium eques* sp. n. (CASENT0418317: paratype) **B** *Mystrium janovitzi* sp. n. (CASENT0482696) **C** *Mystrium mirror* sp. n. (CASENT0318940: paratype) **D** *Mystrium oberthueri* (CASENT0496064) **E** *Mystrium shadow* sp. n. (CASENT0077647: paratype) **F** *Mystrium voeltzkowi* (CASENT0002092).

**Figure 56.**
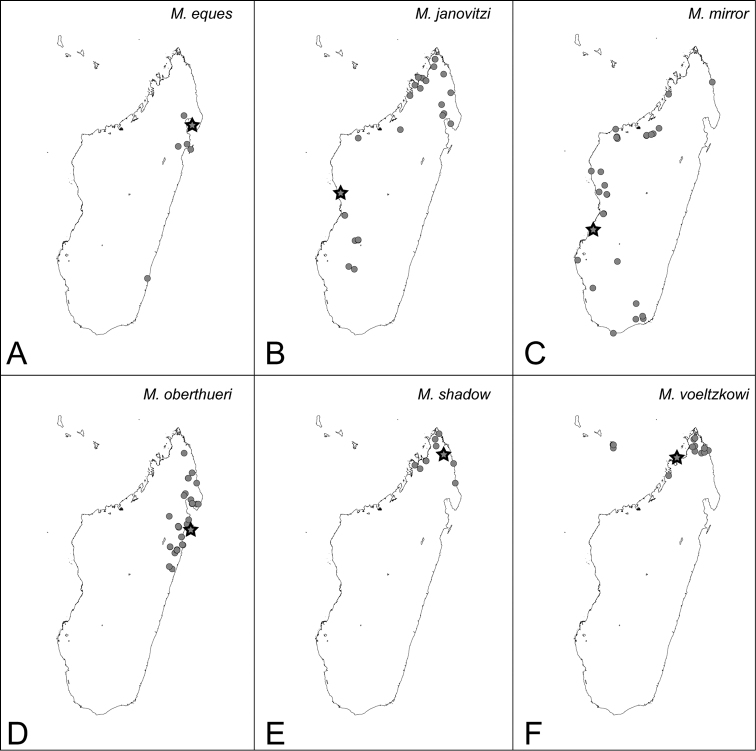
Distribution maps for *voeltzkowi* species group. **A** *Mystrium eques* sp. n. **B** *Mystrium janovitzi* sp. n. **C** *Mystrium mirror* sp. n. **D** *Mystrium oberthueri* **E** *Mystrium shadow* sp. n. **F** *Mystrium voeltzkowi*. Star symbols represent the type locality: the localities for *Mystrium oberthueri* and *Mystrium voeltzkowi* are approximate, which are assumed from the original descriptions.

###### Etymology.

This species name is the Latin word eques, inspired by the distinctly developed clypeus (shield) on the new species. The species epithet is a noun and invariant.

###### Distribution.

MADAGASCAR: as in Figure 56A.

###### Additional material examined.

In addition to the type material, specimens from the following localities were examined in this study: MADAGASCAR. Toamasina. Montagne d’Akirindro 7.6 km 341° NNW Ambinanitelo (-15.28833°, 49.54833°), rainforest, 600 m alt.; 5.3 km SSE Ambanizana, Andranobe (-15.66667°, 49.96667°), rainforest, 425 m alt.; Tampolo, Masoala Peninsula, 40.4 km 154° SSE Maroantsetra (-15.73°, 49.96°), rainforest, 30 m alt.; Res. Ambodiriana, 4.8 km 306° Manompana, along Manompana river (-16.67233°, 49.70117°), rainforest, 125 m alt.; Réserve Spéciale Ambatovaky, Sandrangato river (-16.77274°, 49.26551°), rainforest, 450 m alt.; Ile Sainte Marie, Forêt Kalalao, 9.9 km 34° Ambodifotatra (-16.9225°, 49.88733°), rainforest, 100 m alt.; Réserve Speciale Manombo 24.5 km 228° Farafangana (-23.01583°, 47.719°), rainforest, 30 m alt.

###### Remarks.

The worker of *Mystrium eques* is easily distinguished from those of other *Mystrium* species by a combination of the following characters: strong and deep longitudinal sculpture on the dorsal and lateral surface of pronotum (as in Fig. 17B); anteromedial portion of the clypeus strongly convex anteriorly (Fig. 18B); and sharper angle between the posterior and dorsal faces of the vertex on the median line (as in Fig. 16A). The queen of *Mystrium eques* is differentiated from other *Mystrium* queens by having vestigial wings reduced to small appendages (as in Fig. 25D) with undeveloped wing sclerites (ergatoid), posterior face of the vertex forming approximately a right angle with its dorsal face on the median line (Fig. 21A), and a strongly convex anterior clypeal margin (Fig. 22A).

The worker of *Mystrium eques* is similar to that of *Mystrium oberthueri*, however, the strongly convex anterior clypeal margin separates *Mystrium eques* (Fig. 18B) from *Mystrium oberthueri* (Fig. 18A), in which the anterior margin of the clypeus is straight. The male of *Mystrium eques* is not yet known. Because of the similarity between the workers and queens of *Mystrium eques* and *Mystrium oberthueri*, we assume the male of *Mystrium eques* is also similar to that of *Mystrium oberthueri*. The most easily distinguishable character between workers of *Mystrium eques* and *Mystrium oberthueri*, the anterior margin of the clypeus (Figs 18A, B), is unfortunately not useful for males because the anterior clypeal margin and the conical setae on it are less developed in males. *Mystrium eques* (Fig. 56A) has a sympatric distribution with *Mystrium oberthueri* (Fig. 56D), so collection locality does not separate these two similar species either. The current male specimens identified as *Mystrium oberthueri* may contain males of *Mystrium eques*. Further studies are necessary to resolve this issue.

##### Mystrium janovitzi

Yoshimura & Fisher sp. n.

http://zoobank.org/B390A568-7F20-45E0-95AF-74706CEAC3AB

http://species-id.net/wiki/Mystrium_janovitzi

Figs 4D , 5D , 17A, 17C , 23A, 23C , 26D , 28D , 31B , 32B, 32D, 37D , 38D , 39D , 40D , 41D , 50B , 51B , 52B , 53B , 54B , 55B , 56B

###### Holotype.

Worker: CASENT0482698, BLF04508, MADAGASCAR, Mahajanga, Forêt de Tsimembo, 11.0 km 346° NNW Soatana (-18.99528°, 44.4435°), 50 m alt., 21–25.xi.2001, Fisher-Griswold Arthropod Team leg. [CASC].

###### Worker.

**Description.** Measurements: holotype. HL 1.74, HW 1.89, SL 1.13, ML 1.69, HD 1.25, WL 2.15, PnW 1.2, PpW 0.99, PtW 1.07, PtL 0.73, CI 108.5, SI 59.8, MI 89.5, PpI 82.5, PtI 146.

HL 1.40–2.27, HW 1.46–2.67, SL 0.90–1.61, ML 1.30–2.58, HD 1.01–1.79, WL 1.83–2.94, PnW 0.91–1.52, PpW 0.80–1.28, PtW 0.82–1.32, PtL 0.56–0.90, CI 101.6–117.5, SI 59.7–67.9, MI 67.0–103.1, PpI 84.3–94.6, PtI 138.7–160.3 (10 specimens measured).

Posterolateral corner of head moderately expanding posteriorly. Posterior face of vertex forming almost right angle with dorsal face on median line of head, so that declivity of vertex on median part even steeper than on lateral part. Ventral half of vertex sculptured. Eye usually large, but sometimes small. Anterior margin of clypeus straight with small, extremely low conical setae. Genal tooth of head undeveloped, anterolateral corner of head angled. Masticatory margin of mandible almost invisible in full-face view, and width of dorsal surface of mandible almost identical from mandibular shaft to distal portion. Second maxillary palpomere longer than third. First flagellomere (third antennal segment) about as long as pedicel (second antennal segment). Shallow, rough, and fragmented longitudinal reticulation irregularly impressed along the entire pronotal dorsum. Shallow, rough, and fragmented longitudinal reticulation irregularly impressed on lateral surface of pronotum. Mesonotum often not differentiated from propodeum in dorsal view, length as long as that of propodeum. Metanotal groove indistinct in lateral view. Metapleural gland bulla moderately developed, and propodeal declivity in lateral view almost straight though sometimes weakly rounded. Petiole relatively long (PtI 138.7–160.3), with slightly widened posterior 1/4 to 1/3 in dorsal view, anterior margin gently rounded or almost straight, and never edged by striae.

Body color black. Four distal segments of antennal club brighter.

###### Ergatoid queen.

**Description.** Measurements: HL 1.32–1.77, HW 1.37–2.04, SL 0.99–1.34, ML 1.31–1.80, HD 0.99–1.35, WL 1.86–2.43, PnW 0.88–1.92, PpW 0.83–1.08, PtW 0.81–1.19, PtL 0.58–0.69, CI 103.9–114.8, SI 65.7–74.5, MI 88.5–95.3, PpI 48.6–95.1, PtI 139.6–177.3 (10 specimens measured).

Wings vestigial and reduced to small appendages, which are sometimes indistinct. Wing sclerites undeveloped. Posterolateral corner of head moderately expanding posteriorly, expansion not differentiated from that of workers. Posterior face of vertex forming almost right or even slightly acute angle with dorsal face on median line of head, so that declivity of vertex on median part even steeper than on lateral part. Ventral half of vertex sculptured. Eye varied from well developed to small. Ocelli absent. Anterior margin of clypeus straight with extremely short and small conical setae. Genal tooth of head absent, corner usually unangled, sometimes angled into a small, short spine. Masticatory margin of mandible almost invisible in full-face view, and dorsal surface on distal portion as wide as that on mandibular shaft. Spatulate seta present on basal side of each basal denticle on masticatory margin of mandible. First flagellar segment on antenna as long as pedicel. Setae on pronotum usually narrowing distally, or sometimes widened distally, with strongly sharpened apex. Metapleural gland bulla less developed, not expanding to either dorsum of propodeal spiracle or propodeal declivity margin in lateral view, so that posterior margin of propodeum in lateral view almost straight. Petiole relatively long in dorsal view, about 0.8-1.0× length of abdominal segment III.

Body color black.

###### Male.

**Description.** Measurements: HL 1.10–1.22, HW 1.52–1.72, SL 0.36–0.42, EL 0.62–0.71, WL 2.45–2.96, MnW 1.57–1.76, CI 135.2–141.0, SI 21.2–25.2, EI 54.1–58.1, MnI 99.3–103.9 (5 specimens measured).

Eye moderately large, occupying 0.6× head length. Ocelli reaching to dorsal margin of head in full-face view, or just failing to reach dorsal margin. Dorsal margin of head in full-face view rounded. Both anterior and lateral ocelli small. Lateral ocellus small and distant from eye: distance between these more than 2× maximum diameter of lateral ocellus. Posterior face of vertex rising steeply from neck, dorsal face slightly shorter than posterior face. Palpal formula 4,3. First segment of maxillary palp flattened and distinctly wider than second segment. Second maxillary palpomere longer than third. Notauli deeply to shallowly impressed on mesoscutum, but often unclear. Petiole in dorsal view thin, length 0.8–0.65× that of abdominal tergite III. Petiolar dorsum covered with shallow, sparse, irregular punctures, or almost smooth. Abdominal tergum VIII without deep punctures, almost smooth.

Distal portion of abdominal sternum IX smooth and not punctured. Basal ring long, distinctly extending basally. Telomere distinctly extending farther distally than digitus. Basoventral expansion of aedeagus moderately developed basoventrally, longer than dorsal extension. Ventral margin of aedeagus gently curved ventrally in lateral view. Aedeagus moderately narrowing distally on distal half, distal portion widely rounded.

On forewing, cu-a located far basal from junction of Media (M) and Cubitus (Cu).

Body color reddish brown to black.

###### Etymology.

The specific epithet is the patronym of Dr. Tyler Janovitz in recognition of his interest in myrmecology and his support for research on ants.

###### Distribution.

MADAGASCAR: as in Figure 56B.

###### Additional material examined.

In addition to the type material, specimens from the following localities were examined in this study: MADAGASCAR. Antsiranana. Ambohitra (Joffreville) (-12.53333°, 49.16667°), rainforest, 1100 m alt.; Parc National Montagne d’Ambre, 3.6 km 235° SW Joffreville (-12.53444°, 49.1795°), montane rainforest, 925 m alt.; Rés. Ankarana, 7 km SE Matsaborimanga (-12.9°, 49.11667°), rainforst, 150 m alt.; Forêt de Binara, 9.1 km 233° SW Daraina (-13.26333°, 49.60333°), rainforest, 800 m alt.; Nosy Be, Réserve Naturelle Intégrale de Lokobe, 6.3 km 112° ESE Hellville (-13.41933°, 48.33117°), rainforest, 30 m alt.; Forêt Ambato, 26.6 km 33° Ambanja (-13.4645°, 48.55167°), rainforest, 150 m alt.; Galoko chain, Mont Galoko (-13.58487°, 48.71818°), rainforest, 520 m alt.; (-13.58745°, 48.71419°), rainforest, 380 m alt.; Ampasindava, Forêt d’Ambilanivy, 3.9 km 181° S Ambaliha (-13.79861°, 48.16167°), rainforest, 600 m alt.; R.S. Manongarivo, 10.8 km 229° SW Antanambao (-13.96167°, 48.43333°), rainforest, 400 m alt.; Makirovana forest (-14.17066°, 49.95409°), rainforest, 225 m alt.; Forêt d’Anabohazo, 21.6 km 247° WSW Maromandia (-14.30889°, 47.91433°), tropical dry forest, 120 m alt.; 6.5 km SSW Befingotra, Rés. Anjanaharibe-Sud (-14.75°, 49.5°), rainforest, 875 m alt. Toamasina. Montagne d’Anjanaharibe, 18.0 km 21° NNE Ambinanitelo (-15.18833°, 49.615°), rainforest, 470 m alt.; Montagne d’Akirindro 7.6 km 341° NNW Ambinanitelo (-15.28833°, 49.54833°), rainforest, 600 m alt.; 6.3 km S Ambanizana, Andranobe (-15.6813°, 49.958°), rainforest, 5 m alt. Mahajanga. Forêt Ambohimanga, 26.1 km 314° Mampikony (-15.96267°, 47.43817°), tropical dry forest, 250 m alt.; Parc National de Namoroka, 17.8 km 329° WNW Vilanandro (-16.37667°, 45.32667°), tropical dry forest, 100 m alt.; Forêt de Tsimembo, 11.0 km 346° NNW Soatana (-18.99528°, 44.4435°), tropical dry forest, 50 m alt.; 8.7 km 336° NNW Soatana (-19.02139°, 44.44067°), tropical dry forest, 20 m alt. Toliara. 48 km ENE Morondava, Kirindy (-20.06667°, 44.65°), tropical dry forest, 30 m alt.; Forêt de Kirindy, 15.5 km 64° ENE Marofandilia (-20.06915°, 44.66041667°), tropical dry forest, 30 m alt.; Makay Mts. (-21.22284°, 45.32477°), gallery forest on sandy soil, 490 m alt.; (-21.25864°, 45.16412°), gallery forest with bamboo, 500 m alt.; Vohibasia Forest, 59 km NE Sakaraha (-22.46667°, 44.85°), tropical dry forest, 780 m alt.; Fianarantsoa. Forêt d’Analalava, 29.6 km 280° W Ranohira (-22.59167°, 45.12833°), tropical dry forest, 700 m alt.

###### Remarks.

The worker of *Mystrium janovitzi* is distinguished easily from that of the other *Mystrium* species by the combination of the following characters: the labrum lacking central longitudinal carina (as in Fig. 8B); low clypeal conical setae (Fig. 17C); the posterior face of the vertex forming a right angle with its dorsal face (as in Fig. 16A); wide pronotum covered with fine sculpture that is neither longitudinal nor reticulate (Fig. 17A). This combination of diagnostic characters for the worker is also useful for distinguishing ergatoid queens of *Mystrium janovitzi*. The ergatoid queen of *Mystrium janovitzi* is similar to conspecific workers in its sculpture and body color, but differs in having a narrower mandible with a straight apical tooth (as in Fig. 15B) and distinct vestigial wings (as in Fig. 25D). For the male, small ocelli not strongly protruding from posterior margin of head in full-face view (Fig. 31B), rounded posterior margin of head in full-face view, smooth abdominal tergum VIII (Fig. 28B), and vertex strongly rising from neck (Fig. 32B) separate *Mystrium janovitzi* from other known *Mystrium* males in the Malagasy region.

##### Mystrium mirror

Yoshimura & Fisher sp. n.

http://zoobank.org/BFEBDF95-71C8-48ED-BDDD-3763E4EE7C0C

http://species-id.net/wiki/Mystrium_mirror

Figs 5C , 8D , 20A, 20C, 20E , 24B , 25A, 25C , 26A, 26C , 27B, 27D , 37E , 38E , 39E , 40E , 41E , 50C , 51C , 52C , 53C , 54C , 55C , 56C

###### Holotype.

Worker: CASENT0429897, BLF04760, MADAGASCAR, Toliara, Parc National de Kirindy Mite, 16.3 km 127° SE Belo sur Mer (-20.79528°, 44.147°), 80 m alt., 6–10.xii.2001, Fisher-Griswold Arthropod Team [CASC].

###### Paratypes.

5 workers: CASENT0429898 [CASC], CASENT0318935 [BMNH], CASENT0318936 [MHNG], CASENT0318937 [MCZC], CASENT0318938 [NHMB]; 3 ergatoid queens: CASENT0429899 [CASC], CASENT0318939 [BMNH], CASENT0318940 [MHNG]; 4 males: CASENT0429893 [CASC], CASENT0429895 [BMNH], CASENT0318941 [MHNG], CASENT0318942 [MCZC], with same data as holotype.

###### Worker.

**Description.** Measurements: holotype. HL 1.60, HW 1.68, SL 1.19, ML 1.72, HD 1.03, WL 1.89, PnW 0.91, PpW 0.76, PtW 0.79, PtL 0.55, CI 104.7, SI 71.1, MI 102.7, PpI 83.2, PtI 143.8.

HL 1.16-1.91, HW 1.16–2.06, SL 0.94–1.48, ML 1.34–2.30, HD 0.84–1.33, WL 1.46–2.27, PnW 0.69–1.08, PpW 0.60–0.94, PtW 0.59–0.91, PtL 0.37–0.59, CI 99.5–108.3, SI 67.9–80.9, MI 103.8–115.7, PpI 81.5–92.6, PtI 143.7–163.2 (10 specimens measured).

Posterolateral corner of head strongly expanding posteriorly. Posterior face of vertex forming a blunt angle with its dorsal face on median line of head, so that declivity of vertex on lateral part distinctly steeper than on median part. Ventral half of vertex sculptured. Eye developed, relatively larger than that of *Mystrium voeltzkowi*. Anterior margin of clypeus straight to weakly convex with moderately long conical setae. Genal tooth of head weakly developed, reaching or slightly exceeding basal line of lateral lobe of clypeus. Masticatory surface of mandible in full-face view visible on basal half and invisible on distal half, width of dorsal surface of mandible almost identical from mandibular shaft to distal portion. Second maxillary palpomere longer than third. First flagellomere (third antennal segment) about 1.0-1.3× length of pedicel (second antennal segment). Pronotal dorsum covered with strong and longitudinal striae, center deeply impressed. Shallow but thick longitudinal striae impressed on lateral surface of pronotum. Mesonotum differentiated from propodeum in dorsal view, length shorter than that of propodeum. Metanotal groove shallowly and gently impressed, mesonotum higher than pronotum in lateral view. Metapleural gland bulla moderately developed, propodeal declivity in lateral view almost straight. Petiole gently narrowing from anterior 1/3 in dorsal view, anterior margin straight to gently rounded and not edged by striae.

Body color reddish brown to dark brown. Four distal segments of antennal club brighter.

###### Ergatoid queen.

**Description.** Measurements: HL 1.17–1.83, HW 1.19–1.55, SL 0.91–1.24, ML 1.12–1.50, HD 0.83–1.08, WL 1.52–2.02, PnW 0.70–0.87, PpW 0.69–0.87, PtW 0.72–0.92, PtL 0.38–0.49, CI 77.6–106.7, SI 72.0–81.9, MI 87.7–104.0, PpI 94.0–106.6, PtI 159.5–194.5 (10 specimens measured).

Wings usually vestigial and reduced to quite small appendages; sometimes completely absent. Wing sclerites undeveloped. Posterolateral corner of head strongly to weakly expanding posteriorly, expansion relatively weaker than that of workers. Vertex usually thin, forming blunt angle between posterior and dorsal faces on median line of head, so that declivity of vertex on lateral part distinctly steeper than on median part. Ventral half of vertex sculptured, or not differentiated from dorsal region. Eye moderate and distinct. Ocelli absent. Anterior margin of clypeus straight to weakly convex with small conical setae. Genal tooth of head absent and not angled, or angled into small, short spine. Masticatory margin of mandible almost invisible in full-face view, and dorsal surface on distal portion as wide as that on mandibular shaft. Spatulate seta present on basal side of each basal denticle on masticatory margin of mandible. First flagellar segment on antenna moderately long, about 1.1-1.2× length of pedicel. Setae on pronotum almost simple, narrowing distally with strongly sharpened apex. Metapleural gland bulla moderately developed and not expanding dorsally to propodeal spiracle, so that propodeal declivity in lateral view weakly convex and rounded posteriorly on its ventral 1/3. Petiole relatively long in dorsal view, about 0.6-0.8× length of abdominal segment III.

Body color yellowish brown, brown or reddish brown.

###### Male.

**Description.** Measurements: HL 0.93–1.05, HW 1.31–1.56, SL 0.29–0.35, EL 0.67–0.78, WL 1.90–2.38, MnW 1.23–1.40, CI 140.6–149.3, SI 21.5–23.1, EI 70.2–74.3, MnI 88.6–94.0 (6 specimens measured).

Eye quite large, occupying about 0.75× of head length. Ocelli protruding from dorsal margin of head in full-face view. Dorsal margin of head in full-face view rounded. Both anterior and lateral ocelli large. Distance between lateral ocellus and eye equal to or shorter than diameter of lateral ocellus. Posterior face of vertex clearly differentiated from dorsal face, dorsal face distinctly shorter than posterior face. Palpal formula 4,3. First segment of maxillary palp flattened and distinctly wider than second segment. Second maxillary palpomere longer than third. Notauli shallowly and weakly impressed on mesoscutum, but often unclear. Petiole in dorsal view thin, length 0.55–0.65× that of abdominal tergite III. Petiolar dorsum covered with fine punctures. Abdominal tergum VIII without deep punctures, almost smooth.

Distal portion of abdominal sternum IX smooth and not punctured. Basal ring short, not extending basally. Telomere distinctly extending distally farther than digitus. Basoventral expansion of aedeagus well developed basoventrally, distinctly longer than dorsal extension. Ventral margin of aedeagus almost straight in lateral view. Aedeagus distinctly narrowing distally, distal portion relatively sharp.

On forewing, cu-a located at junction of Media (M) and Cubitus (Cu), or slightly to far basal from junction.

Body color yellowish to reddish brown.

###### Etymology.

This species name is the English word mirror, inspired by the remarkable variation displayed in this species. This species might confuse an observer as a magic mirror would, which reflects different views to the observer. The species epithet is a noun and invariant.

###### Distribution.

MADAGASCAR: as in Figure 56C.

###### Additional material examined.

In addition to the type material, specimens from the following localities were examined in this study: MADAGASCAR. Antsiranana. Ambondrobe, 41.1 km 175° Vohemar (-13.71533°, 50.10167°), littoral rainforest, 10 m alt.; Forêt d’Anabohazo, 21.6 km 247° WSW Maromandia (-14.30889°, 47.91433°), tropical dry forest, 120 m alt.; Mahajanga. Forêt Ambohimanga, 26.1 km 314° Mampikony (-15.96267°, 47.43817°), tropical dry forest, 250 m alt.; Parc National de Baie de Baly, 12.4 km 337° NNW Soalala (-16.01°, 45.265°), tropical dry forest, 10 m alt.; Parc National d’Ankarafantsika, Forêt de Tsimaloto, 18.3 km 46° NE de Tsaramandroso (-16.22806°, 47.14361°), tropical dry forest, 135 m alt.; Ampijoroa Station Forestière, 5.4 km 331° NW Andranofasika (-16.29889°, 46.813°), tropical dry forest, 70 m alt.; Ampijoroa Station Forestière, 40 km 306° NW Andranofasika (-16.32083°, 46.81067°), tropical dry forest, 130 m alt.; Réserve d’Ankoririka, 10.6 km 13° NE de Tsaramandroso (-16.26722°, 47.04861°), tropical dry forest, 210 m alt.; Parc National de Namoroka, 17.8 km 329° WNW Vilanandro (-16.37667°, 45.32667°), tropical dry forest, 100 m alt.; 16.9 km 317° NW Vilanandro (-16.40667°, 45.31°), tropical dry forest, 100 m alt.; 9.8 km 300° WNW Vilanandro (-16.46667°, 45.35°), tropical dry forest, 140 m alt.; Réserve forestière Beanka, 50.2 km E Maintirano (-18.02649°, 44.05051°), tropical dry forest on tsingy, 250 m alt.; 52.7 km E Maintirano (-18.0622°, 44.52587°), tropical dry forest on tsingy, 300 m alt.; Parc National Tsingy de Bemaraha, 10.6 km ESE 123° Antsalova (-18.70944°, 44.71817°), tropical dry forest on tsingy, 150 m alt.; 2.5 km 62° ENE Bekopaka, Ankidrodroa River (-19.13222°, 44.81467°), tropical dry forest on tsingy, 100 m alt.; 3.4 km 93° E Bekopaka, Tombeau Vazimba (-19.14194°, 44.828°), tropical dry forest, 50 m alt.; Forêt de Tsimembo, 8.7 km 336° NNW Soatana (-19.02139°, 44.44067°), tropical dry forest, 20 m alt.; Toliara. Forêt de Kirindy, 15.5 km 64° ENE Marofandilia (-20.045°, 44.66222°), tropical dry forest, 100 m alt.; 48 km ENE Morondava, Kirindy (-20.06667°, 44.65°), tropical dry forest, 30 m alt.; Forêt de Beroboka, 5.9 km 131° SE Ankidranoka (-22.23306°, 43.36633°), tropical dry forest, 80 m alt.; Forêt de Mite, 20.7 km 29° WNW Tongobory (-23.52417°, 44.12133°), gallery forest, 75 m alt.; Forêt Vohidava 88.9 km N Amboasary (-24.24067°, 46.28783°), spiny forest/dry forest transition, 500 m alt.; Parc National d’Andohahela, Forêt de Manantalinjo, 33.6 km 63° ENE Amboasary, 7.6 km 99° E Hazofotsy (-24.81694°, 46.61°), spiny forest/thicket, 150 m alt.; Forêt d’Ambohibory, 1.7 km 61° ENE Tsimelahy, 36.1 km 308° NW Tolagnaro (-24.93°, 46.6455°), tropical dry forest, 300 m alt.; Réserve Privé Berenty, Forêt de Bealoka, Mandraré River, 14.6 km 329° NNW Amboasary (-24.95694°, 46.2715°), gallery forest, 35 m alt.; Réserve Spéciale de Cap Sainte Marie, 14.9 km 261° W Marovato (-25.59444°, 45.14683°), spiny forest/thicket, 160 m alt. Fianarantsoa. Parc National d’Isalo, Ambovo Springs, 29.3 km 4° N Ranohira (-22.29833°, 45.35167°), Uapaca woodland, 990 m alt.

###### Remarks.

The worker of *Mystrium mirror* is distinguished from workers of other *Mystrium* species by the combination of a central longitudinal furrow on the pronotal dorsum (as in Fig. 13B), reduced convexity of the anteromedial margin of the clypeus (Fig. 20A), blunter angle between the dorsal and posterior faces of the vertex on the median line of the head (as in Fig. 16B), and the straight posterior declivity of the propodeum (Fig. 20E). The workers of *Mystrium mirror* are quite similar to those of *Mystrium shadow* and *Mystrium voeltzkowi*, and the differences among the three species in workers are even slighter in small-sized individuals. When the comparison is made in workers within the same body size range, *Mystrium mirror* can be distinguished from *Mystrium shadow* by the smaller central clypeal conical setae (as in Fig. 19B), and from *Mystrium voeltzkowi* by either the straight declivity of the propodeum (Fig. 20E) and/or larger compound eye (Fig. 20C). The queen of *Mystrium mirror* can be distinguished from other *Mystrium* queens by a combination of the mesosoma without developed wing sclerites (ergatoid), posterior face of the vertex not strongly differentiated from its dorsal face (as in Fig. 21B), simpler body setae (Fig. 25A), a metapleural gland bulla that is moderately developed and not expanding dorsally to the propodeal spiracle (Fig. 24B), and brighter body color. The strength of the body sculpture is remarkably variable even among members in a single colony; some individuals may lack the striae and have a shiny pronotal dorsum - this weakness or lack of striae could prove to be an additional diagnostic character to separate this species from others. The ergatoid queens that are the most similar to *Mystrium mirror* are those of *Mystrium voeltzkowi* and *Mystrium shadow*. The less developed metapleural gland bulla (Fig. 24B) separates *Mystrium mirror* from *Mystrium voeltzkowi*, and pronotal setae simply narrowing distally with a sharp apex (Fig. 25A) separate it from *Mystrium shadow*. For the males, a large eye occupying almost 75% of the lateral margin of the head in full-face view (Fig. 26C), large lateral ocelli protruding from the dorsal margin of the head in full-face view (Fig. 38E), and a shorter distance between the eye and lateral ocellus (Fig. 26A) separate *Mystrium mirror* and *Mystrium voeltzkowi* from *Mystrium shadow*. However, only genital characters can distinguish *Mystrium mirror* from *Mystrium voeltzkowi* (Fig. 27).

The distribution range of *Mystrium mirror* (Fig. 56C) is allopatric with that of *Mystrium voeltzkowi* (Fig. 56F), but *Mystrium mirror* has a wider distribution than *Mystrium voeltzkowi*. Most of the specimens of *Mystrium mirror* were collected from tropical dry forests throughout most of western Madagascar (a few were collected in northern Madagascar), while most *Mystrium voeltzkowi* were collected from rainforest in the northern part of the country. This clear separation in distribution range and preferred habitat may be useful to roughly distinguish *Mystrium mirror* from *Mystrium voeltzkowi*.

We confirm that *Mystrium mirror* was previously used as material in one phylogenetic study (Ouellette et al. 2006) and two studies of evolutionary biology (Molet et al. 2009; Molet et al. 2012). In Ouellette et al. (2006), the specimen referred to as *M*. mysticum3 (CASENT0500395) is *Mystrium mirror*. An ergatoid queen of *Mystrium mirror* was referred to as *Mystrium* ‘red’ in figure 2 in Molet et al. (2009) and supplemental figure A1 in Molet et al. (2012). Prior to this study, the species boundaries among species in the genus *Mystrium* were too weak and ambiguous to provide proper taxonomic names for use in phylogenetic or ecological studies. However, the ecological data for *Mystrium* ‘red’ (Molet et al. 2009; Molet et al. 2007a; Molet et al. 2012) is still considered that of *Mystrium voeltzkowi* even though an image of *Mystrium mirror* was used in their figures. In fact, all colonies used as material in Molet et al. (2007a), which we could reexamine in the CASC collection, were identified as *Mystrium voeltzkowi* (see also *Mystrium voeltzkowi*).

##### Mystrium oberthueri

Forel, 1897

http://species-id.net/wiki/Mystrium_oberthueri

Figs 1A , 16A , 17B, 17D , 18A, 18C, 18E , 22B , 23B, 23D , 30A , 37F , 38F , 39F , 40F , 41F , 50D , 51D , 52D , 53D , 54D , 55D , 56D

Mystrium oberthueri Forel, 1897. MADAGASCAR. Syntypes: one worker (as major) and two ergatoid queens (as minor) [lectotype is designated below].
Mystrium oberthueri : Menozzi 1929 (in part?: uncertain male identification)
Mystrium oberthueri (Not) (male): Emery 1899.
Mystrium rogeri (male): Forel 1899.

###### Lectotype

**[here designated].** Worker: CASENT0101993, MADAGASCAR, Insel Ste. Marie (Island of Sainte Marie), Perrot [MHNG: examined].

###### Worker.

**Description.** Measurements: lectotype. HL 2.40, HW 2.61, SL 1.80, ML 2.92, HD 1.67, WL 2.79, PnW 1.39, PpW 1.21, PtW 1.18, PtL 0.81, CI 108.7, SI 69.0, MI 111.8, PpI 87.4, PtI 144.9.

HL 2.00–2.58, HW 2.12–2.76, SL 1.55–1.96, ML 2.39–3.17, HD 1.33–1.69, WL 2.43–2.96, PnW 1.17–1.38, PpW 1.02–1.29, PtW 1.00–1.32, PtL 0.67–0.81, CI 104.4–108.5, SI 68.8–74.9, MI 106.9–122.9, PpI 87.2–93.8, PtI 139.5–163.2 (10 specimens measured).

Posterolateral corner of head moderately expanding posteriorly. Posterior face of vertex forming almost right angle with dorsal face on median line of head, so that declivity of vertex on lateral part as steep as on median part. Whole region of vertex finely striated. Eye moderately small. Anterior margin of clypeus straight or weakly convex with long conical setae. Genal tooth of head relatively long, as long as lateral lobe of clypeus. Masticatory surface of mandible almost invisible in full-face view, and width of dorsal surface of mandible almost identical from mandibular shaft to distal portion. Second maxillary palpomere longer than third. First flagellomere (third antennal segment) about 1.2–1.5× length of pedicel (second antennal segment). Strong, deep longitudinal striae regularly impressed on whole central part of pronotal dorsum. Strong, deep longitudinal striae impressed on lateral surface of pronotum. Mesonotum differentiated from propodeum in dorsal view, length slightly shorter than that of propodeum. Metanotal groove shallowly and gently impressed in lateral view. Metapleural gland bulla moderately developed, and propodeal declivity in lateral view almost straight. Petiole widened on posterior 1/3 and gently narrowing anteriorly in dorsal view, anterior margin straight to gently rounded and often edged by thick striae.

Body color black.

###### Ergatoid queen.

**Description.** Measurements: HL 1.40–1.93, HW 1.46–1.96, SL 1.07–1.47, ML 1.49–2.00, HD 1.00–1.35, WL 1.92–2.56, PnW 0.83–1.11, PpW 0.85–1.13, PtW 0.81–1.07, PtL 0.53–0.70, CI 101.6–108.6, SI 71.6–76.7, MI 96.3–108.8, PpI 96.0–104.4, PtI 149.1–177.1 (10 specimens measured).

Wings vestigial and reduced to small but distinct appendages. Wing sclerites undeveloped. Posterolateral corner of head moderately expanding posteriorly, expansion not differentiated from that in workers. Posterior face of vertex forming almost a right angle with dorsal face on median line of head, so that declivity of vertex on lateral part as steep as on median part. Ventral half of vertex sculptured. Eye small but distinct. Ocelli absent. Anterior margin of clypeus almost straight with long to moderate conical setae. Anterolateral portion of head with short spine. Masticatory margin of mandible almost invisible in full-face view, and dorsal surface on distal portion as wide as that on mandibular shaft. Spatulate seta present on basal side of each basal denticle on masticatory margin of mandible. First flagellomere (third antennal segment) long, about 1.2-1.5× length of pedicel (second antennal segment). Setae on pronotum distinctly spatulate, widened distally with sharp or blunt apex. Metapleural gland bulla moderately developed, not expanding dorsally to propodeal spiracle, so that propodeal declivity in lateral view weakly convex and rounded posteriorly on its ventral 1/3. Petiole relatively long in dorsal view, about 0.8× length of abdominal segment III.

Body color blackish to reddish brown.

###### Male.

**Description.** Measurements: HL 1.44, HW 2.09, SL 0.62, EL 0.95, WL 3.55, MnW 1.94, CI 145.1, SI 29.6, EI 66.2, MnI 92.9 (1 specimen measured).

Eye moderately large, occupying 0.6× of head length. Ocelli relatively distant from dorsal margin of head in full-face view. Dorsal margin of head in full-face view straight. Both anterior and lateral ocelli small. Lateral ocellus small and distant from eye: distance between these more than 1.5× maximum diameter of lateral ocellus. Posterior face of vertex not clearly differentiated from dorsal face, so that vertex almost continuously rounded. Palpal formula 4,3. First maxillary palpomere flattened and distinctly wider than second segment. Second maxillary palpomere longer than third. Notauli clearly impressed on mesoscutum. Petiole in dorsal view thin, 0.8× as long as abdominal tergite III. Petiolar dorsum covered with shallow, irregular punctures. Abdominal tergum VIII without deep punctures, almost smooth.

Distal portion of abdominal sternum IX smooth and not punctured. Basal ring moderately long, expanding basoventrally. Telomere distinctly extending distally farther than digitus. Basoventral expansion of aedeagus moderately developed basoventrally, longer than dorsal extension. Ventral margin of aedeagus gently convex in lateral view. Aedeagus moderately narrowing distally and distal portion rounded.

On forewing, cu-a located far basal from junction of Media (M) and Cubitus (Cu).

Body color reddish brown to black.

###### Distribution.

MADAGASCAR: as in Figure 56D.

###### Additional material examined.

In addition to the type material, specimens from the following localities were examined in this study: MADAGASCAR. Antsiranana. Forêt d’ Antsahabe, 11.4 km 275° W Daraina (-13.21167°, 49.55667°), tropical dry forest, 550 m alt.; Makirovana forest (-14.17066°, 49.95409°), rainforest, 415 m alt.; Parc National de Marojejy, Manantenina River, 27.6 km 35° NE Andapa, 9.6 km 327° NNW Manantenina (-14.435°, 49.76°), rainforest, 775 m alt.; 28.0 km 38° NE Andapa, 8.2 km 333° NNW Manantenina (-14.43667°, 49.775°), rainforest, 450 m alt.; Marojejy R.N.I. #12 (-14.43583°, 49.76056°), rainforest, 610 m alt.; Forêt Ambanitaza, 26.1 km 347° Antalaha (-14.67933°, 50.18367°), rainforest, 240 m alt.; 1 km W Andampibe, Cap Masoala (-15.69361°, 50.18139°), lowland rainforest, 125 m alt.; Fotodriana, Cap Masoala (-15.69694°, 50.27028°), lowland rainforest, 25 m alt.; Toamasina. Montagne d’Anjanaharibe, 18.0 km 21° NNE Ambinanitelo (-15.18833°, 49.615°), rainforest, 470 m alt.; Montagne d’Akirindro 7.6 km 341° NNW Ambinanitelo (-15.28833°, 49.54833°), rainforest, 600 m alt.; 19 km ESE Maroantsetra (-15.48333°, 49.9°), rainforest, 350 m alt.; 5.3 km SSE Ambanizana, Andranobe (-15.66667°, 49.96667°), rainforest, 425 m alt.; 6.3 km S Ambanizana, Andranobe (-15.6813°, 49.958°), rainforest, 5 m alt.; 6.3 km S Ambanizana, Andranobe (-15.68131°, 49.958°), rainforest, 25 m alt.; Parc National Mananara-Nord, 7.1 km 261° Antanambe (-16.455°, 49.7875°), rainforest, 225 m alt.; Res. Ambodiriana, 4.8 km 306° Manompana, along Manompana river (-16.67233°, 49.70117°), rainforest, 125 m alt.; Réserve Spéciale Ambatovaky, Sandrangato river (-16.7633°, 49.26692°), rainforest, 520 m alt.; (-16.77274°, 49.26551°), rainforest, 450 m alt.; (-16.7755°, 49.26427°), rainforest, 430 m alt.; (-16.81745°, 49.2925°), rainforest, 400 m alt.; (-16.81753°, 49.29498°), rainforest, 360 m alt.; Ile Sainte Marie, Forêt Kalalao, 9.9 km 34° Ambodifotatra (-16.9225°, 49.88733°), rainforest, 100 m alt.; Réserve Forestière Tampolo, 95.2 km N Toamasina (-17.27808°, 49.42853°), littoral rainforest, 20 m alt.; S.F. Tampolo, 10 km NNE Fenoarivo Atn. (-17.2825°, 49.43°), littoral rainforest, 10 m alt.; Bridge at Onibi, NW of Mahavelona (-17.65°, 49.46667°), in clay forest; Mahavelona (Foulpointe) (-17.66667°, 49.5°), Forest; Parc National de Zahamena, Besaky River (-17.75244°, 48.85321°), rainforest, 760 m alt.; Sahavorondrano River (-17.75257°, 48.85725°), rainforest, 765 m alt.; Onibe River (-17.75908°, 48.85468°), rainforest, 780 m alt.; Reserve Betampona, Camp Vohitsivalana, 37.1 km 338° Toamasina (-17.88667°, 49.2025°), rainforest, 520 m alt.; Réserve Naturelle Betampona, 34.1 km 332° Toamasina (-17.916135°, 49.20185°), rainforest, 550 m alt.; Réserve Nationale Intégrale Betampona, Betampona 35.1 km NW Toamasina (-17.91801°, 49.20074°), rainforest, 500 m alt.; Reserve Betampona, Camp Rendrirendry 34.1 km 332° Toamasina (-17.924°, 49.19967°), rainforest, 390 m alt.; F.C. Sandranantitra (-18.04833°, 49.09167°), rainforest, 450 m alt.; F.C. Andriantantely (-18.695°, 48.81333°), rainforest, 530 m alt.; Sahafina forest 11.4 km W Brickaville (-18.81445°, 48.96205°), rainforest, 140 m alt. Mahajanga. Réserve Spéciale Marotandrano, Marotandrano 48.3 km S Mandritsara (-16.28322°, 48.81443°), transition humid forest, 865 m alt.

###### Remarks.

The worker of *Mystrium oberthueri* is easily distinguished from that of other *Mystrium* species by the combination of the following characters: strong, deep, and thick longitudinal sculpture on the dorsal and lateral surface of pronotum (Fig. 17B); straight anteromedial margin of the clypeus (Fig. 18A); a sharper angle between the dorsal and posterior faces of the vertex on the median line of the head (Fig. 16A). The queen of *Mystrium oberthueri* is differentiated from other queens of *Mystrium* species by the vestigial wings reduced to small appendages with undeveloped wing sclerites (ergatoid: as in Fig. 25D), the posterior face of the vertex forming approximately a right angle with its dorsal face on the median line of the head (as in Fig. 21A), straight anterior clypeal margin (Fig. 22B), and moderately developed clypeal conical setae (Fig. 23B). The male of *Mystrium oberthueri* can be separated from the other known *Mystrium* males by its smooth abdominal tergum VIII (as in Fig. 28), small lateral ocelli situated distant from posterior margin of head in full-face view, and straight dorsal margin of head in full-face view (Fig. 30A). We should note that the current male character diagnoses may not be able to distinguish between *Mystrium oberthueri* and *Mystrium eques*, which is not yet known from the male caste and may be quite similar to *Mystrium oberthueri*.

The lectotype for *Mystrium oberthueri* is designated. According to Forel (1897), a single major worker and multiple ergatoid queens (as minor workers) were used for the original description of *Mystrium oberthueri*. We confirmed one worker and one ergatoid queen in the Forel collection in MHNG and one ergatoid queen in MCZC, all of which are labeled with the location “Ste. Marie Madagascar.” The single worker was chosen as the new representative specimen.

Although worker descriptions for *Mystrium oberthueri* are reliable, male descriptions of this species in previous papers are problematic. The original description by Forel (1897) did not include males. Later male descriptions were given by Emery (1899) and Menozzi (1929). As in the cases of *Mystrium mysticum* and *Mystrium rogeri*, Emery’s and Menozzi’s descriptions of the male of *Mystrium oberthueri* seem to be based on specimens not associated with conspecific workers. We did not examine the actual male specimens they used, and cannot be sure that these descriptions are for the actual male of *Mystrium oberthueri*. Emery mentioned Forel’s description (Forel 1899) of *Mystrium rogeri* in the same paper. We have confirmed the male described as *Mystrium rogeri* was the actual *Mystrium oberthueri*; however, Emery (1899) did not mention this problem. Moreover, Emery listed smaller body size as a character able to distinguish the male of *Mystrium oberthueri* from that of *Mystrium mysticum*; however, according to our measurements, *Mystrium oberthueri* is the largest of the *Mystrium* males (*Mystrium oberthueri*: HW>2.00 mm vs. other known males: HW<1.88 mm). We believe that Emery’s male description for *Mystrium oberthueri* is doubtful, even though we have not been able to determine the males described in either *Mystrium oberthueri* or *Mystrium mysticum* in Emery (1899). Second, Menozzi (1929) examined all types in the Forel collection, including those of *Mystrium rogeri*. However, he did not mention anything in the male description of *Mystrium oberthueri* about the male in the syntypes of *Mystrium rogeri*. Hence, the male description of the *Mystrium oberthueri* in Menozzi (1929) is also not likely to be the true *Mystrium oberthueri*, although it probably is a male in the *voeltzkowi* species group.

We describe a new species, *Mystrium eques*, with a worker similar to that of *Mystrium oberthueri*. As we have already mentioned in the section of *Mystrium eques*, the unknown male of *Mystrium eques* is likely to be quite similar to that of *Mystrium oberthueri*, and the current male diagnosis for *Mystrium oberthueri* may be insufficient to distinguish these two males (see *Mystrium eques*).

Material identified as *Mystrium oberthueri* in Molet et al. (2009) and Bouchet et al. (2013) is confirmed here.

##### Mystrium shadow

Yoshimura & Fisher sp. n.

http://zoobank.org/2CF2C722-970D-4829-87ED-C58CBF036481

http://species-id.net/wiki/Mystrium_shadow

Figs 19A , 21B , 25B, 25D , 28D , 30B , 31D , 32A, 32C , 37G , 38G , 39G , 40G , 41G , 50E , 51E , 52E , 53E , 54E , 55E , 56E

###### Holotype.

Worker: CASENT0318933, BLF09661, MADAGASCAR, Antsiranana, Forêt de Binara, 9.1 km 233° SW Daraina (-13.26333°, 49.60333°), 650-800 m alt., 3.xii.2003, B.L. Fisher leg. [CASC].

###### Paratypes.

4 workers: CASENT0077646 [BMNH], CASENT0077648 [MHNG], CASENT0318920 [MCZC], CASENT0318931 [NHMB]; 3 ergatoid queens: CASENT0077647 [CASC], CASENT0318932 [BMNH], CASENT0318934 [MHNG], with same data as holotype.

###### Worker.

**Description.** Measurements: holotype. HL 2.11, HW 2.15, SL 1.60, ML 2.52, HD 1.43, WL 2.42, PnW 1.09, PpW 0.99, PtW 0.90, PtL 0.59, CI 101.9, SI 74.4, MI 116.9, PpI 91.1, PtI 152.6.

HL 1.36–2.25, HW 1.37–2.36, SL 1.00–1.78, ML 1.40–2.89, HD 0.90–1.49, WL 1.55–2.67, PnW 0.74–1.16, PpW 0.67–1.03, PtW 0.72–1.04, PtL 0.40–0.66, CI 97.0–104.9, SI 70.5–76.8, MI 102.3–125.5, PpI 85.6–93.6, PtI 147.3–179.4 (10 specimens measured).

Posterolateral corner of head strongly expanding posteriorly. Posterior face of vertex forming blunt angle with dorsal face on median line of head, so that declivity of vertex on lateral part distinctly steeper than on median part. Ventral half of vertex sculptured. Eye moderately small. Anterior margin of clypeus strongly convex with long conical setae. Genal tooth of head moderately developed, reaching to about half of lateral lobe of clypeus. Masticatory surface of mandible in full-face view slightly visible on basal half and invisible on distal half, width of dorsal surface of mandible almost identical from mandibular shaft to distal portion. Second maxillary palpomere longer than third. First flagellomere (third antennal segment) about 1.0-1.3× length of pedicel (second antennal segment). Pronotal dorsum covered with strong longitudinal striae, which are waved and irregular, but median stria always deeply, straightly and clearly impressed. Strong, deep, and irregular longitudinal striae impressed on lateral surface of pronotum. Mesonotum differentiated from propodeum in dorsal view, length shorter than that of propodeum. Metanotal groove shallowly and gently impressed, mesonotum higher than pronotum in lateral view. Metapleural gland bulla moderately developed, and propodeal declivity in lateral view almost straight but sometimes weakly rounded. Petiole relatively short (PtI ), gently narrowing from anterior 1/3 in dorsal view, anterior margin straight to gently rounded and not edged by striae.

Body color reddish brown to black. Four distal segments of antennal club brighter.

###### Ergatoid queen.

**Description.** Measurements: HL 1.30–1.67, HW 1.31–1.70, SL 1.00–1.42, ML 1.30–1.81, HD 0.93–1.20, WL 1.74–2.33, PnW 0.76–1.00, PpW 0.76–0.98, PtW 0.76–0.96, PtL 0.45–0.61, CI 98.7–105.3, SI 76.2–85.5, MI 97.1–108.2, PpI 95.5–101.7, PtI 157.7–178.6 (10 specimens measured).

Wings vestigial and reduced to small but distinct appendages. Wing sclerites undeveloped. Posterolateral corner of head weakly expanding posteriorly, expansion distinctly weaker than that in workers. Posterior face of vertex forming blunt angle with dorsal face on median line of head, so that declivity of vertex on lateral part distinctly steeper than on median part. Ventral half of vertex sculptured. Eye small but distinct. Ocelli absent. Anterior margin of clypeus widely convex with short conical setae, sometimes with median notch. Genal tooth of head with short spine. Masticatory margin of mandible almost invisible in full-face view, dorsal surface on distal portion as wide as that on mandibular shaft. Spatulate seta present on basal side of each basal denticle on masticatory margin of mandible. First flagellomere (third antennal segment) moderately long, about 1.5–1.2× length of pedicel (second antennal segment). Setae on pronotum widened distally with sharp apex. Metapleural gland bulla moderately developed, not expanding to dorsum of propodeal spiracle nor propodeal declivity margin in lateral view, so that posterior margin of propodeum in lateral view almost straight. Petiole relatively long in dorsal view, about 0.5–0.8× length of abdominal segment III.

Body color reddish to blackish brown.

###### Male.

**Description.** Measurements: HL 1.12–1.18, HW 1.53–1.54, SL 0.41–0.49, EL 0.56–0.60, WL 2.32–2.69, MnW 1.44–1.53, CI 130.6–136.6, SI 26.8–31.8, EI 47.4–53.6, MnI 93.9–99.7 (2 specimens measured).

Eye moderately large, occupying 0.6× head length. Ocelli relatively distant from dorsal margin of head in full-face view. Dorsal margin of head in full-face view weakly rounded. Both anterior and lateral ocelli small. Lateral ocellus relatively small and distant from eye: distance between these more than 2.5× maximum diameter of lateral ocellus. Posterior face of vertex not clearly differentiated from dorsal face, so that margins almost continuously rounded. Palpal formula 4,3. First maxillary palpomere flattened and distinctly wider than second. Second maxillary palpomere longer than third. Notauli shallowly and weakly impressed on mesoscutum, but often unclear. Petiole in dorsal view thin, length 0.6× that of abdominal tergite III. Petiolar dorsum covered with shallow, irregular punctures. Abdominal tergum VIII without deep punctures, almost smooth.

Distal portion of abdominal sternum IX smooth and not punctured. Basal ring moderately long, gently expanding basally. Telomere distinctly extending distally farther than digitus. Basoventral expansion of aedeagus less developed basoventrally, as long as dorsal extension. Ventral margin of aedeagus almost straight in lateral view. Aedeagus distinctly narrowing distally on distal half, but distal portion rounded.

On forewing, cu-a located far basal from junction of Media (M) and Cubitus (Cu).

Body color reddish brown to black.

###### Etymology.

This species name is the English word shadow, inspired by the difficulty of recognizing this new species, which is distributed like *Mystrium voeltzkowi* but hidden in its shadow. The species epithet is a noun and thus invariant.

###### Distribution.

MADAGASCAR: as in Figure 56E.

###### Additional material examined.

In addition to the type material, specimens from the following localities were examined in this study: MADAGASCAR. Antsiranana. Forêt d’Orangea, 3.6 km 128° SE Remena (-12.25889°, 49.37467°), littoral rainforest, 90 m alt.; Parc National Montagne d’Ambre, 3.6 km 235° SW Joffreville (-12.53444°, 49.1795°), montane rainforest, 925 m alt.; Réserve Spéciale de l’Ankarana, 13.6 km 192° SSW Anivorano Nord (-12.86361°, 49.22583°), tropical dry forest, 210 m alt.; Forêt d’ Antsahabe, 11.4 km 275° W Daraina (-13.21167°, 49.55667°), tropical dry forest, 550 m alt.; Forêt de Binara, 9.1 km 233° SW Daraina (-13.26333°, 49.60333°), rainforest, 800 m alt.; 9.4 km 235° SW Daraina (-13.26333°, 49.6°), montane rainforest, 1100 m alt.; Galoko chain, Mont Galoko (-13.58487°, 48.71818°), rainforest, 520 m alt.; (-13.58745°, 48.71419°), rainforest, 380 m alt.; (-13.59358°, 48.73157°), montane forest, 1100 m alt.; Ambondrobe, 41.1 km 175° Vohemar (-13.71533°, 50.10167°), littoral rainforest, 10 m alt.; Ampasindava, Forêt d’Ambilanivy, 3.9 km 181° S Ambaliha (-13.79861°, 48.16167°), rainforest, 600 m alt.; R.S. Manongarivo, 12.8 km 228° SW Antanambao (-13.97667°, 48.42333°), rainforest, 780 m alt.; Forêt Ambanitaza, 26.1 km 347° Antalaha (-14.67933°, 50.18367°), rainforest, 240 m alt.

###### Remarks.

The worker of *Mystrium shadow* is distinguished from other *Mystrium* species by the combination of the following characters: a central longitudinal furrow on the pronotal dorsum (as in Fig. 13B); projected anteromedial portion of the clypeus (Fig. 19A); blunter angle between the dorsal and posterior faces of the vertex on the median line of the head (as in Fig. 16B); the straight posterior declivity of the propodeum (as in Fig. 20). The workers of *Mystrium shadow* are quite similar to those of *Mystrium mirror* and *Mystrium voeltzkowi*, and the differences among the workers of the three species are even slighter in small-sized individuals. When workers in the same body size range are compared, *Mystrium shadow* can be distinguished from *Mystrium mirror* by the central pair of conical setae that is larger in size than the adjacent pair (Fig. 19A), and from *Mystrium voeltzkowi* by a straight declivity of the propodeum (as in Fig. 20). The queen of *Mystrium shadow* can be distinguished from the other *Mystrium* queens by a combination of the mesosoma without developed wing sclerites but with vestigial wing appendages (ergatoid: Fig. 25D), posterior face of the vertex forming a blunt angle with its dorsal face on the median line of the head (Fig. 21B), spatulate setae on dorsum of head and pronotum (Fig. 25B), and the metapleural gland bulla that is moderately developed and not expanding dorsally to the propodeal spiracle (as in Fig. 24B). The ergatoid queens similar to those of *Mystrium shadow* are those of *Mystrium voeltzkowi* and *Mystrium mirror*; however, a less-developed metapleural gland bulla distinguishes *Mystrium shadow* from *Mystrium voeltzkowi* (as in Fig. 24), and spatulate setae on the pronotal dorsum (Fig. 25B) and the presence of the genal tooth of the head (Fig. 54E) distinguish *Mystrium shadow* from *Mystrium mirror*. The male of *Mystrium shadow* is not that similar to that of *Mystrium voeltzkowi* or *Mystrium mirror*. The male of *Mystrium shadow* can be easily separated from the latter two males by the small ocelli (as in Fig. 26B) and small eye (as in Fig. 26D). On the other hand, the male of *Mystrium shadow* is similar to that of *Mystrium janovitzi*. The shape of the vertex is the character that separates these two similar males: the posterior face of the vertex in posterior view is not clearly differentiated from its dorsal face in *Mystrium shadow* (Fig. 32A), while the posterior face of the vertex is clearly divided from its dorsal face on the median line of the head in *Mystrium janovitzi* (Fig. 32B).

This taxonomic revision permits us to update identifications in a recent ecological publication Molet et al. (2009). Material identified as “*Mystrium mysticum*” in their paper is *Mystrium shadow*, not *Mystrium mysticum*.

##### Mystrium voeltzkowi

Forel, 1897

http://species-id.net/wiki/Mystrium_voeltzkowi

Figs 1B , 4B , 5A , 11B , 13B, 13D , 15 , 16B , 19B , 20B, 20D, 20F , 24A , 27A , 27C , 37H , 38H , 39H , 40H , 41H , 50F , 51F , 52F , 53F , 54F , 55F , 56F

Mystrium voeltzkowi Forel, 1897. MADAGASCAR. Syntypes: four workers were confirmed, one male [lectotype is designated below].
Mystrium fallax Forel, 1897. **syn n.** [lectotype is designated below].
Mystrium fallax : Brown 1960; Menozzi 1929.
Mystrium voeltzkowi fallax : Emery 1911.

###### Lectotype of *Mystrium voeltzkowi*

**[here designated].** Worker: CASENT0101952 (the specimen on the bottom side with open mandible out of the two workers on the same pin), MADAGASCAR, Nossi-Bé (Nosy Be), Voeltzkow [MHNG: examined].

###### Lectotype of *Mystrium fallax*

**[here designated].** Ergatoid queen: CASENT0101995, MADAGASCAR, Nossi-Bé (Nosy Be), Voeltzkow [MHNG: same data with lectotype of *Mystrium voeltzkowi*, examined].

###### Worker.

**Description.** Measurements: lectotype. HL 2.08, HW 2.12, SL 1.63, ML 2.67, HD 1.35, WL 2.58, PnW 1.17, PpW 1.00, PtW 0.97, PtL 0.66, CI 102.0, SI 76.9, MI 125.8, PpI 84.9, PtI 147.6.

HL 1.45–2.15, HW 1.41–2.26, SL 1.05–1.65, ML 1.47–2.72, HD 0.96–1.46, WL 1.72–2.68, PnW 0.80–1.20, PpW 0.69–1.09, PtW 0.70–1.03, PtL 0.46–0.67, CI 96.6–104.7, SI 71.7–80.1, MI 104.0–125.7, PpI 86.2–91.0, PtI 141.7–164.6 (9 specimens measured).

Posterolateral corner of head strongly expanding posteriorly. Posterior face of vertex forming blunt angle with dorsal face on median line of the head, so that declivity of vertex on lateral part distinctly steeper than on median part. Ventral half of vertex sculptured. Eye moderately small. Anterior margin of clypeus straight to weakly convex with moderately long conical setae. Genal tooth of head moderately developed, reaching about half of lateral lobe of clypeus. Masticatory surface of mandible in full-face view visible on basal half and invisible on distal half, width of dorsal surface of mandible almost identical from mandibular shaft to distal portion. Second maxillary palpomere longer than third. First flagellomere (third antennal segment) about 1.0–1.3× length of pedicel (second antennal segment). Pronotal dorsum covered with strong to weak longitudinal striae often waved and irregular, but median stria always deeply, straightly and clearly impressed. Shallow, fine longitudinal striae irregularly impressed on lateral surface of pronotum. Mesonotum not differentiated from propodeum in dorsal view in small individuals but differentiated in large individuals, its length shorter than that of propodeum. Metanotal groove indistinct in small individuals and shallowly and gently impressed in large individuals; mesonotum higher than pronotum in lateral view. Metapleural gland bulla strongly developed, so that propodeal declivity in lateral view convex posteriorly on ventral side. Petiole widened on posterior 1/4–1/2, gently narrowed anteriorly in dorsal view, anterior margin straight to gently rounded and not edged by striae.

Body color reddish brown to black. Four distal segments of antennal club brighter.

###### Ergatoid queen.

**Description.** Measurements: HL 1.18-1.59, HW 1.12-1.63, SL 0.88-1.35, ML 1.13-1.64, HD 0.82-1.13, WL 1.56-2.34, PnW 0.70-0.99, PpW 0.70-1.14, PtW 0.68-0.95, PtL 0.38-0.57, CI 95.2-106.4, SI 76.1-85.7, MI 93.1-108.7, PpI 97.1-115.9, PtI 161.8-186.9 (10 specimens measured).

Wings absent or vestigial and reduced to quite small appendages. Wing sclerites undeveloped. Posterolateral corner of head gently expanding posteriorly, expansion distinctly weaker than that in workers. Vertex usually thin, forming blunt angle between dorsal and posterior faces on median line of head, so that declivity of vertex on lateral part distinctly steeper than median part. Ventral half of vertex sculptured, not differentiated from dorsal region. Eye small but distinct. Ocelli absent. Anterior margin of clypeus straight to weakly convex with short conical setae. Genal tooth of head absent, usually even not angular, but angular in smallest individuals. Masticatory margin of mandible almost invisible in full-face view, dorsal surface on distal portion as wide as that on mandibular shaft. Spatulate seta present on basal side of each basal denticle on masticatory margin of mandible. First flagellomere (third antennal segment) moderately long, about 1.1–1.2× length of pedicel (second antennal segment). Setae on pronotum almost simple, narrowing distally with strongly sharpened apex. Metapleural gland bulla extremely developed and expanding dorsally to propodeal spiracle, so that propodeal declivity in lateral view strongly convex posteriorly on ventral 2/3. Petiole relatively long in dorsal view, about 0.5–0.8× length of abdominal segment III.

Body color brown to reddish brown.

###### Male.

**Description.** Measurements: HL 1.27, HW 1.87, SL 0.42, EL 0.91, WL 2.97, MnW 1.72, CI 146.9, SI 22.2, EI 71.4, MnI 92.0 (1 specimen from original type series measured).

Eye quite large, occupying about 0.75× head length. Ocelli protruding from dorsal margin of head in full-face view. Dorsal margin of head in full-face view rounded. Both anterior and lateral ocelli large. Distance between lateral ocellus and eye equal to or shorter than diameter of lateral ocellus. Posterior half of vertex clearly differentiated from dorsal half, dorsal face distinctly shorter than posterior face. Palpal formula 4,3. First maxillary palpomere flattened and distinctly wider than second segment. Second maxillary palpomere longer than third. Notauli shallowly and weakly impressed on mesoscutum, but often unclear. Petiole in dorsal view thin, length 0.55× that of abdominal tergite III. Petiolar dorsum covered with shallow, irregular punctures. Abdominal tergum VIII without deep punctures, almost smooth.

Distal portion of abdominal sternum IX smooth and not punctured. Basal ring moderately long, gently expanding basally. Telomere distinctly extending distally farther than digitus. Basoventral expansion of aedeagus well developed basoventrally, distinctly longer than dorsal extension. Ventral margin of the aedeagus gently curved ventrally in lateral view. Aedeagus distinctly narrowing distally on distal half and apex rounded.

On forewing, cu-a located far basal from junction of Media (M) and Cubitus (Cu).

Body color yellowish to reddish brown.

###### Distribution.

MADAGASCAR and MAYOTTE: as in Figure 56F.

###### Additional material examined.

In addition to the type material, specimens from the following localities were examined in this study: MADAGASCAR. Antsiranana. Réserve Spéciale d’Ambre, 3.5 km 235° SW Sakaramy (-12.46889°, 49.24217°), tropical dry forest, 325 m alt.; Parc National Montagne d’Ambre, 3.6 km 235° SW Joffreville (-12.53444°, 49.1795°), montane rainforest, 925 m alt.; Réserve Spéciale de l’Ankarana, 13.6 km 192° SSW Anivorano Nord (-12.86361°, 49.22583°), tropical dry forest, 210 m alt.; 22.9 km 224° SW Anivorano Nord (-12.90889°, 49.10983°), tropical dry forest, 80 m alt.; Forêt d’Ampondrabe, 26.3 km 10° NNE Daraina (-12.97°, 49.7°), tropical dry forest, 175 m alt.; Forêt d’Analabe, 30.0 km 72° ENE Daraina (-13.08333°, 49.90833°), littoral rainforest, 30 m alt.; Forêt d’ Andavakoera, 21.4 km 75° ENE Ambilobe; 4.6 km 356° N Betsiaka (-13.11833°, 49.23°), rainforest, 425 m alt.; Forêt de Bekaraoka, 6.8 km 60° ENE Daraina (-13.16667°, 49.71°), tropical dry forest, 150 m alt.; Forêt d’ Antsahabe, 11.4 km 275° W Daraina (-13.21167°, 49.55667°), tropical dry forest, 550 m alt.; Nosy Be, Réserve Naturelle Intégrale de Lokobe, 6.3 km 112° ESE Hellville (-13.41933°, 48.33117°), rainforest, 30 m alt.; Forêt d’Anabohazo, 21.6 km 247° WSW Maromandia (-14.30889°, 47.91433°), tropical dry forest, 120 m alt.

MAYOTTE. Mt. Combani (-12.8°, 45.13333°), forest litter, 420 m alt.; (-12.80486°, 45.15266°), rainforest, 460 m alt.; (-12.80632°, 45.15314°), rainforest, 370 m alt.; Dapani (-12.96279°, 45.15037°), rainforest, 135 m alt.

###### Remarks.

The worker of *Mystrium voeltzkowi* is distinguished from other *Mystrium* workers by the combination of the following characters: the vertex forming a blunt angle between posterior and dorsal faces on the median line of the head (Fig. 16B), small compound eye (Fig. 20D), the genal tooth of the head reaching to half of the lateral lobe of the clypeus (Fig. 20B), and a medial pair of conical setae on the clypeus that is smaller than adjacent pair (Fig. 19B). In addition, the characters of a central longitudinal furrow on the pronotal dorsum (Fig. 13B) but without deep, stout striae impressed on dorsal and lateral surface of pronotum, and the posterior declivity of the propodeum convex posteriorly on the ventral half (Fig. 20F), so that the margin is somewhat rounded in lateral view, are also useful. The workers of *Mystrium mirror* and *Mystrium shadow* are quite similar to those of *Mystrium voeltzkowi*, and the differences among the three species in workers are even slighter in small-sized individuals. When the comparison is made in workers within the same body size range, *Mystrium voeltzkowi* can be distinguished from *Mystrium shadow* by the small central clypeal conical setae (Fig. 19B), and from *Mystrium mirror* by either a rounded declivity of the propodeum (Fig. 20F) or smaller compound eye (Fig. 20D). The queen of *Mystrium voeltzkowi* is easily distinguished from the other *Mystrium* queens by the mesosoma without developed wing sclerites (ergatoid: as in Fig. 25C), the developed metanotal gland bulla expanding dorsally to the propodeal spiracle (Fig. 24A), and the brighter body color. Among males, only genital characters distinguish *Mystrium voeltzkowi* from *Mystrium mirror* (Fig. 27).

The male of *Mystrium voeltzkowi* was described with conspecific workers from the same colony in the original description (Forel 1897). We confirmed one male in the Forel collection in MHNG; its pin has the same collection label as the other syntypes. This is the only reliable male description in all previous male descriptions of the genus *Mystrium*.

*Mystrium fallax* Forel, 1897 is synonymized with *Mystrium voeltzkowi* in this study. *Mystrium fallax* was apparently described by a simple mistake. According to the original description of *Mystrium voeltzkowi* by Forel (1897), he received a vial containing workers, two other reddish individuals, and males which were collected in Nossi-Bé by Dr. Voeltzkow. When Forel (1897) described the worker and male as *Mystrium voeltzkowi*, he tentatively regarded the two reddish individuals in the same vial as a different species due to the distinct differences in their appearance (as Figs 50F vs. 53F), and named these specimens *Mystrium fallax*. Forel doubted that the two small reddish individuals belonged to the same colony as *Mystrium voeltzkowi*; and later Menozzi (1929) even determined these to be a species distinct from *Mystrium voeltzkowi*. However, the type specimens of *Mystrium fallax* were probably from the same colony of types of *Mystrium voeltzkowi*. The mistake was due to the remarkable polymorphism in this species. Once the original descriptions of *Mystrium fallax* and *Mystrium voeltzkowi* were published, the association between the large black worker (Fig. 50F) and the small reddish individuals (Fig. 53F) was not clarified for more than 110 years. Now accumulated material clearly shows the small reddish individuals are conspecific with the black large worker, and an ecological study (Molet et al. 2007a) revealed these small reddish individuals are ergatoid queens of this strange species (Fig. 1B).

The distribution of *Mystrium voeltzkowi* is limited to Mayotte and the northern part of Madagascar (Fig. 56F). The collection localities of *Mystrium mirror* are in tropical dry forest, gallery forest, and spiny forest (Fig. 56C), while those of *Mystrium voeltzkowi* are in tropical dry forest, montane rainforest, littoral rainforest, and rainforest (Fig. 56F). The distinct development of the metapleural grand bulla observed in *Mystrium voeltzkowi* (Figs 20F, 24A) may be related to its nesting environment, which has higher moisture levels and requires stricter control of fungi.

The reference to *Mystrium voeltzkowi* in recent molecular phylogenetic studies (Brady et al. 2006; Kück et al. 2011; Moreau and Bell 2013; Ouellette et al. 2006; Saux et al. 2004) and ecological studies (Molet et al. 2009; Molet et al. 2007a) need to be reassessed. Material identified as *Mystrium voeltzkowi* in Saux et al. (2004) are confirmed here. *Mystrium mysticum* material in Brady et al. (2006), Ouellette et al. (2006), Kück et al. (2011), and Moreau and Bell (2013) is actually *Mystrium voeltzkowi*. This complicated species also has been represented by species codes as *Mystrium* ‘red’ in Molet et al. (2009), Molet et al. (2007a), and Molet et al. (2012), as *Mystrium* sp.2 in Saux et al. (2004), and as *Mystrium* mysticum2 in Ouellette et al. (2006). These complications represent the typical difficulty of species-level identification in *Mystrium*.

## Supplementary Material

XML Treatment for Mystrium

XML Treatment for Mystrium barrybressleri

XML Treatment for Mystrium camillae

XML Treatment for Mystrium labyrinth

XML Treatment for Mystrium mysticum

XML Treatment for Mystrium rogeri

XML Treatment for Mystrium eques

XML Treatment for Mystrium janovitzi

XML Treatment for Mystrium mirror

XML Treatment for Mystrium oberthueri

XML Treatment for Mystrium shadow

XML Treatment for Mystrium voeltzkowi

## Supplementary material

Supplementary material 1 A data sheet for all specimens of *Mystrium* examined doi: 10.3897/zookeys.394.6446.app1 Data type: occurrences Explanation note: Comprehensive data for all *Mystrium* specimens examined including information of specimens ID, species name, sex and caste, collection data, and depository. This dataset is made available under the Open Database License (http://opendatacommons.org/licenses/odbl/1.0/). The Open Database License (ODbL) is a license agreement intended to allow users to freely share, modify, and use this Dataset while maintaining this same freedom for others, provided that the original source and author(s) are credited.Masashi Yoshimura, Brian L. Fisher

## References

[B1] Bihn JH Verhaagh M (2007) A review of the genus *Mystrium* (Hymenoptera: Formicidae) in the Indo-Australian region. Zootaxa 1642: 1-12.

[B2] Bolton B (1994) Identification guide to the ant genera of the world. Harvard University Press, Cambridge, Massachusetts, 222 pp.

[B3] Bolton B (2003) Synopsis and classification of Formicidae. Memoirs of the American Entomological Institute 71: 1-370.

[B4] Bouchet DC Peeters C Fisher BL Molet M (2013) Both female castes contribute to colony emigration in the polygynous ant *Mystrium oberthueri*. Ecological Entomology 38: 408-417. doi: 10.1111/een.12033

[B5] Brady SG Schultz TR Fisher BL (2006) Evaluating alternative hypotheses for the early evolution and diversification of ants. Proceedings of the National Academy of Sciences of the United States of America 103: 18172-18177. doi: 10.1073/pnas.0605858103 17079492 PMC1838725

[B6] Brown WL Jr. (1960) Contributions toward a reclassification of the Formicidae. III. Tribe Amblyoponini (Hymenoptera). Bulletin of the Museum of Comparative Zoology 122: 143-230.

[B7] Emery C (1889) Formiche di Birmania e del Tenasserim raccolte da Leonardo Fea (1885–87). [part]. Annali del Museo Civico di Storia Naturale di Genova 27: 485-512.

[B8] Emery C (1895) Die Gattung Dorylus Fab. und die systematische Eintheilung der Formiciden. Zoologische Jahrbücher, Abteilung für Systematik, Geographie und Biologie der Tiere 8: 685–778, Taf. 614–617.

[B9] Emery C (1899) Formiche di Madagascar raccolte dal Sig. A. Mocquerys nei pressi della Baia di Antongil (1897–1898). Bullettino della Societa Entomologica Italiana 31: 263-290.

[B10] Emery C (1911) Hymenoptera. Fam. Formicidae. Subfam. Ponerinae. Brussels, 1–125, pl. 121–123 pp.

[B11] Fisher BL (2005) A model for a global Inventory of ants: A case study in Madagascar. In:Jablonski NG(Ed) Biodiversity: A Symposium Held on the Occasion of the 150th Anniversary of the California Academy of Sciences June 17–18, 2003. Proceedings of the California Academy of Sciences, San Francisco, California, 78–89 pp.

[B12] Fisher BL (2010) Biogeography. In: Lach L Parr C Abbott K (Eds) Ant Ecology. Oxford University Press, Oxford, UK, 18–37, references 319–383.

[B13] Forel A (1891) Les Formicides. In: Grandidier A (Ed) Histoire physique, naturelle, et politique de Madagascar Histoire naturelle des Hyménoptères Deuxième partie. Hachette et Cie, Paris, v + 280, pl. I-VII.

[B14] Forel A (1892) Nouvelles espèces de Formicides de Madagascar (récoltées par M. Sikora). Première série. Annales de la Société Entomologique de Belgique 36: 516-535.

[B15] Forel A (1893) Sur la classification de la famille des Formicides, avec remarques synonymiques. Annales de la Société Entomologique de Belgique 37: 161-167.

[B16] Forel A (1895) Nouvelles fourmis de l’Imerina oriental (Moramanga etc.). Annales de la Société Entomologique de Belgique 39: 243-251.

[B17] Forel A (1897) Ameisen aus Nossi-Bé, Majunga, Juan de Nova (Madagaskar), den Aldabra-Inseln und Sansibar, gesammelt von Herrn Dr. A. Voeltzkow aus Berlin. Mit einem Anhang über die von Herrn Privatdocenten Dr. A. Brauer in Marburg auf den Seychellen und von Herrn Perrot auf Ste. Marie (Madagaskar) gesammelten Ameisen. Abhandlungen der Senckenbergischen Naturforschenden Gesellschaft 21: 185-208.

[B18] Forel A (1899) Trois notices myrmécologiques. Annales de la Société Entomologique de Belgique 43: 303-310.

[B19] Gauld I Bolton B (1988) The Hymenoptera. Oxford University Press, Oxford, xii + 322 pp.

[B20] Gronenberg W Hölldobler B Alpert GD (1998) Jaws that snap: control of mandible movements in the ant *Mystrium*. Journal of Insect Physiology 44: 241-253. doi: 10.1016/S0022-1910(97)00145-5 12769958

[B21] Hita Garcia F Fisher BL (2012) The ant genus *Tetramorium* Mayr (Hymenoptera: Formicidae) in the Malagasy region—taxonomic revision of the *T. kelleri* and *T. tortuosum* species groups. Zootaxa 3592: 1-85. 10.3897/zookeys.413.7172 PMC4086027 25009414

[B22] Huber JT Sharkey MJ (1993) Structure. In: Goulet H Huber TJ (Eds) Hymenoptera of the world: an identification guide to families. Research Branch Agriculture Canada Publication 1894/E, Ottawa: 13-59.

[B23] Karavaiev V (1925) Ponerinen (Fam. Formicidae) aus dem Indo-Australischen Gebiet. Konowia 4: 69-81.

[B24] Keller RA (2011) A phylogenetic analysis of ant morphology (Hymenoptera: Formicidae) with special reference to the poneromorph subfamilies. Bulletin of the American Museum of Natural History 355: 1-90. doi: 10.1206/355.1

[B25] Kück P Hita Garcia F Misof B Meusemann K (2011) Improved phylogenetic analyses corroborate a plausible position of *Martialis heureka* in the ant tree of life. PLoS ONE 6(6): e21031: 15 p. doi: 10.1371/journal.pone.0021031 PMC3123331 21731644

[B26] Mayr G (1862) Myrmecologische Studien. Verhandlungen der Kaiserlich-Königlichen Zoologisch-Botanischen Gesellschaft in Wien 12: 649-776.

[B27] Menozzi C (1929) Revisione delle formiche del genere *Mystrium* Roger. Zoologischer Anzeiger 82: 518-536.

[B28] Molet M Fisher BL Ito F Peeters C (2009) Shift from independent to dependent colony foundation and evolution of ‘multi-purpose’ ergatoid queens in *Mystrium* ants (subfamily Amblyoponinae). Biological journal of the Linnean Society 98: 198-207. doi: 10.1111/j.1095-8312.2009.01257.x

[B29] Molet M Peeters C Fisher BL (2007a) Winged queens replaced by reproductives smaller than workers in *Mystrium* ants. Naturwissenschaften 94: 280-287. doi: 10.1007/s00114-006-0190-2 17165079

[B30] Molet M Peeters C Follin I Fisher BL (2007b) Reproductive caste performs intranidal tasks instead of workers in the ant *Mystrium oberthueri*. Ethology 113: 721-729. doi: 10.1111/j.1439-0310.2007.01376.x

[B31] Molet M Wheeler DE Peeters C (2012) Evolution of novel mosaic castes in ants: modularity, phenotypic plasticity, and colonial buffering. American Naturalist 180: 328-341. doi: 10.1086/667368 22854076

[B32] Moreau CS Bell CD (2013) Testing the museum versus cradle tropical biological diversity hypothesis: phylogeny, diversification, and ancestral biogeographic range evolution of the ants. Evolution 67: 2240-2257. doi: 10.1111/evo.12105 23888848

[B33] Moreau CS Bell CD Vila R Archibald SB Pierce NE (2006) Phylogeny of the ants: diversification in the age of angiosperms. Science 312: 101-104. doi: 10.1126/science.1124891 16601190

[B34] Ouellette GD Fisher BL Girman DJ (2006) Molecular systematics of basal subfamilies of ants using 28S rRNA (Hymenoptera: Formicidae). Molecular Phylogenetics and Evolution 40: 359–369. doi: 10.1016/j.ympev.2006.03.017 16630727

[B35] Roger J (1862) Einige neue exotische Ameisen-Gattungen und Arten. Berliner Entomologische Zeitschrift 6: 233-254.

[B36] Santschi F (1914) Formicides de l’Afrique occidentale et australe du voyage de M. le Professeur F. Silvestri. Bollettino del Laboratorio di Zoologia Generale ed Agraria, Portici 8: 309–385.

[B37] Saux C Fisher BL Spicer GS (2004) Dracula ant phylogeny as inferred by nuclear 28S rDNA sequences and implications for ant systematics (Hymenoptera: Formicidae: Amblyoponinae). Molecular Phylogenetics and Evolution 33: 457-468. doi: 10.1016/j.ympev.2004.06.017 15336679

[B38] Serna F Mackay W (2010) A descriptive morphology of the ant genus *Procryptocerus* (Hymenoptera: Formicidae). Journal of Insect Science 10: 137: 36 p. doi: 10.1673/031.010.11101, http://www.insectscience.org/10.111/ PMC3016927 20874568

[B39] Snodgrass RE (1935) Principles of Insect Morphology, with a new foreword by G. C Eickwort. (1993). Cornell University Press, New York, 1–86 +33 pls pp.

[B40] Snodgrass RE (1941) The male genitalia of Hymenoptera. Smithsonian Miscellaneous Collections 99: 1-86.

[B41] Snodgrass RE (1957) A revised interpretation of the external reproductive organs of male insects. Smithsonian Miscellaneous Collections 135: 1-60.

[B42] Ward PS (1994) *Adetomyrma*, an enigmatic new ant genus from Madagascar (Hymenoptera: Formicidae) and its implications for ant phylogeny. Systematic Entomology 19: 159-175. doi: 10.1111/j.1365-3113.1994.tb00585.x

[B43] Wootton RJ (1979) Function, homology and terminology in insect wings. Systematic Entomology 4: 81-93. doi: 10.1111/j.1365-3113.1979.tb00614.x

[B44] Xu ZH (1998) Two new species of the genera *Mystrium* and *Cryptopone* from Yunnan, China (Hymenoptera: Formicidae). Zoological Research 19: 160-164.

[B45] Xu ZH Chu JJ (2012) Four new species of the amblyoponine ant genus *Amblyopone* (Hymenoptera: Formicidae) from Southwestern China with a key to the known Asian species. Sociobiology 59: 1175-1196.

[B46] Yoshimura M Fisher BL (2007) A revision of male ants of the Malagasy region (Hymenoptera: Formicidae): Key to subfamilies and treatment of the genera of Ponerinae. Zootaxa 1654: 21-40.

[B47] Yoshimura M Fisher BL (2009) A revision of male ants of the Malagasy region (Hymenoptera: Formicidae): Key to genera of the subfamily Proceratiinae. Zootaxa 2216: 1-21.

[B48] Yoshimura M Fisher BL (2011) A revision of male ants of the Malagasy region (Hymenoptera: Formicidae): Key to genera of the subfamily Dolichoderinae. Zootaxa 2794: 1-34.

[B49] Yoshimura M Fisher BL (2012) A revision of male ants of the Malagasy Amblyoponinae (Hymenoptera: Formicidae) with resurrections of the genera *Stigmatomma* and *Xymmer*. PLoS ONE 7: e33325. doi: 10.1371/journal.pone.0033325 22496722 PMC3320654

